# DDX39B K63-linked ubiquitination mediated by TRIM28 promotes NSCLC metastasis by enhancing ECAD lysosomal degradation

**DOI:** 10.1038/s41392-025-02305-9

**Published:** 2025-07-16

**Authors:** Hang Yuan, Qin Li, Liang Li, Gang Zhao, Jie Zhang, Tianyu Feng, Yafei Guo, Qiming Kou, Siqi Li, Shan Li, Minghui Zhao, Guanru Wang, Qijing Wang, Jie Qu, Huayang Yu, Hongbai Chen, Lunxu Liu, Kai Li, Ping Lin

**Affiliations:** 1 Department of Thoracic Surgery and Lab of Experimental Oncology, State Key Laboratory of Biotherapy, West China Hospital, Sichuan University, Chengdu, China; 2 Division of Abdominal Tumor Multimodality Treatment, Cancer Center and Lab of Experimental Oncology, State Key Laboratory of Biotherapy, West China Hospital, Sichuan University, Chengdu, China; 3 Key Laboratory of Birth Defects and Related Diseases of Women and Children of MOE, Department of Pediatrics, West China Second University Hospital, Sichuan University, Chengdu, China; 4 Department of Thoracic Surgery, West China Hospital, Sichuan University, Chengdu, China

**Keywords:** Lung cancer, Metastasis, Oncogenes, Cell biology, Cancer therapy

## Abstract

Metastasis is a leading cause of treatment failure and high mortality in non-small cell lung cancer (NSCLC). Recently, we demonstrated that DEAD box helicase 39B (DDX39B) was upregulated and activated metabolic reprogramming in colorectal cancer and hepatocellular carcinoma. However, the function of DDX39B and the therapeutic potential for targeting DDX39B in NSCLC remain unclear. Herein, we discovered that DDX39B was an independent marker for poor survival in NSCLC patients. Strikingly, DDX39B protein, but not its mRNA, was elevated in clinical metastatic brain lesions and metastatic cell models (in vitro EMT-metastatic and in vivo carotid artery injection-induced brain-metastatic cell model). Mechanistically, DDX39B interacted with E3 ubiquitin ligase TRIM28 via Pro322 residue and underwent TRIM28-mediated K63-linked ubiquitination at Lys241, Lys384, and Lys398, leading to DDX39B protein stabilization and upregulation. Subsequently, DDX39B directly bound to ECAD and promoted ECAD lysosomal degradation by recruiting Src and Hakai, which was independent of its RNA helicase activity, followed by activating β-catenin oncogenic signaling and facilitating NSCLC aggressive phenotype. According to structure-based virtual screening, we discovered a clinical antimalarial drug, artesunate, that disrupted the association of DDX39B-TRIM28 complex, resulting in DDX39B degradation and blocking the pro-metastatic effects of DDX39B. Overall, our findings uncover that TRIM28/DDX39B/ECAD axis contributes to NSCLC metastasis and targeting DDX39B degradation by artesunate is an effective and promising therapeutic approach for the treatment of NSCLC.

## Introduction

Lung cancer is a severe burden on human health and has become the leading cause of cancer-related mortality worldwide.^1^ Non-small cell lung cancer (NSCLC) is the primary histological subtype of lung cancer accounting for approximately 85% of lung cancer cases and up to 60% of patients with NSCLC are diagnosed at advanced stages due to the underexploitation of early detection and surveillance.^2^ Metastasis is the leading cause of the lung cancer-associated deaths. Brain is the most predominant secondary metastasis site in lung cancer and approximately half of NSCLC eventually develop into brain metastasis (BM).^3^ Despite clinical advances in the treatment of NSCLC (such as tyrosine kinase inhibitors and immunotherapy), the prognosis of NSCLC patients with BM remains poor, with an average median period no more than 6 months.^4^ The metastatic cascade encompasses a long series of sequential and interrelated steps, including cellular transformation and tumor growth, extensive vascularization, local invasion, detachment, circulation, extravasation, and proliferation within distant organs.^5^ Unlike breast cancer and colorectal carcinoma, NSCLC cells enable to rapidly acquire both infiltration and colonization competence, as evidenced by the short time between primary tumor diagnosis and metastatic relapses in disease progression.^6^ Importantly, NSCLC cells undergoing metastasis usually exhibit heterogeneity and plasticity resembling epithelial‒mesenchymal transition (EMT), which results in limited clinical response and treatment failure in advanced NSCLC patients.^7,8^ Therefore, clarifying the detailed molecular mechanisms that drive the malignant progression of NSCLC will identify effective metastatic biomarkers and potential treatment strategies, ultimately aiming to enhance precision prevention and improve the outcomes of NSCLC patients.

DExD-box helicase 39B (DDX39B, also known as BAT1 and UAP56), a member of DEAD box (DDX) RNA helicase family, has been demonstrated to participate in the development of various diseases through the regulation of mRNA transcription, splicing and nuclear export.^9–11^ In addition to their canonical RNA regulatory functions, DDX family has recently been confirmed to participate in tumor development via protein‒protein interaction. For example, DDX24 disassociated p300-p53 interplay to repress p300-mediated acetylation of p53, which inactivated p53 transcriptional activity and encouraged the proliferation in human cancer cells.^12^ DDX21 cooperatively induced ULK1 gene transcription through recruitment of transcription factor YBX1, resulting in the aggressive progression of acute myeloid leukaemia.^13^ Our previous study demonstrated that DDX39B directly interacted with SREBP1 and stabilized SREBP1 protein by preventing FBXW7-mediated ubiquitination and degradation of SREBP1, followed by the nuclear translocation of SREBP1 and de novo lipid synthesis in hepatocellular carcinoma (HCC).^14^ Moreover, DDX39B bound to retinol saturase to remove R-loops and promote fork restarting, leading to cellular resistance to gemcitabine- and hypoxia-induced replication stress in pancreatic ductal adenocarcinoma.^15^ However, the function, expression regulation and clinical significance of DDX39B in NSCLC remain unknown.

Tripartite motif-containing 28 (TRIM28) belongs to TRIM E3 ubiquitin ligase family. Accumulating evidence has reported the highly complex functions of TRIM28 participated in many aspects of cancer cell biology via regulation of gene transcription, induction of autophagy, and maintenance of stem cell pluripotency, etc.^16^ TRIM28 appears ubiquitous and highly expressed in several types of tumors. A recent study has reported that TRIM28 SUMOylated NUP37 at Lys114/118/246 to inhibit K27-linked polyubiquitination of NUP37, which augmented NUP37 protein level and accelerated lipid synthesis and the progression of HCC.^17^ TRIM28 directly interacted with PD-L1 and prevented PD-L1 degradation through inhibition of PD-L1 ubiquitination. On the other hand, TRIM28 increased PD-L1 transcription by inducing K63-linked polyubiquitination of TBK1, leading to the escape of gastric cancer cells.^18^ In NSCLC, TRIM28 was upregulated in tumor tissues compared with adjacent normal tissues and facilitated the degradation of RLIM, an MDM2 negative modulator, which maintained low p53 protein expressions and in turn promoted lung tumorigenesis.^19^ Paradoxically, Chen and colleagues revealed that high levels of TRIM28 protein were correlated with better overall survival in a panel of early-stage lung adenocarcinomas and that TRIM28 hindered cell proliferation via interference of the transcriptional activity of E2F3 and E2F4.^20^ These findings suggest multiple roles of TRIM28 in different stages of NSCLC development. Moreover, the specific substrates of TRIM28 are not fully understood.

Here, we provided the first evidence that DDX39B protein expression was gradually upregulated from paracancerous tissue to primary tumor tissue to metastatic NSCLC tissue and that elevated DDX39B protein level was an independent indicator of poor overall survival in patients with NSCLC. We also observed increased expression of DDX39B protein, but not its mRNA, in EGF- or TGF-β-induced EMT-metastatic model and carotid artery injection-induced BM model. Mechanistically, our findings confirmed that DDX39B was a novel substrate of E3 ubiquitin ligase TRIM28, which facilitated K63-linked ubiquitination of DDX39B at Lys241, Lys384, and Lys398, resulting in the stabilization and upregulation of DDX39B protein during metastasis process. Subsequently, DDX39B recruited Src and Hakai to promote the lysosomal degradation of E-cadherin (ECAD), followed by the dissociation of β-catenin from the β-catenin/ECAD complex, β-catenin nuclear translocation and activation of downstream oncogenic signaling pathways. By performing structure-based virtual screening, we discovered a clinical antimalarial drug artesunate that enabled to disrupt the association of DDX39B-TRIM28 complex, leading to the decreased expression of DDX39B, and that neutralized the pro-EMT reprogramming and pro-metastatic effects of DDX39B in NSCLC. Our current study expands molecular insight underlying the regulation of DDX39B expression at post-translational level and DDX39B is a feasible and beneficial target of pharmacological strategies for NSCLC therapy.

## Results

### Upregulated DDX39B protein is correlated with metastasis and poor prognosis in NSCLC

**Fig. 1:**
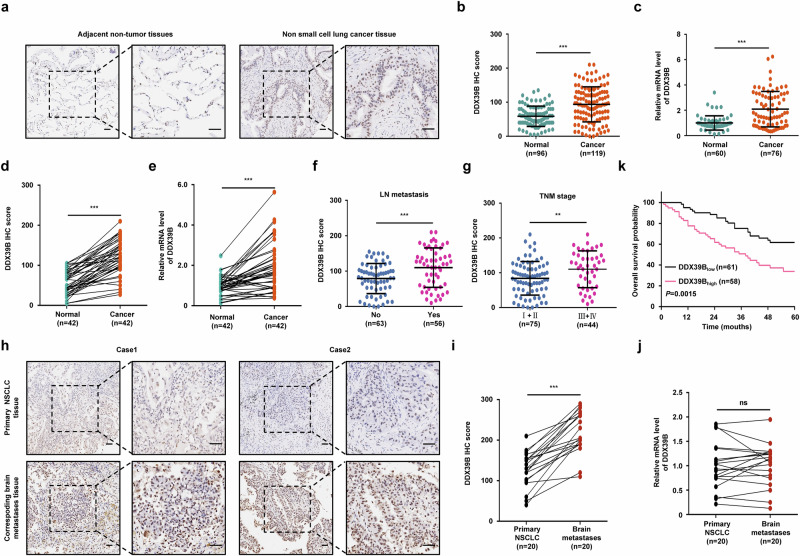
Upregulated DDX39B correlates with metastasis and predicts poor prognosis in NSCLC patients. The protein expression of DDX39B in NSCLC (*n* = 119) and adjacent non-tumor tissues (*n* = 96) was determined by immunohistochemistry analysis, (**a**) representative images and (**b**) relative intensities of DDX39B were shown. **c** The mRNA expression of DDX39B in NSCLC (*n* = 76) and adjacent non-tumor tissues (*n* = 60) was measured by RT‒qPCR assay. **d** The relative intensities of DDX39B protein staining or (**e**) mRNA level were analyzed in primary NSCLC and patient-matched normal tissues (*n* = 42). **f** DDX39B protein expression in primary NSCLC tissues with lymph node (LN) metastasis (*n* = 56) or without LN metastasis (*n* = 63). **g** DDX39B protein expression in primary NSCLC tissues at early (I + II) (*n* = 75) or advanced (III + IV) (*n* = 44) TNM stages. DDX39B expression in metastatic brain tissues and matched primary NSCLC tissues (*n* = 20) was determined by (**h**, **i**) immunohistochemistry analysis or (**j**) RT‒qPCR. **h** Representative images of DDX39B expression in primary tissue and metastatic brain lesions were shown. **i** The relative intensities of DDX39B protein staining or (**j**) mRNA level were analyzed. **k** Kaplan‒Meier estimates of overall survival probability based on DDX39B protein expression in NSCLC patients (*n* = 119). Scale bars, 50 µm. *P* values were determined by using the two-tailed unpaired *t* test (**b**, **c**, **f**, **g**), two-tailed paired *t* test (**d**, **e**, **i**, **j**), or log-rank test (**k**). ns, not significant, **P < 0.01, ***P < 0.001

To clarify the clinical relevance of DDX39B in NSCLC, we first determined the protein expression of DDX39B via our tissue microarray, which contained 119 NSCLC specimens and 96 adjacent normal lung specimens. DDX39B protein levels were significantly augmented in cancer tissues compared with normal tissues (Fig. 1a, b**)**. Consistent results were obtained from the CPTAC database by an online tool (http://ualcan.path.uab.edu/index.html) (Supplementary Fig. 1a, b). We also found higher expression of DDX39B mRNA in NSCLC tissues than paracancerous tissues (Fig. 1c). We subsequently analyzed DDX39B level in 42 samples of primary NSCLC and patient-matched normal tissues. Both the mRNA and protein expression of DDX39B were increased in NSCLC tissues compared with paired adjacent noncancerous tissues(Fig. 1d, e). Moreover, elevated DDX39B protein was observed in primary NSCLC tissues of patients with lymph node metastasis compared with those without lymph node metastasis (Fig. 1f). When tumors were grouped by TNM stage, NSCLC patients with late-stage disease (stage III + IV) showed higher expression of DDX39B than those with early-stage disease (stage I + II) (Fig. 1g). According to the clinicopathologic features, DDX39B expression was associated with lymph node metastasis, M stage, and TNM stage in NSCLC patients (Supplementary Table 1). Considering that up to 50% of patients with NSCLC clinically develop brain metastasis,^21^ we further examined the DDX39B expression in NSCLC BM tissues. Strikingly, DDX39B protein, but not its mRNA, was shown to be further increased in metastatic brain lesions compared with patient-matched primary tumors (Fig. 1h–j). The outcome was significantly worse in NSCLC patients with high DDX39B protein expression than in those with low DDX39B expression (Fig. 1k). Multivariate analysis showed that DDX39B level, lymph node metastasis and TNM stage were independent risk factors for predicting poor overall survival in NSCLC patients (Supplementary Table 2). Collectively, these data indicate that upregulated DDX39B protein level is correlated with aggressive progression and unfavorable prognosis in NSCLC patients.

### DDX39B protein is highly expressed in multiple metastasis models

**Fig. 2:**
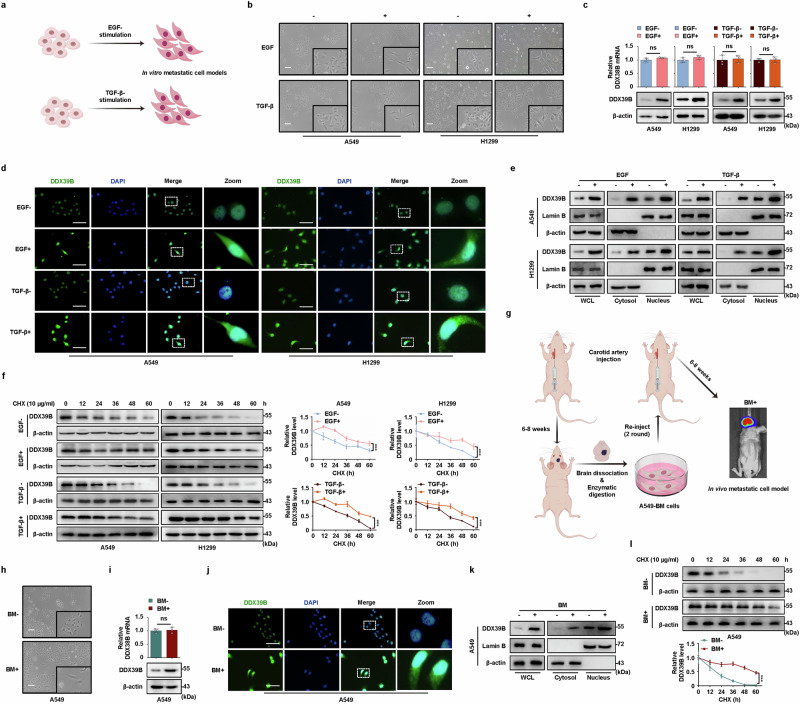
DDX39B protein is highly expressed during metastasis in multiple metastasis models. **a** Scheme for the in vitro establishment of EGF- or TGF-β-induced EMT cell models (by Figdraw). **b**–**e** NSCLC cell lines (A549 and H1299) were starved overnight and then treated with or without 30 ng/ml EGF or 10 ng/ml TGF-β for 72 h. **b** Cell morphology was analyzed using microscopy. **c** The expression of DDX39B was determined by RT‒qPCR (*n* = 3) and Western blotting. **d** The expression and distribution of DDX39B protein were examined by immunofluorescence staining. **e** DDX39B expression in the indicated cell fractions was detected by Western blot analysis. WCL: whole cell lysates. **f** NSCLC cells were starved overnight and treated with 10 µg/ml cycloheximide (CHX) for the indicated times (0, 12, 24, 36, 48 and 60 h) in the absence or presence of EGF (30 ng/ml) or TGF-β (10 ng/ml). Cell lysates were immunoblotted with antibodies against DDX39B and β-actin, and the ratio of DDX39B to β-actin was calculated. The line graph represents the rate of DDX39B degradation (*n* = 3). **g** Scheme for the in vivo selection process of the isolation of brain-metastasis (BM) cells from A549-luc cells (by Figdraw). **h** Cell morphology of A549-parental and BM cell lines. **i** The expression of DDX39B in A549-parental and BM cell lines was determined by RT‒qPCR (*n* = 3) and Western blotting. **j** The expression and distribution of DDX39B protein in A549-parental and BM cell lines were examined by immunofluorescence staining. **k** DDX39B expression in the indicated cell fractions of A549-parental and BM cells was detected by Western blot analysis. WCL: whole cell lysates. **l** A549-parental and BM cells were treated with 10 µg/ml CHX for the indicated times (0, 12, 24, 36, 48 and 60 h). Cell lysates were immunoblotted with antibodies against DDX39B and β-actin, and the ratio of DDX39B to β-actin was calculated. The line graph represents the rate of DDX39B degradation (*n* = 3). Scale bars, 50 µm. Graphs represent data as the mean±s.d. *P* values were determined by a two-tailed unpaired *t* test (**c**, **i**) or two-way ANOVA (**f**, **l**). ns, not significant, **P < 0.01, ***P < 0.001

Considering that EMT was an essential process during metastasis, we first constructed EGF- or TGF-β-induced EMT-metastatic models^22–24^ to investigate the expression regulation of DDX39B during metastasis (Fig. 2a). As expected, compared with control cells, EGF-treated and TGF-β-treated NSCLC cells lost their cobblestone morphology and acquired a spindle-shaped pattern with motivated F-actin rearrangement, a lower level of the epithelial marker ECAD and a higher level of the mesenchymal marker Vimentin (Fig. 2b and Supplementary Fig. 2a, b). These data confirmed the successful establishment of the EMT-metastasis model in NSCLC cells. DDX39B was significantly increased at the protein level but not at the mRNA level in NSCLC cells upon exposure to EGF or TGF-β (Fig. 2c). Interestingly, the cytoplasmic distribution of DDX39B protein was increased in the EMT-metastasis model, as validated by immunofluorescence staining and cell fraction assays (Fig. 2d, e). These results suggested that the upregulation of DDX39B protein during metastasis might be attributed to the elevated stabilization of DDX39B protein. To verify this hypothesis, a CHX (cycloheximide, an inhibitor of protein synthesis) assay revealed that the degradation of DDX39B protein was remarkably delayed during EGF- or TGF-β-induced metastasis process (Fig. 2f). Considering that brain metastasis is a major determinant of prognosis for NSCLC patients, we subsequently established a BM model through carotid artery inoculation of A549 cells into nude mice as done previously^25^ (Fig. 2g). After three rounds of in vivo selection, A549-BM cell populations exhibited enriched metastatic activity, as validated by bioluminescence imaging and H&E-stained BM lesion sections (Fig. 2g and Supplementary Fig. 3a). As shown in Fig. 2h, A549-BM cell lines exhibited a more mesenchymal morphology than A549-parental cells. In addition, the enhanced migration, invasiveness and 3D sphere formation activities were observed in A549-BM cells compared to parent A549 cells (Supplementary Fig. 3b, c). Consistent with our in vitro findings, the expression, cytoplasmic accumulation and stability of DDX39B protein were obviously higher in derivative cells than those in parent cells (Fig. 2i–l). Thus, our results reveal that post-translational modulation contributes to the elevation of DDX39B protein during NSCLC metastasis process.

### DDX39B is a direct substrate of the E3-specific ligase TRIM28

**Fig. 3:**
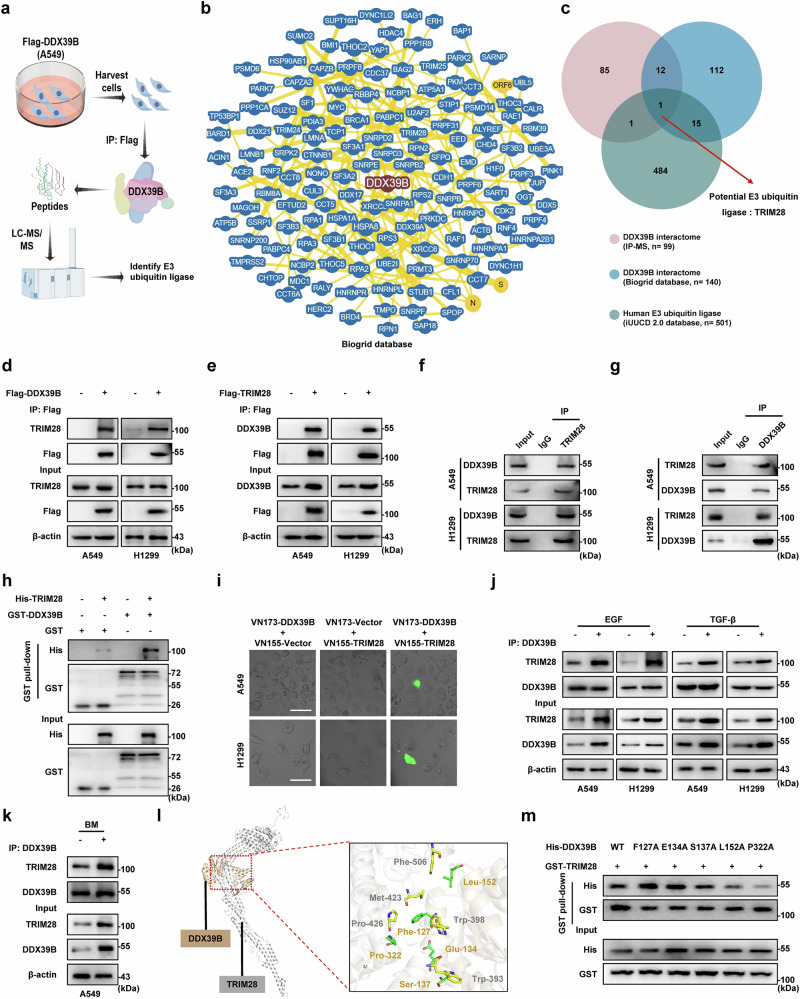
DDX39B interacts with TRIM28 via P322 residue. **a** Schematic workflow for exploring DDX39B interactors by co-IP coupled with LC-MS/MS (by Figdraw). **b** Protein‒protein interaction network of DDX39B from the Biogrid database (https://thebiogrid.org/, minimum evidence: 10). **c** Venn diagram shows the overlap of potential E3 ligase (TRIM28) identified from our DDX39B interactors data, Biogrid database, and iUUCD 2.0 database. **d**, **e** Exogenous and (**f**, **g**) endogenous associations between DDX39B and TRIM28 were determined by immunoprecipitation analysis in NSCLC cell lines (A549 and H1299). **h** GST pull-down assay of purified GST-DDX39B and His-TRIM28 proteins. **i** Indicated BiFC plasmids were transfected into NSCLC cells, in vivo interaction between DDX39B and TRIM28 was detected by BiFC analysis. **j** NSCLC cell lines were treated with EGF (30 ng/ml) or TGF-β (10 ng/ml) for 72 h, the interaction between DDX39B and TRIM28 was examined. **k** The interaction between DDX39B and TRIM28 was examined in A549-parental and BM cell lines. **l** The binding interface between DDX39B and TRIM28 protein was shown. **m** GST pull-down assay of purified GST-TRIM28 and the WT or the indicated mutant forms of His-DDX39B. Scale bars, 50 µm

Considering that E3 ubiquitin ligases play important roles in the regulation of protein expression and stability, we performed immunoprecipitation-mass spectrum (IP-MS) analysis and identified 99 potential DDX39B-binding proteins (Fig. 3a and Supplementary Table 3). Subsequently, we overlapped our MS data with the DDX39B-interactome from Biogrid database and human E3 ubiquitin ligases from iUUCD 2.0 database,^26^ and identified TRIM28 as the most likely E3 ubiquitin ligase of DDX39B (Fig. 3b, c). Immunoprecipitation assays demonstrated that both exogenous and endogenous DDX39B were able to interact with TRIM28 in NSCLC cells (Fig. 3d–g). A GST pull-down assay showed a direct association between DDX39B and TRIM28 protein in vitro (Fig. 3h). To further explore the interaction between DDX39B and TRIM28 in viable NSCLC cells, we performed a bimolecular fluorescence complementation (BiFC) assay and observed reconstituted fluorophore signals in NSCLC cells following co-transfection with VN173-DDX39B and VC155-TRIM28 plasmid (Fig. 3i). Notably, the binding of DDX39B and TRIM28 was turned stronger in the presence of EGF or TGF-β stimulation as well as in A549-BM cells compared with control cells (Fig. 3j, k). To further map the key amino acids of DDX39B responsible for the TRIM28 interaction, we exploited the available crystal structures of DDX39B and TRIM28 to perform a protein‒protein docking prediction via the ZDOCK server (http://zdock.umassmed.edu/). The molecular docking model showed that the α-helix of TRIM28 was tightly embedded in the groove of DDX39B (Supplementary Fig. 4a). We calculated the energy decomposition of the DDX39B-TRIM28 complex (Supplementary Fig. 4b), and chose the top five amino acids of DDX39B that located at the binding interface and potentially involved in TRIM28 association for further investigation (Fig. 3l and Supplementary Table 4). GST pull-down assays demonstrated that the P322A mutant of DDX39B remarkably diminished the interplay between DDX39B and TRIM28 (Fig. 3m). These data imply that DDX39B is a novel substrate of TRIM28 and that the P322 residue of DDX39B is crucial for its binding with TRIM28.

### TRIM28 stabilizes DDX39B protein by triggering K63-linked polyubiquitination at K241, K384, and K398 residues

**Fig. 4:**
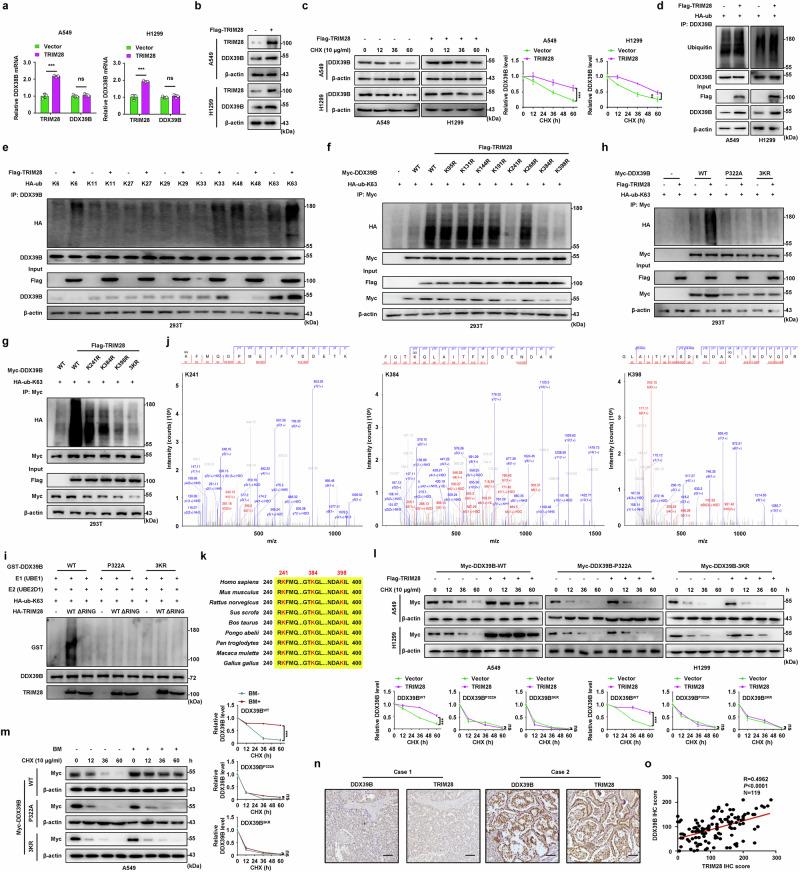
TRIM28 enhances the stabilization of DDX39B protein by inducing K63-linked ubiquitination at Lys241, Lys284, and Lys398 residues of DDX39B. NSCLC cells were transfected with Flag-TRIM28, and the (**a**) mRNA (*n* = 3) and (**b**) protein expression of DDX39B were determined by RT‒qPCR and Western blotting, respectively. **c** The expression of TRIM28 on DDX39B protein degradation was detected by CHX chase analysis (*n* = 3). **d** Cells were co-transfected with Flag-TRIM28 and HA-ub, and the effect of TRIM28 on DDX39B total ubiquitination was examined by immunoprecipitation assay. **e** 293T cells were co-transfected with the indicated ubiquitin plasmids and Flag-TRIM28, and DDX39B ubiquitination were analyzed by immunoprecipitation assay. **f** 293T cells were co-transfected with Flag-TRIM28, HA-ub-K63 and Myc-DDX39B wild-type (WT) or indicated lysine to arginine (K to R) mutants, and DDX39B ubiquitination were analyzed by immunoprecipitation assay. **g** 293T cells were co-transfected with Flag-TRIM28, HA-ub-K63 and Myc-DDX39B WT or indicated lysine to arginine (K to R) mutants (K241R, K384R, K398R or 3KR (K241R, K384R and K398R)), and DDX39B ubiquitination were analyzed by immunoprecipitation assay. **h** 293T cells were co-transfected with Flag-TRIM28, HA-ub-K63 and Myc-DDX39B WT or indicated mutants (P322A, 3KR), and DDX39B ubiquitination were analyzed by immunoprecipitation assay. **i** The impact of TRIM28 WT or ΔRING (RING domain deletion) on the K63-linked ubiquitination of purified DDX39B WT, P322A or 3KR was examined by in vitro ubiquitination assay. **j** Mass spectrometry analysis reveals the K63-linked ubiquitination sites of DDX39B at K241, K384, K398 residues. **k** Sequence alignment around K241, K384, K398 residues of DDX39B in different species was shown. **l**, **m** Indicated NSCLC cells were transfected with DDX39B WT, P322A or 3KR mutants, and then treated with CHX (10 μg/ml) for the indicated times (0, 12, 36 and 60 h). The ratio of DDX39B to β-actin was calculated. The line graph represents the rate of DDX39B degradation (*n* = 3). **n**, **o** IHC assay was performed to examine the relationship between DDX39B and TRIM28 expression in primary NSCLC tissues (*n* = 119). **n** Representative images of DDX39B and TRIM28 expression were shown. **o** The correlation between DDX39B and TRIM28 expression was evaluated. Scale bars, 50 µm. Graphs represent data as the mean ± s.d. *P* values were determined by one-way ANOVA (**a**), two-way ANOVA (**c**, **l**, **m**) or Pearson correlation coefficient analysis (**o**). ns, not significant, **P < 0.01, ***P < 0.001

To explore whether TRIM28 enabled to induce ubiquitination of DDX39B, we first examined the effect of TRIM28 on DDX39B expression. Neither TRIM28 overexpression nor TRIM28 silencing altered the mRNA expression of DDX39B gene in A549 and H1299 cells (Fig. 4a and Supplementary Fig. 5a). However, exogenous introduction of TRIM28 augmented the protein expression of DDX39B, while TRIM28 deficiency significantly reduced DDX39B protein levels in NSCLC cells (Fig. 4b and Supplementary Fig. 5b). CHX assay revealed that TRIM28 overexpression prolonged the half-life of DDX39B protein (Fig. 4c). Conversely, TRIM28 insufficiency accelerated the degradation of DDX39B protein (Supplementary Fig. 5c). Next, we investigated whether the ubiquitin-proteasome system or lysosomal pathway was involved in TRIM28-mediated DDX39B stability. Administration of proteasome inhibitor MG132 effectively abrogated the diminished DDX39B protein level caused by TRIM28 knockdown, whereas lysosome inhibitor chloroquine exhibited no influence on TRIM28-mediated DDX39B protein expression (Supplementary Fig. 5d). Interestingly, TRIM28 upregulation slightly enhanced the total ubiquitination of DDX39B protein (Fig. 4d). Ubiquitin molecule contains 7 lysine residues (including K6, K11, K27, K29, K33, K48 and K63) that can be linked to form poly-ubiquitin chain.^27^ To further characterized the linkage type of DDX39B ubiquitination modulated by TRIM28, 293T cells were co-transfected with Flag-TRIM28 and the indicated ubiquitin plasmids. As shown in Fig. 4e, overexpression of TRIM28 predominantly increased the K63-linked ubiquitination of DDX39B, while slightly augmented the K6- or K33-linked ubiquitination of DDX39B. Conversely, decreased K48-linked ubiquitination of DDX39B was observed in the presence of TRIM28 (Fig. 4e). Accumulating evidence has reported that K63-linked polyubiquitination promotes target protein activation, whereas K48-linked polyubiquitination facilitates the degradation of substrate proteins.^28,29^ Thus, we focused on K63- and K48-linked polyubiquitination for further investigation. TRIM28 directly potentiated K63-linked polyubiquitination of DDX39B, but failed to induce K48-linked polyubiquitination of DDX39B according to in vitro ubiquitination assays (Supplementary Fig. 5e, f). Notably, the RING domain deletion (ΔRING) and enzymatic-dead C65A mutant of TRIM28 was unable to stimulate K63-linked polyubiquitination of DDX39B (Supplementary Fig. 5e, f). These results suggest that TRIM28 directly interacts with DDX39B and augments K63-linked polyubiquitination of DDX39B, leading to the stabilization and elevation of DDX39B protein in NSCLC cells. According to IP-MS analysis, we identified top 8 lysine residues (K95, K131, K144, K191, K241, K268, K384 and K398) as potential ubiquitination sites of DDX39B (Supplementary Table 5). Next, we mutated lysine residues to arginine and evaluated the effect of these mutations on TRIM28-mediated DDX39B ubiquitination. K241R, K384R and K398R mutation of DDX39B attenuated DDX39B ubiquitination in the presence of TRIM28, and the 3KR (K241R/K384 R/K398 R) mutation substantially abolished DDX39B ubiquitination (Fig. 4f, g). Consistently, TRIM28 strengthened DDX39B protein levels, which was prevented by K241R, K384R, K398R and 3KR mutants (Supplementary Fig. 5g). As expected, both P322A and 3KR mutant of DDX39B remarkably diminished the K63-linked polyubiquitination of DDX39B triggered by TRIM28 (Fig. 4h). A resistant result was confirmed by in vitro ubiquitination assay (Fig. 4i and Supplementary Fig. 5h). Moreover, TRIM28 lacking RING domain or enzyme activity failed to catalyze the K63-linked polyubiquitination of DDX39B in vitro (Fig. 4i and Supplementary Fig. 5h). The mass spectrometry identification of K241, K384 and K398 ubiquitination of DDX39B was present in Fig. 4j. We analyzed the amino acid sequence of DDX39B and found that K241, K384 and K398 residues were evolutionarily conserved (Fig. 4k). Additionally, enforced expression of TRIM28 delayed the degradation of wild-type (WT) DDX39B, but had no obvious impact on the P322A and 3KR mutant (Fig. 4l). Similar results were obtained in EGF- or TGF-β-induced EMT-metastatic models and A549-BM cells (Fig. 4m and Supplementary Fig. 5i, j). We also found a positive correlation between DDX39B and TRIM28 protein expression in NSCLC clinical samples (Fig. 4n, o). Moreover, worse prognosis was observed in high TRIM28-expressing group compared with low TRIM28-expressing group (Supplementary Fig. 5k). Patients with DDX39B_high_/TRIM28_high_ level exhibited shortest overall survival than patients in the other groups, whereas patients with DDX39B_low_/TRIM28_low_ expression had the best outcome (Supplementary Fig. 5l). These findings indicate that TRIM28 augments DDX39B protein expression via induction of K63-linked polyubiquitination at K241, K384, and K398 residues.

### DDX39B promotes EMT reprogramming and metastasis in NSCLC cells

**Fig. 5:**
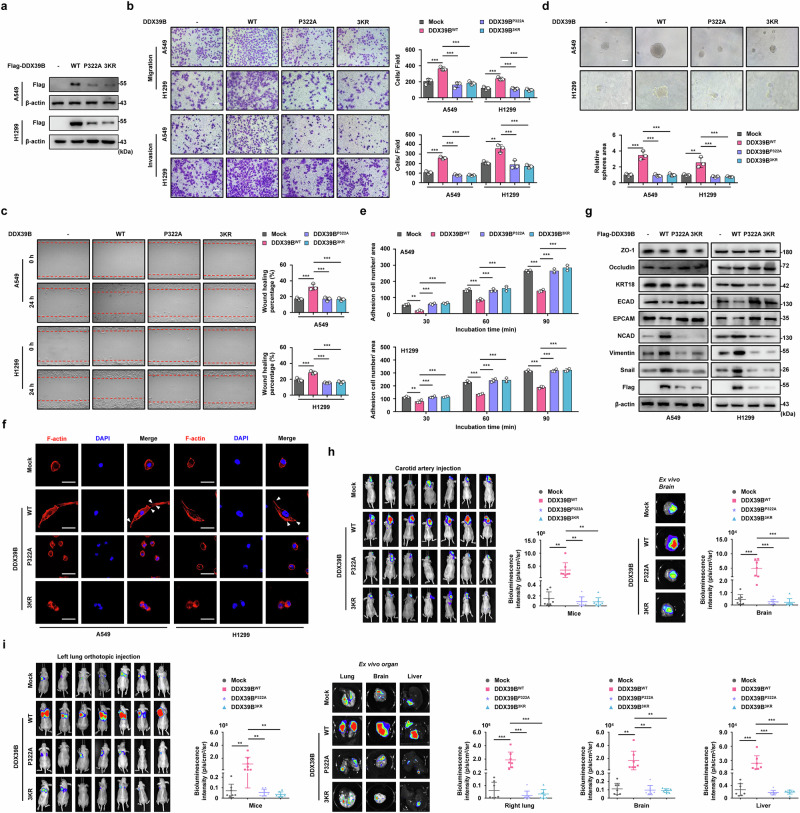
DDX39B promotes EMT reprogramming and metastasis in NSCLC cells. **a** Reconstituted expression of the wild-type (WT), P322A and 3KR (K241R, K384R and K398R) of Flag-DDX39B in NSCLC cell lines (A549 and H1299) was verified by Western blotting. The effects of DDX39B WT, P322A and 3KR mutant on (**b**) cell migration and invasion, (**c**) wound closure, (**d**) 3D sphere formation and (**e**) adhesion in NSCLC cell lines were analyzed (*n* = 3). **f** F-actin and nuclei were stained using rhodamine-phalloidin and DAPI, respectively. Representative images were shown, and the white triangles represented cilia and pseudopodia. **g** Protein expression of mesenchymal markers (NCAD, Vimentin and Snail) and epithelial markers (ZO-1, Occludin, KRT18, ECAD and EPCAM) in indicated NSCLC cells were measured by Western blotting. **h** Indicated A549 cells were injected into carotid artery. Six weeks later, the representative bioluminescence images and intensities (p/s/cm2/sr) of mice and isolated brain were presented and quantified (*n* = 7/group). **i** Indicated A549 cells were orthotopically injected into the left pulmonary tissue. Eight weeks later, the representative bioluminescence images and intensities (p/s/cm2/sr) of mice and isolated organs (including the right lung, brain, and liver) were shown and quantified (*n* = 7/group). Scale bars, 50 µm. Graphs represent data as the mean ± s.d. *P* values were determined by one-way ANOVA (**b–e**, **h**, **i**). **P < 0.01, ***P < 0.001

To determine the function of TRIM28-mediated K63-linked polyubiquitination of DDX39B in NSCLC development, we established NSCLC cells stably expressing DDX39B^WT^, DDX39B^P322A^ or DDX39B^3KR^. Overexpression of wild-type DDX39B, but not P322A and 3KR mutants, resulted in increased migration, invasion, and stem cell-like characteristics as well as reduced adhesion in A549 and H1299 cells, as confirmed by Transwell, wound closure, 3D sphere formation and cell adhesion assays (Fig. 5a–e). In contrast, DDX39B-deficient NSCLC cells had lower motility (Supplementary Fig. 6a–e). F-actin stress fiber staining showed that NSCLC cells with upregulated DDX39B expression exhibited a spindle-like morphology, whereas DDX39B P322A or 3KR mutant eliminated F-actin rearrangement compared with DDX39B WT (Fig. 5f). Moreover, a more flat or round morphology was observed in DDX39B-insufficient NSCLC cells than control cells (Supplementary Fig. 6f). Ectopic expression of DDX39B significantly increased the expression of mesenchymal markers (N-cadherin, Vimentin and Snail) and diminished the expression of epithelial markers (ECAD and EPCAM), whereas DDX39B silencing showed the opposite phenomenon (Fig. 5g and Supplementary Fig. 6g). Notably, neither P322A nor 3KR mutant had an obvious effect on the expression of the above EMT-related markers (Fig. 5g). To further investigate whether DDX39B was implicated in the malignant development of NSCLC in vivo, we first exploited a BM model via carotid artery injection. As shown in Fig. 5h, exogenous introduction of wild-type DDX39B accelerated the metastatic colonization of A549 cells in the brain compared with mock group and DDX39B P322A or 3KR mutant group. Conversely, DDX39B-insufficient group displayed less brain-metastatic lesions than negative control group (Supplementary Fig. 6h). Orthotopic lung cancer model also showed enhanced spontaneous contralateral intrapulmonary metastasis and distal organ metastasis (including liver and brain) in A549 cells overexpressing wild-type DDX39B, rather than mock, DDX39B P322A or 3KR mutant group (Fig. 5i). DDX39B deficiency greatly delayed the metastatic potential of A549 cells in vivo (Supplementary Fig. 6i). These data indicate that prevention of DDX39B-TRIM28 interplay or TRIM28-mediated DDX39B ubiquitination effectively restricts the potent pro-EMT and pro-metastatic role of DDX39B in NSCLC.

### DDX39B interacts with ECAD and recruits the Src/Hakai complex to promote ECAD ubiquitination and subsequent lysosomal degradation

**Fig. 6:**
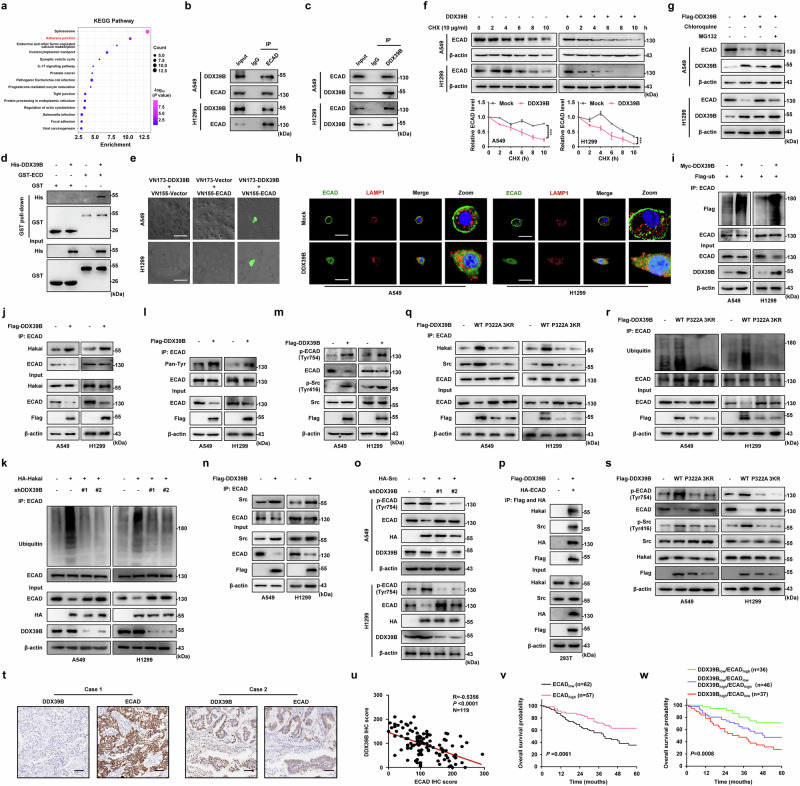
DDX39B interacts with and accelerates ECAD ubiquitination and lysosomal degradation by recruiting Src and Hakai. **a** KEGG enrichment analysis of the DDX39B interactome. **b**, **c**The endogenous association between DDX39B and ECAD was determined by immunoprecipitation analysis. **d** GST pull down assay of purified GST-ECD (GST-tagged ECAD cytoplasmic domain) and His-DDX39B protein. **e** Indicated BiFC plasmids were transfected into NSCLC cells, and in vivo interaction between DDX39B and ECAD was detected by BiFC analysis. **f** The effect of DDX39B on ECAD protein stability was detected by CHX analysis (*n* = 3). **g** DDX39B-overexpressed NSCLC cells were treated with chloroquine (50 μM, 8 h) or MG132 (10 μM, 8 h), and ECAD expression was detected by Western blot analysis. **h** The colocalization of ECAD and LAMP1 (a lysosomal marker) in DDX39B-overexpressed cells was determined by immunofluorescence staining. **i** Cells were co-transfected with Myc-DDX39B and Flag-ub, and the effect of DDX39B on ECAD ubiquitination was examined by immunoprecipitation assay. **j** The effect of DDX39B on the interaction between ECAD and Hakai was determined by immunoprecipitation analysis. **k** DDX39B-deficient NSCLC cells were transfected with or without HA-Hakai plasmid. The expression and ubiquitination of ECAD protein were detected. **l** The pan-pTyr and (**m**) Tyr754-phosphorylation of ECAD were examined in DDX39B-overexpressed cells. **n** The effect of DDX39B on the interaction of ECAD with Src was evaluated by immunoprecipitation assay. **o** DDX39B-silenced cells were transfected with or without HA-Src plasmid, and pECAD^Y754^ was analyzed by Western blot analysis. **p** 293T cells were co-transfected with Flag-DDX39B and HA-ECAD plasmids, and the lysates were processed for tandem affinity purification by using anti-Flag and anti-HA magnetic beads. The effects of DDX39B WT, P322A or 3KR (K241R, K384R and K398R) mutant on (**q**) the interaction between ECAD and Hakai or Src, (**r**) the expression of ECAD and ECAD ubiquitination, and (**s**) the expression of pECAD^Y754^. **t, u** IHC assay was performed to examine the relationship between DDX39B and ECAD expression in primary NSCLC tissues (*n* = 119). **t** Representative images of DDX39B and ECAD expression were shown. **u** The correlation between DDX39B and ECAD expression was evaluated. **v** Kaplan‒Meier estimates of overall survival probability based on ECAD protein expression in NSCLC patients (*n* = 119). **w** Prognostic value of DDX39B combined with ECAD expression in NSCLC patients (*n* = 119). Scale bars, 50 µm. Graphs represent data as the mean ± s.d. *P* values were determined by two-way ANOVA (**f**). Pearson correlation coefficient analysis (**u**), and log-rank test (**v, w**). ***P < 0.001

To further explore the potential molecular mechanism by which DDX39B mediated EMT and metastasis in NSCLC cells, we analyzed DDX39B interactome as described above (Supplementary Table 3). KEGG enrichment analysis showed that the adherens junction (AJ) pathway, which comprises ECAD, ACTN1, LMO7, ACTN4, and IQGAP1, was highly enriched (Fig. 6a). Considering that AJ pathway was the foundation for EMT activation,^30,31^ we first confirmed the interplay between DDX39B and these proteins (Supplementary Fig. 7a). Because ECAD has been widely demonstrated to be a key factor contributing to EMT and metastasis, we focused on ECAD for further investigation. Co-immunoprecipitation assay showed an endogenous binding of DDX39B and ECAD (Fig. 6b, c). In addition, we found a direct association between purified recombinant His-DDX39B and GST-ECD (ECAD cytoplasmic domain) proteins (Fig. 6d). We also observed visible fluorophores in living NSCLC cells co-transfected with VN173-DDX39B and VC155-ECAD plasmid (Fig. 6e). DDX39B upregulation significantly accelerated ECAD protein degradation, whereas this effect was delayed by DDX39B silencing (Fig. 6f and Supplementary Fig. 7b). Notably, treatment with chloroquine largely rescued the decline of ECAD protein in DDX39B-overexpressed cells, while treatment with MG132 had limited impact (Fig. 6g). These results suggested that DDX39B promoted ECAD protein degradation mainly via lysosome pathways. Consistently, DDX39B enhanced the lysosomal distribution of ECAD by colocalizing with lysosome marker LAMP1 (Fig. 6h). A similar result was obtained by fractionation assay (Supplementary Fig. 7c, d). Furthermore, overexpression of DDX39B considerably increased ECAD ubiquitination, while interference with DDX39B had the opposite effect (Fig. 6i and Supplementary Fig. 7e).

Since the E3 ubiquitin ligase Hakai has been reported to control the ubiquitination and lysosomal degradation of ECAD,^32^ we next examined the effect of DDX39B on Hakai expression and Hakai-ECAD interplay. Our results showed that DDX39B overexpression did not alter Hakai expression but strengthened the association of ECAD with Hakai (Fig. 6j). Moreover, the effect of DDX39B on ECAD ubiquitination and degradation was largely interrupted by Hakai suppression (Supplementary Fig. 7f). Strikingly, DDX39B deficiency remarkably blocked Hakai-triggered ubiquitination and degradation of ECAD (Fig. 6k), suggesting an essential function of DDX39B during this process. Previous studies have demonstrated that Src-derived ECAD^Tyr754^ phosphorylation was the precondition of Hakai-mediated ECAD ubiquitination.^33,34^ We next assessed whether DDX39B was responsible for the phosphorylation of ECAD^Tyr754^. Ectopic expression of DDX39B augmented the pan-Tyr and Tyr754-phosphorylation of ECAD (Fig. 6l, m). In contrast, DDX39B depletion decreased pECAD^Y754^ in NSCLC cells (Supplementary Fig. 7g). Interestingly, DDX39B facilitated the interaction between Src and ECAD (Fig. 6n). Conversely, the inhibition of Src resulted in reduced Tyr754-phosphorylation and ubiquitination of ECAD in the presence of DDX39B (Supplementary Fig. 7h). More importantly, Src-mediate increase of pECAD^Y754^ was abolished by knockdown of DDX39B (Fig. 6o). Given the indispensable role of DDX39B in Src-induced ECAD phosphorylation and subsequent ubiquitination caused by Hakai, we inferred that DDX39B may recruit Src and Hakai to degrade ECAD. To test this hypothesis, we performed a tandem affinity purification analysis and our results revealed that DDX39B formed a complex with ECAD, Src and Hakai (Fig. 6p). Compared with wild-type DDX39B, DDX39B P322A or 3KR mutant was unable to recruit Src and Hakai to interact with ECAD and diminished Tyr754-phosphorylation and ubiquitination of ECAD (Fig. 6q–s). Loss of the ATPase and RNA helicase activity of DDX39B caused by E197A or SAT/AAA mutant^35^ also triggered the phosphorylation and ubiquitination of ECAD, accompanied by decreased ECAD protein level in NSCLC cells, suggesting that DDX39B-induced ECAD degradation was independent of its unwinding activity (Supplementary Fig. 8a, b). Consistent with the cellular experiments, we found an inverse correlation between DDX39B and ECAD protein levels in NSCLC tissue (Fig. 6t, u). Overall survival was longer in the high ECAD-expressing group than in the low ECAD-expressing group (Fig. 6v). Patients with DDX39B_high_/ECAD_low_ expression exhibited shorter overall survival than patients in the other groups, while patients with DDX39B_low_/ECAD_high_ expression had the best outcome (Fig. 6w). Altogether, these data indicate that DDX39B directly binds to ECAD and recruits Src and Hakai to promote ECAD phosphorylation and subsequent ubiquitination-lysosomal degradation.

### DDX39B promotes disassociation of the ECAD/β-catenin complex and activates β-catenin signaling

Accumulating evidence has demonstrated that β-catenin associates with ECAD to modulate metastasis in various types of cancers.^36^ Because DDX39B bound to and accelerated ECAD degradation, we next explored the impact of DDX39B on ECAD/β-catenin complex. Co-immunoprecipitation assay showed that DDX39B upregulation impaired the stability of the ECAD/β-catenin complex (Supplementary Fig. 9a). Exogenous introduction of DDX39B resulted in the translocation of β-catenin from the cytoplasm to the nucleus, whereas DDX39B knockdown restricted the EGF-induced nuclear accumulation of β-catenin (Supplementary Fig. 9b–d). TOP/FOP flash assay revealed that the transcriptional activity of β-catenin was markedly increased in DDX39B-overexpressed cells, while loss of DDX39B caused the opposite result (Supplementary Fig. 9e, f). Similar results were obtained from the expression of β-catenin downstream target genes (including c-Myc, Cyclin D1, MMP2, MMP7 and MMP9) (Supplementary Fig. 9g, h). Strikingly, DDX39B P322A or 3KR mutant failed to dissociate the ECAD/β-catenin complex or to enhance the nuclear accumulation of β-catenin (Supplementary Fig. 9i, j). Moreover, decreased transcriptional activity of β-catenin and the transcription of β-catenin target genes were observed in NSCLC cells expressing P322A or 3KR mutant than in those expressing wild-type DDX39B (Supplementary Fig. 9k, l). Altogether, these findings indicate that DDX39B facilitates the nuclear localization of β-catenin via disassociation of the ECAD/β-catenin complex and augments the transcription of β-catenin target genes, which further promotes DDX39B-triggered aggressive phenotype of NSCLC cells.

### The overexpression of ECAD was sufficient to abate DDX39B-mediated EMT reprogramming and metastasis in NSCLC

**Fig. 7:**
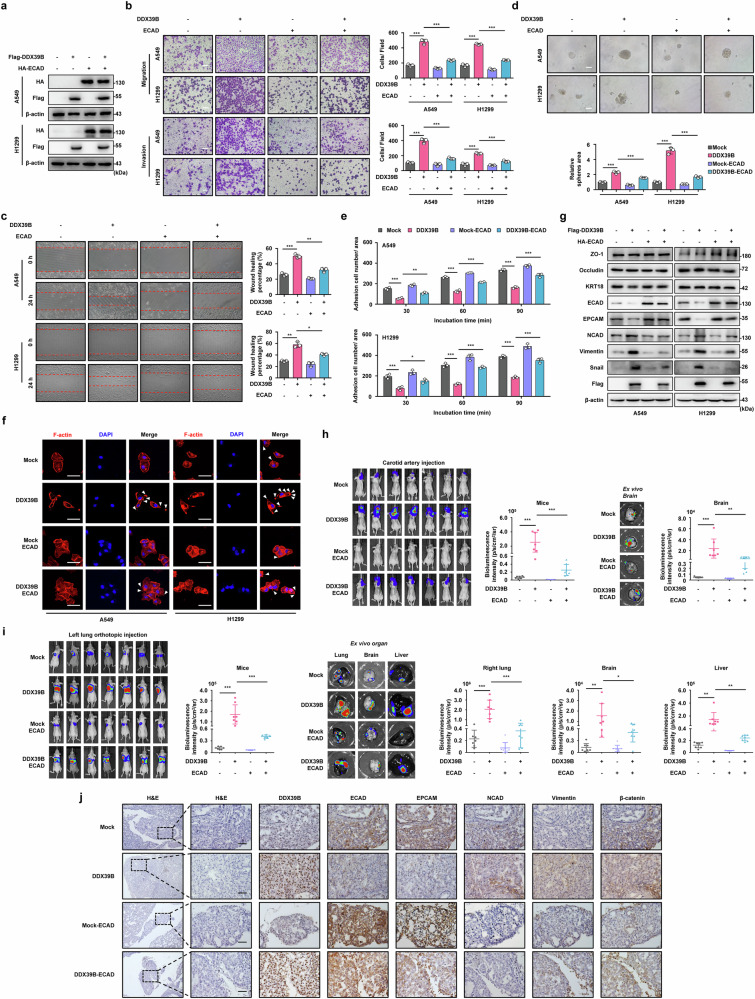
The overexpression of ECAD is sufficient to abolish DDX39B-mediated EMT reprogramming and metastasis in NSCLC in vitro and in vivo. **a** Reintroduced ECAD expression was performed in DDX39B-overexpressed NSCLC cell lines (A549 and H1299) and verified by Western blotting. **b** Cell migration and invasion, (**c**) wound closure, (**d**) sphere formation and (**e**) adhesion in indicated NSCLC cell lines were determined (*n* = 3). **f** F-actin and nuclei were stained using rhodamine-phalloidin and DAPI, respectively. Representative images were shown, and the white triangles represented cilia and pseudopodia. **g** The expression of EMT markers was detected by Western blot analysis. **h** Indicated A549 cells were injected into carotid artery. Six weeks later, the representative bioluminescence images and intensities (p/s/cm2/sr) of mice and isolated brain were presented and quantified (*n* = 7/group). **i** Indicated A549 cells were orthotopically injected into the left pulmonary tissue. Eight weeks later, the representative bioluminescence images and intensities (p/s/cm2/sr) of mice and isolated organs (including the right lung, brain, and liver) were shown and quantified (*n* = 7/group). **j** Immunohistochemistry analysis was performed to examine the relationship between DDX39B expression and EMT markers (ECAD, EPCAM, NCAD, Vimentin, β-catenin) in indicated orthotopic lung cancer tissues. Representative images were shown. Scale bars, 50 µm. Graphs represent data as the mean ± s.d. *P* values were determined by one-way ANOVA (**b**–**e**, **h**, **i**). *P < 0.05, **P < 0.01, ***P < 0.001

To elucidate whether ECAD contributes to the pro-EMT and pro-metastatic function of DDX39B, we reintroduced ECAD expression into DDX39B-overexpressed A549 and H1299 cells. Upregulated ECAD considerably impaired DDX39B-promoted migration, invasion and self-renewal ability of NSCLC cells (Fig. 7a–d). Moreover, the reduced adhesive ability of NSCLC cells caused by DDX39B was partially restored by ECAD overexpression (Fig. 7e). Consistently, DDX39B-induced EMT-like characteristics were remarkably abrogated by reintroduction of ECAD (Fig. 7f, g). In agreement with our in vitro findings, the DDX39B-overexpressed groups displayed more metastatic colonization in the brain than control group, which was remarkably reversed by enforced expression of ECAD (Fig. 7h). Notably, NSCLC cells expressing DDX39B together with ECAD displayed fewer metastatic foci in various distal organs than cells expressing DDX39B alone (Fig. 7i). Additionally, orthotopic tumors with DDX39B overexpression exhibited elevated staining of mesenchymal markers NCAD, Vimentin and β-catenin as well as diminished level of epithelial marker EPCAM, which were reversed by ECAD reintroduction (Fig. 7j). Thus, our findings reveal that the suppression of ECAD is critical for DDX39B-mediated malignant progression in NSCLC.

### Artesunate inhibits DDX39B protein stability and tumor metastasis by blocking the DDX39B-TRIM28 interplay in NSCLC

**Fig. 8:**
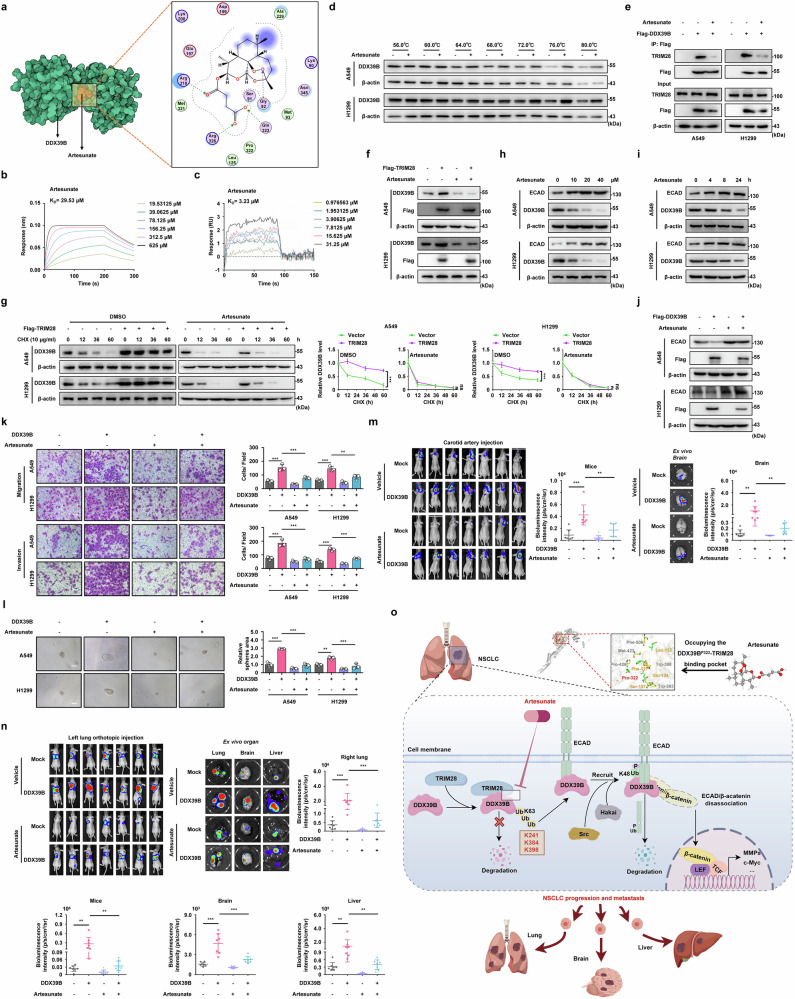
Artesunate inhibits DDX39B protein stability and tumor metastasis by blocking the DDX39B-TRIM28 interplay in NSCLC. **a** The overall structure of the complex and two-dimensional ligand-interaction maps of the binding of artesunate to DDX39B were revealed by a computational model. DDX39B (green), artesunate (orange) (left). The interacting amino acids of DDX39B to artesunate were shown (right). **b** The binding affinity of artesunate to DDX39B was determined by bio-layer interferometry (BLI) assay. **c** The equilibrium dissociation constant (K_D_) of artesunate interacting with DDX39B was calculated using surface plasmon resonance (SPR) assay. **d** Cellular thermal shift assay (CETSA) was performed in NSCLC cell lines treated with DMSO or 100 μM artesunate. A loading control was established using β-actin. **e** The indicated cells were treated with or without 20 µM artesunate for 24 h. The interaction between DDX39B and TRIM28 was analyzed by immunoprecipitation assay. **f** NSCLC cells with or without stable expression of TRIM28 were treated with artesunate (20 μM, 24 h), and the effect of artesunate on DDX39B expression was analyzed. **g** TRIM28-overexpressing NSCLC cells were co-treated with or without artesunate (20 μM) and CHX (10 μg/ml) for the indicated times (0, 12, 36 and 60 h). The ratio of DDX39B to β-actin was calculated. The line graph represents the rate of DDX39B degradation (*n* = 3). NSCLC cells were treated with artesunate at the indicated (**h**) doses and (**i**) times, and the expression of DDX39B and ECAD was detected by Western blotting. **j** NSCLC cells with or without stable expression of DDX39B were treated with artesunate (20 μM, 24 h), and the effect of artesunate on DDX39B-induced ECAD degradation was analyzed. The indicated NSCLC cells were treated with or without artesunate (20 μM) (*n* = 3). The abilities of (**k**) cell migration, invasion and (**l**) sphere formation were investigated. **m** Indicated A549 cells were injected into carotid artery. Two weeks later, mice were intraperitoneally injected with artesunate (100 mg/kg per two day and per mice) for four weeks. Representative bioluminescence images and intensities (p/s/cm2/sr) of mice and isolated brain were presented and quantified (*n* = 7/group). **n** Indicated A549 cells were orthotopically inoculated into the left pulmonary tissue. Three weeks later, the mice were intraperitoneally injected with artesunate (100 mg/kg per two day and per mice) for five weeks, the representative bioluminescence images and intensities (p/s/cm2/sr) of mice and isolated organs (including the right lung, brain, and liver) were shown and quantified (*n* = 7/group). **o** Schematic illustration of TRIM28/DDX39B/ECAD axis-mediated metastasis in NSCLC (by Figdraw). Scale bars, 50 µm. Graphs represent data as the mean ± s.d. *P* values were determined by two-way ANOVA (**g**) or one-way ANOVA (**k**–**n**). **P < 0.01, ***P < 0.001

Given the essential role of the DDX39B-TRIM28 interaction in TRIM28-mediated K63-linked polyubiquitination and the subsequent upregulation of DDX39B, we hypothesized that targeting this interaction could serve as a promising therapeutic strategy for NSCLC. Therefore, we performed virtual drug screening on the basis of the critical P322 residue of DDX39B on the DDX39B-TRIM28 binding interface. This screening identified a potential candidate, artesunate, an FDA-approved drug widely used to treat choice globally in adults hospitalized with severe malaria.^37^ Molecular docking data of DDX39B-artesunate revealed that artesunate occupies the groove region where DDX39B interacts with TRIM28 (Fig. 8a). To corroborate the virtual screening findings, we subsequently employed a biolayer interferometry (BLI) assay to evaluate the binding affinity between DDX39B and artesunate. The binding kinetics curves indicated the equilibrium dissociation constant (K_D_) for artesunate binding to DDX39B was 29.53 μM (Fig. 8b). Furthermore, surface plasmon resonance (SPR) technology confirmed a comparable K_D_ of 3.23 μM for the artesunate-DDX39B interaction (Fig. 8c). Moreover, the thermal stabilization of the DDX39B protein markedly increased upon artesunate treatment compared with that in the control group by using a cellular thermal shift assay (CETSA) (Fig. 8d). These results indicated a direct interaction between artesunate and DDX39B protein. Notably, artesunate substantially disrupted the DDX39B-TRIM28 complex (Fig. 8e) and abolished the TRIM28-induced upregulation of DDX39B protein expression (Fig. 8f). CHX assay showed that the extension of the DDX39B protein half-life induced by TRIM28 was negated by artesunate treatment (Fig. 8g). In addition, artesunate reduced the protein level of DDX39B, which was accompanied by an increase in ECAD expression, in a dose- and time-dependent manner (Fig. 8h, i). We also found that artesunate effectively reversed the effect of DDX39B on ECAD degradation (Fig. 8j). Moreover, artesunate significantly attenuated the enhanced migration, invasion and stem cell-like characteristics of NSCLC cells induced by DDX39B (Fig. 8k, l). Similar results were verified by F-actin stress fiber staining and assessment of EMT-related markers (Supplementary Fig. 10a, b). In comparison to the untreated group, cells overexpressing DDX39B exhibited reduced brain-metastatic potential following exposure to artesunate in a carotid artery injection-induced BM model (Fig. 8m). Consistent results were supported by orthotopic lung cancer models, wherein artesunate largely impaired the pro-metastatic effects of DDX39B on spontaneous contralateral intrapulmonary metastasis and distal organ metastasis (including liver and brain) in NSCLC cells (Fig. 8n). Collectively, these data reveal that artesunate directly interacts with DDX39B to disrupt the DDX39B-TRIM28 binding, which subsequently leads to the degradation of DDX39B protein and inhibition of its pro-metastatic function in NSCLC cells.

## Discussion

DDX39B was frequently upregulated in various types of cancers. This was partly attributable to transcriptional activation, because our previous study demonstrated that Sp1 directly bound to the DDX39B promoter and induced its transcription in colorectal cancer (CRC) cells.^38^ Here, we reported that the protein expression of DDX39B, but not its mRNA expression, was notably increased in brain metastatic tissues of NSCLC patients compared with patient-matched primary tumors, suggesting a post-translational regulation of DDX39B during metastasis process. We identified DDX39B as a novel and specific substrate of the E3 ubiquitin ligase TRIM28. Our data verified that TRIM28 promoted the K63-linked ubiquitination of DDX39B at K241, K384, and K398 residues, accompanied by the reduced K48-linked ubiquitination of DDX39B, leading to the increased stability and expression of DDX39B protein. A previous study demonstrated that Plk1 phosphorylates DDX39B and subsequently destabilizes DDX39B protein in a proteasome-dependent manner.^39^ Nevertheless, the authors did not identify the specific E3 ubiquitin ligase responsible for the degradation of DDX39B. Based on the aforementioned findings, we inferred that TRIM28 may compete with other E3 ubiquitin ligases for DDX39B binding to impede its latter-mediated K48-linked ubiquitination and degradation. In agreement with our observations, recent studies have reported similar models of two different E3 ligases on the competitive regulation of the same substrate ubiquitination in other proteins.^40,41^ According to the protein‒protein docking model, a variety of hydrophobic amino acids (including proline, phenylalanine, tryptophan and methionine) were enriched in the binding interface of the DDX39B-TRIM28 complex. Our previous study revealed that DDX39B R319 residue around P322 residue is indispensable for PKM2 binding and for DDX39B-triggered PKM2 nuclear accumulation and proliferation in CRC cells.^38^ These results suggest that this hydrophobic pocket within the helicase C-terminal domain of DDX39B may facilitate the stable interactions between DDX39B and other proteins. Consistently, the helicase C-terminal domain of DDX39B mediated its interaction with ALYREF to form the human TREX complex.^42^ Therefore, targeting the hydrophobic region of DDX39B protein may sufficiently and effectively eliminate DDX39B function. As expected, DDX39B P322A mutant (blockage of DDX39B-TRIM28 complex formation) or 3KR mutant (interruption of TRIM28-induced DDX39B ubiquitination) restricted the pro-metastatic potential of DDX39B in NSCLC cells. Our findings supported an oncogenic role of the TRIM28/DDX39B axis in the aggressive development of NSCLC, and DDX39B may be an attractive therapeutic target for NSCLC treatment.

ECAD protein encoded by the CDH1 gene was a well-known epithelial marker and was able to maintain cell polarity and tissue homeostasis. Downregulation of the CDH1 gene caused by germline mutations, DNA methylation and transcriptional repression has been described during the progression of multiple cancers.^43–45^ Several EMT transcription factors, including Snail, Slug, Twist and ZEB1/2, have been reported to inhibit CDH1 transcription by directly binding to the E-box motifs within the promoter region of the CDH1 gene.^46^ However, these studies mainly focused on the transcriptional regulation of the CDH1 gene. Here, we provided evidence that DDX39B directly interacted with ECAD and strengthened the physiological association of ECAD with Hakai and Src, which was independent of its RNA helicase activity. Importantly, loss of DDX39B prevented Src-mediated phosphorylation and Hakai-induced ubiquitination of ECAD. Based on the above observations, we proposed that DDX39B may act as an essential scaffold protein to recruit Src and Hakai to subsequently coordinate ECAD degradation in the lysosome. Our findings expand the understanding of the non-canonical RNA regulatory function of DDX39B. Moreover, we found a weaker blocking effect of the proteasome inhibitor MG132 than of the lysosome inhibitor chloroquine on the downregulation of ECAD caused by DDX39B, suggesting that DDX39B may encourage ECAD degradation via the ubiquitin–proteasome pathway. In agreement with our results, a recent study has confirmed that oxidative stress enhances RNF25-mediated ubiquitination and degradation of ECAD in HCC cells, which is greatly abrogated by MG132 administration.^47^ Whether DDX39B is involved in this process needs to be clarified in the future.

Although artesunate was an FDA-approved drug used worldwide for the clinical treatment of malaria, recent studies have revealed anticancer activity of artesunate against different cancer types via various mechanisms, such as induction of cell death, anti-oxidative stress, anti-inflammation, and inhibition of angiogenesis.^48,49^ A recent clinical trial showed that artesunate was well tolerated without liver toxicity and glioblastoma patients with artesunate treatment in remission maintenance have a median survival of 46 months.^50^ Artesunate has been shown to mediate enhance the sensitivity to sorafenib through the exacerbation of the AFAP1L2-SRC-FUNDC1 axis-dependent mitophagy in HCC.^51^ Previous studies have reported that artesunate inhibit cell metastasis, invasion and malignant progression by inhibiting c-Myc signaling-mediated aerobic glycolysis or MMP expression in lung cancer.^52,53^ However, the direct target of artesunate is poorly understood. In the present study, we demonstrated that artesunate directly bound to the DDX39B protein and impaired the interplay between DDX39B and TRIM28, suggesting that artesunate competed with TRIM28 for DDX39B binding. As a result, artesunate considerably eliminated TRIM28-mediated K63-linked ubiquitination and subsequent upregulation of DDX39B protein, which further inhibited DDX39B-induced degradation of ECAD protein and metastasis in NSCLC cells. Because our current data provided a novel effect of artesunate on the regulation of protein-protein interactions, we inferred that artesunate may be modified as a linker to assist the formation of the DDX39B-artesunate-E3 ligase ternary complex, which facilitated the ubiquitination and subsequent degradation of DDX39B through the ubiquitin-proteasome system. This application of PROteolysis Targeting Chimeras technology enables to inhibit the growth and metastasis of cancer cells by eliminating all functions of DDX39B protein.

In summary, our findings highlight the clinical significance of DDX39B in predicting the aggressive progression and prognosis of patients with NSCLC. TRIM28 potently stabilizes DDX39B protein by triggering K63-linked ubiquitination at K241, K384, and K398 residue, resulting in the upregulation of DDX39B in NSCLC cells. Importantly, we reported a non-classical helicase function of DDX39B as a cytoplasmic protein regulator that enhances the degradation of ECAD protein through recruitment of Src and Hakai, which further activates β-catenin signaling. We verified that the antimalarial drug artesunate was able to directly bind to DDX39B and prevent the interaction of DDX39B-TRIM28 complex, leading to the impairment of the pro-EMT and pro-metastatic function of DDX39B (Fig. 8o). Taken together, our data describe a novel mechanism by which TRIM28/DDX39B/ECAD axis contributes to the EMT reprogramming and metastasis in NSCLC and targeting DDX39B by artesunate is an effective and promising therapeutic approach for the treatment of NSCLC.

## Materials and methods

### Study approval

This study complied with all relevant ethical regulations and was approved by the Ethics Committee on Biomedical Research, West China Hospital of Sichuan University (protocol no. 2020358). All animal experiments were conducted under the supervision and approval of the Animal Ethics and Welfare Committee of Sichuan University (protocol no. 2021087A).

### Animal experiments

All mice were maintained in specific pathogen-free animal care conditions, including autoclaved food, litter and water at Sichuan University. Mice were kept at room temperature (21–25 °C), 50–60% humidity and a 12 h light–12 h dark cycle. BALB/c nude male mice (GemPharmatech) (6 weeks, 18–20 g) were purchased and used. The carotid artery injection model was constructed as indicated.^25^ Briefly, the indicated A549 cells stably expressing luciferase (A549-luc) were injected into the carotid artery of mice (2.5 × 10^5^ cells per mouse, 100 μl in PBS) and inoculated for six weeks. To establish a left lung orthotopic lung cancer model, A549-luc cells were mixed with Matrigel (Corning, 356234) in equal proportions and then injected into the left pulmonary tissue of mice (6 × 10^5^ cells per mouse, 50 μl in PBS) and inoculated for eight weeks. For the bioluminescent imaging assay, mice were intraperitoneally injected with 150 mg/kg D-luciferin potassium (Meilunstar^®^, MB1834) per mouse. The bioluminescent images and quantitative bioluminescent intensities (p/s/cm2/sr) of mice, separated primary tumors and metastases among isolated organs were captured and analyzed by Spectrum Living Image 4.2 software (Caliper Life Sciences).

### Immunoprecipitation (IP) followed by mass spectrometry (MS)

The indicated cell lysates were harvested and lysed as described above.^54^ The magnetic beads were prewashed with TBS buffer (150 mM NaCl, 50 mM Tris-HCl (pH = 7.4)) 3 times before immunoprecipitation. Cell lysates were immunoprecipitated using the indicated antibodies or corresponding normal IgG for endogenous Co-IP, and anti-Flag Magnetic Beads (Sigma, M8823) for exogenous Co-IP overnight at 4 °C. The immunoprecipitates were then washed 5 times with TBS buffer, and 2× loading buffer was added and boiled for 10 min, followed by Western blotting. For mass spectrometry, the Flag-DDX39B immunoprecipitates were isolated by SDS‒PAGE, and visualization was performed by using Feto SDS‒PAGE staining buffer (Affinibody Life Science AG, 18.001.10) for 15 min at room temperature. The prospectively specific gel bands (The molecular weight was around 70 kDa and above) were excised from the gel and subjected to mass spectrometry, which were provided by the Beijing Bio-Tech Pack Technology Company Ltd. Proteins that contained at least 4 unique peptides and exhibited corresponding molecular weights were selected for subsequent analysis.

### Identification of ubiquitination sites by MS analysis

The ubiquitination sites of DDX39B were characterized by *Jingjie* PTM BioLab. Briefly, 293T cells were co-transfected with Flag-DDX39B and HA-ub for 48 h and then treated with MG132 (10 μM, 8 h). Cell lysates were prepared as indicated and subjected to immunoprecipitation by using anti-Flag Magnetic Beads (Sigma, M8823). After washing washed 5 times with TBS buffer, ubiquitinated-Flag-DDX39B protein was eluted from the beads by using Flag peptides (Sigma, F4799). The ubiquitinated Flag-DDX39B protein were then digested with trypsin (Promega, V5111) to generate peptides. The peptides underwent analysis using a NSI source followed by tandem mass spectrometry (MS/MS) on a Q Exactive™ Plus instrument (Thermo Fisher Scientific), which was interfaced online to UPLC (PTM BIO). An electrospray voltage of 2.0 kV was applied. The *m*/*z* scan range for the full scan was set between 350 and 1800, with intact peptides being detected in the Orbitrap at a resolution of 70,000. Peptides were subsequently selected for MS/MS by using NCE setting of 28, and the resulting fragments were collected in the Orbitrap at a resolution of 17,500. A data-dependent acquisition method was employed, alternating between one MS scan and 20 MS/MS scans, with a dynamic exclusion of 15.0 s. Automatic gain control (AGC) was set at 5 × 10^4^. The MS/MS data were analyzed by Proteome Discoverer 1.3 software (Thermo Fisher Scientific).

### Protein purification and GST/His pull-down assay

The *Escherichia coli BL21 (DE3)* strain was transformed with recombinant GST-tag or His-tag plasmids involved in the article and grown at 37 °C in lysogeny broth medium (supplemented with ampicillin (100 µg/ml) or kanamycin (100 µg/ml)). Protein expression was triggered by the addition of IPTG (500 µM) for 14–16 h at 18 °C when the optical density (OD) reached 0.6–0.8. Protein purification was carried out using GST agarose (Sangon Biotech, C600031) or Ni-NTA agarose (Invitrogen, R90101) in accordance with the protocol recommended by the manufacturer. Finally, the proteins were gel-filtered on HiLoad 60 Superdex 200 columns (GE Healthcare), verified by SDS‒PAGE and aliquoted for storage at *−*80 °C. To perform GST or His pull-down assays, GST or Ni-NTA agarose was prewashed with TBS buffer three times, and then the indicated purified protein was incubated with agarose at 4 °C overnight. The agarose was rinsed 5 times with TBS buffer, and the binding proteins were eluted by adding 1% SDS and analyzed by immunoblotting.

### Bio-Layer Interferometry (BLI) assay

The binding affinities of artesunate with His-DDX39B protein were determined by a BLI assay using Octet RED96 (ForteBio, Menlo Park, CA, USA). To obtain biotinylated His-DDX39B protein, EZ-Link NHS-PEG12-Biotin (Thermo Fisher, A35389) was added and subjected to the manufacturer’s recommended protocol. Subsequent assays were conducted at 35 °C in a total volume of 200 µl per well. Biotin-labeled His-DDX39B protein was loaded on super streptavidin (SSA) biosensors (Octet, 18-5057) in PBS buffer until equilibrium. The same sensors were incubated in PBS buffer without protein for background binding control. Artesunate was delivered at different concentrations in PBS containing 5% DMSO. After custom (200 s) and baseline steps (60 s) with PBS containing 5% DMSO, the biosensors were immersed in wells containing different concentrations of artesunate for binding (200 s) before a dissociation step (120 s). Finally, the data were evaluated by Octet data analysis software.

### In vitro ubiquitination assay

HA-ub-K48 or HA-ub-K63 protein were purified from 293T cells by immobilization with anti-HA magnetic beads (MCE, HY-K0201) and eluted with HA peptides (MCE, HY-P0239) in TBS buffer. The DDX39B ubiquitylation reaction mixture was composed of 150 μM HA-ub-K48 or HA-ub-K63, 150 nM UBE1 (Proteintech, Ag8920), 300 nM UBE2D1 (Proteintech, Ag1932), 350 nM His-TRIM28 or His-TRIM28-C65A, and 4 μM GST-DDX39B in a reaction buffer (50 mM Tris-HCl (pH = 7.5), 2 mM ATP, 50 mM NaCl, 5 mM MgCl_2_, 1 mM DTT) and incubated at 37 °C for 1 h. The reactions were stopped by adding an equal volume of 2× loading buffer and boiling for 10 min, followed by Western blotting.

### Protein‒protein docking

The crystal structure of DDX39B (PDB ID: 1XTI) was obtained from the Research Collaboratory for Structural Bioinformatics Protein Data Bank (RCSB PDB). The TRIM28 crystal structure was constructed based on the I-TASSER server (https://zhanglab.ccmb.med.umich.edu/I-TASSER). Protein‒protein docking was carried out by an online server (http://zdock.umassmed.edu/), and docking parameters were adjusted to default settings. For the molecular dynamic simulation, the top conformation was selected. The binding free energy and energy component for the DDX39B/TRIM28 complex was computed from molecular dynamics simulation trajectories.

### DDX39B-artesunate docking

The crystal structure of DDX39B (PDB: 1XTI) was obtained from the Protein Data Bank.

The crystal structure of DDX39B (PDB: 1XTI) was obtained from the Protein Data Bank. The molecular structure of artesunate was derived from PubChem Compound (https://pubchem.ncbi.nlm.nih.gov/). The protonation state of artesunate was adjusted to pH = 7.4, and the compound was expanded to 3D structures using Open Babel. The receptor protein (DDX39B) and ligand (artesunate) were prepared and parameterized using AutoDock Tools (ADT3). The docking grid documents were constructed by AutoGrid of sitemap, and AutoDock Vina (1.2.0) was applied to simulate docking. The most appropriate docking pose was chosen to analyze the interaction. Finally, the protein‒ligand interactions were plotted using PyMOL.

### Statistical analysis

The corresponding figure legends were described in the data presentation form, and the statistical tests for the results were shown. Graphs were plotted and analyzed with GraphPad Prism, SPSS, Octet data analysis software or Biacore X100 system. At least three independent replicates of the in vitro experiments were performed. Statistical significance was indicated as *P < 0.05, **P < 0.01 and ***P < 0.001.

## Supplementary information

Supplementary Materials

Supplementary Fig. 1

Supplementary Fig. 2

Supplementary Fig. 3

Supplementary Fig. 4

Supplementary Fig. 5

Supplementary Fig. 6

Supplementary Fig. 7

Supplementary Fig. 8

Supplementary Fig. 9

Supplementary Fig. 10

Table S3. IP-MS data of DDX39B interactome

Table S4. The energy decomposition of the DDX39B-TRIM28 complex

Table S5. The potential ubiquitination sites of DDX39B

The original and uncropped films of Western blots

## Data Availability

Experimental data supporting the conclusions of this study are available within the Article and the Supplementary Information. The mass spectrometry data for identification of the ubiquitination sites of DDX39B have been deposited to the ProteomeXchange Consortium (https://proteomecentral.proteomexchange.org) via the iProX partner repository with the dataset identifier PXD064812. All other data supporting the findings of this study are available from the corresponding author upon reasonable request.
