# Supplementary material for: DDX39B K63-linked ubiquitination mediated by TRIM28 promotes NSCLC metastasis by enhancing ECAD lysosomal degradation
Source: Signal Transduct Target Ther. 2025 Jul 16;10:221. doi: 10.1038/s41392-025-02305-9 (PMC12263876; doi:10.1038/s41392-025-02305-9)
Supplement: Supplementary file 1 — Supplementary Materials [file 41392_2025_2305_MOESM1_ESM.docx]

Supplementary Materials for

DDX39B K63-linked ubiquitination mediated by TRIM28 promotes NSCLC metastasis by enhancing ECAD lysosomal degradation

Hang Yuan ^1,5^, Qin Li ^1,5^, Liang Li ^1,5^, Gang Zhao ^2^, Jie Zhang ^1^, Tianyu Feng ^1^, Yafei Guo ^3^, Qiming Kou ^1^, Siqi Li ^1^, Shan Li ^1^, Minghui Zhao ^1^, Guanru Wang ^1^, Qijing Wang ^1^, Jie Qu ^1^, Huayang Yu ^1^, Hongbai Chen ^1^, Lunxu Liu ^4*^, Kai Li ^1*^, Ping Lin ^1*^

Correspondence to: linping@scu.edu.cn

**This PDF file includes:**

Supplementary Materials and Methods

Figs. S1 to S10

Tables S1-2, S6-7

Legends for Tables S3-5

**Other Supplementary Materials for this manuscript include the following:**

Tables S3 to S5

**Supplementary Materials and Methods**

**Cell culture and establishment of the EMT model**

The HEK-293T (ATCC, CRL-1573), NSCLC cell lines A549 (ATCC, CCL-185) and H1299 (ATCC, CRL-5803) were cultured in DMEM (Gibco, 12800-017) or RPMI-1640 (Gibco, 31800-022), respectively, supplemented with 10% FBS and the necessary quantity of penicillin‒streptomycin. The incubation was carried out in a humidified chamber with 5% CO_2_ at 37°C. For EMT model construction, A549 and H1299 cells were starved overnight. Subsequently, the cells were treated with 30 ng/ml EGF (Peprotech, PHG0311), 10 ng/ml TGF-β (ACROBiosystems, TG1-H4212) or 1% BSA (CST, 9998) as a control three times for 72 h with 0.5% FBS in RPMI-1640 medium. EMT was confirmed based on the observation of EMT-like cell morphology, characterized by a spindle-shaped and fibroblast-like appearance, as well as the alteration of EMT markers.

**The isolation and purification of brain metastatic cells**

We developed a carotid artery injection model as has been noted and using bioluminescence imaging to select mice with brain metastasis (BM). Following euthanasia, brain lesions were finely minced and immersed in 0.25% trypsin under sterile conditions. The samples were incubated at 37°C for 15 minutes with gently blown every 5 min, then centrifuged (1000 rpm, 5 min) and resuspended in RPMI-1640 (10% FBS, 1% penicillin‒streptomycin). Cells were then cultured in 10 cm² culture dishes to allow for growth, and selected with G-418 (the corresponding antibiotics for pLV-Neo-CMV-luciferase vector). Subsequently, the purified BM cells were reintroduced into mice for two rounds to generate A549-luc-BM cells.

**Plasmids, transfection and virus production**

The pcDNA3.1-HA tag vector (Addgene, 128034), pRK7-Myc tag vector (Addgene, 119400), pDEST40-2XHA-wtSrc (Addgene, 140294), pET28a-His tag vector (EMD Biosciences, 69864-3), pEnter-Flag-His-vector (Vigenebio, N/A), pEnter-Flag-His-DDX39B (Vigenebio, CH821820), and pEnter-Flag-His-TRIM28 (Vigenebio, CH831297) were purchased. The pGEX-5X-3-GST tag vector was kindly provided by Qunyin Lei (Fudan University). Flag-ub, HA-ub, HA-ub-K48, and HA-ub-K63 were kindly provided by Hongbo Hu (Sichuan University). pCAGGS-TRIM28-ΔRING-HA, pCAGGS-TRIM28-HA were kindly provided by Xiaofeng Guo (South China Agricultural University). HA-ub-K6, HA-ub-K11, HA-ub-K27, HA-ub-K29, HA-ub-K33 were purchased (Miaoling biology, P31806, P31913, P31987, P31814, P31798). The pBiFc-VN173 vector and pBiFc-VC155 vector plasmids were gifts from Chang-Deng Hu (Addgene, 22010 and 22011). The pcDNA3.1-HA-Hakai vector was kindly provided by Yasuyuki Fujita (Max-Delbrück-Center for Molecular Medicine, Germany). The pRL-TK and TOP/FOP Flash were kindly provided by Jinyi Liu (Third Military Medical University). The pET28a-His-TRIM28 plasmid was synthesized by Sangon Biotech. The plasmids or lentivirus of shRNAs targeting DDX39B and TRIM28 were synthesized and purchased from GeneChem. The shRNAs targeting Hakai, pLV[Exp]-Puro-EF1A>Flag-DDX39B (wild type), pLV[Exp]-Puro-EF1A>Flag-DDX39B (P322A), pLV[Exp]-EGFP:T2A:Hygro-CMV>HA-CDH1 and pLV-Neo-CMV-luciferase vector plasmids and lentivirus in this study were synthesized and purchased from VectorBuilder. The detailed shRNA sequences are provided in **(Table S6)**. The full-length human cDNA of DDX39B (NM_004640.7) was cloned and inserted into the pRK7-Myc tag vector. DDX39B, TRIM28 and ECAD cDNA were cloned and inserted into pBiFc-VN173 vector or pBiFc-VC155 vector, respectively. His-tagged DDX39B and TRIM28 (NM_005762.3) expression in *Escherichia coli* was based on the pET28a-His tag vector backbone. GST-tagged DDX39B (WT, P322A, 3KR), TRIM28 and ECAD cytoplasmic domain (ECD) (731-882 aa) expression in *Escherichia coli* was based on the pGEX-5X-3-GST tag vector backbone. The expression of His-tagged DDX39B mutants (F127A, E134A, S137A, L152A, P322A) or His-tagged TRIM28 mutant (C65A) in *Escherichia coli* was based on the pET28a-His-DDX39B backbone. The point mutations of DDX39B (K95R, K131R, K144R, K191R, K241R, K268R, K384R, K398R, and 3KR (K241R, K384R and K398R)) were was based on the pRK7-Myc-DDX39B backbone. All mutants were generated using the site-specific mutagenesis method (TransGen Biotech, FM111-01). Mutations were confirmed by sequencing services (Sangon Biotech). Transfection of the indicated plasmids into A549 and H1299 cells was performed by using Lipofectamine 3000 (Invitrogen) in accordance with the protocol recommended by the manufacturer. For the establishment of stable cell lines, cells were infected with the indicated lentiviruses alone or in combination with fresh RPMI-1640 medium (10% FBS) containing 4 μg/ml polybrene as described previously. After culturing for four days, the cells were screened and established with the corresponding antibiotics (puromycin, hygromycin or G-418).

**Antibodies, drugs, and other reagents**

Detailed information on all antibodies, drugs and other reagents was provided in **(Table S7).**

**RT‒qPCR**

Total RNA was isolated with TRIzol (Tarkara, 9109) and reverse transcribed with 1.5 μg by using a HiScript^®^Ⅱ Q Select RT SuperMix for qPCR kit (Vazyme, R232-01). Relative gene expression was assessed by RT‒qPCR using a CFX Connect real-time PCR system (Roche) in accordance with the protocol recommended by the manufacturer. All analyses were performed in triplicate. The expression of mRNAs was estimated on the basis of the threshold cycle (Ct), and the relative abundance of mRNAs was calculated using the 2^-∆∆Ct^ method and normalized to 18S. Sequences for qPCR primers are shown in **(Table S6).**

**Western blotting**

The indicated cells were harvested and lysed in lysis buffer (150 mM NaCl, 50 mM Tris-HCl (pH=7.4), 1% Triton X-100, 1 mM EDTA), protease (Thermo Fisher, 78430) and phosphatase inhibitor (Thermo Fisher, 78428). Then, the cell lysates were sonicated at 40% duty for 2 min (5 s on, 15 s off) on ice. The protein concentration was measured by a BSA protein assay kit (Sangon Biotech, C503021). Equal amounts of protein (10-40 μg) were fractionated by 8-12% SDS–polyacrylamide gel electrophoresis. Subsequently, the proteins were transferred to polyvinylidene fluoride (PVDF) membranes (Merck Millipore, IPVH00010) and blocked with 5% skim milk in TBS (containing 0.05% Tween 20) at room temperature for 1 h. The membranes were incubated with the primary antibody at 4°C overnight and with HRP-conjugated secondary antibody at room temperature for 1 h. Detailed information on the antibodies is provided in **Table S7.**

**Immunohistochemistry (IHC) staining**

IHC analysis was performed as previously described (56). Briefly, the isolated tumor tissues were promptly immersed in 4% paraformaldehyde at room temperature. Paraffin-embedded tissues were sectioned at 4 μm. The slices were incubated overnight at 4°C with the following primary antibodies: DDX39B (Sangon Biotech, BB25AA0014), ECAD (Cell Signaling Technology, 3195), EPCAM (Sangon Biotech, D226394), NCAD (Cell Signaling Technology, 13116), Vimentin (Cell Signaling Technology, 5741), β-catenin (Huabio, ET1601-5). After incubation with HRP-conjugated secondary antibodies for 1 h at room temperature, detection was performed using a DAB Detection Kit (Gene Tech, GK600510). Later, the nuclei were counterstained with hematoxylin (Beyotime, C0105M).

**Surface plasmon resonance (SPR) assay**

The SPR screening of artesunate targeting DDX39B was accomplished by a Biacore X100 system (GE Healthcare). All assays were carried out at 30°C. SPR kinetic analysis was conducted according to the method suggested in the instruction manual using HBS-P running buffer (10 mM HEPES (pH=7.4), 150 mM NaCl, 0.05% (v/v) surfactant P20), and the His-DDX39B protein was loaded onto a CM5 chip (Cytiva, BR100012) on a Biacore X100 SPR instrument (GE Healthcare). After a baseline step (60 s) with PBS buffer (5% DMSO), multiple concentrations of artesunate in PBS containing 5% DMSO were injected on the chip surface (90 s), followed by a dissociation step (60 s). The kinetic analyses were performed on the basis of the steady-state affinity fit model, according to the methods detailed in the software manual.

**Cellular thermal shift assay (CETSA)**

A549 and H1299 cells were lysed by sonication, cell lysates were centrifuged, and the supernatants were collected. Aliquots were incubated with artesunate (100 µM) or solvent control (DMSO) for 1 h at room temperature. Samples were aliquoted to a volume of 40 µl/tube and exposed to graduated temperatures for 3 min, cooled at room temperature for 3 min, and maintained on ice. The samples were submitted to centrifugation, and the supernatant (soluble fractions) was then evaluated by Western blotting.

**Immunofluorescence analysis**

A549 and H1299 cells seeded on coverslips were fixed with 4% paraformaldehyde in PBS for 10 min at room temperature. Cells were washed with PBS buffer 2 times and permeabilized with 0.5% Triton-X-100 for 7 min at room temperature. After washing with PBS buffer 2 times, the cells were blocked with blocking buffer (5% horse serum in PBS buffer) before incubation with primary antibodies (recommended dilution) at 4 °C overnight. Coverslips were incubated with appropriate fluorescence-labeled secondary antibodies (1:100) (Proteintech, SA00003-1) (Invitrogen, A11037) (Proteintech, SA00003-2) (Proteintech, SA00007-1) after 3 washes with PBS buffer. The nuclei were stained with DAPI (Solarbio, C0065) for 5 min, the coverslips were then mounted with anti-fade mounting medium (Sangon Biotech, E675011), and images were acquired with a fluorescence confocal microscope (Nikon, N-SIM S). For F-actin staining, cells were stained with rhodamine-phalloidin (US Everbright^®^ Inc., YP0063) according to the manufacturer’s instructions. Detailed information on the antibodies is given in **(Table S7).**

**Subcellular fractionation**

Cells were grown on 10 cm^2^ dishes, and the subcellular fractions were harvested according to the protocol recommended by the manufacturer. The cytosolic fraction and nuclear fraction were prepared by using a nuclear and cytoplasmic extraction kit (CWBIO, CW0199). The lysosome fraction was obtained with a lysosome extraction kit (Bestbio, BB-3603). Finally, the samples were prepared by adding 5× loading buffer and boiling for 10 min, followed by Western blotting.

**Transwell migration/invasion assay**

Transwell migration/invasion assays were performed by using 8-μm pore filters (Corning, 3422). The bottom chamber was resupplied with 400 μl of 10% FBS RPMI-1640 medium. For the transwell migration assay, 200 μl of FBS-free 1640 medium containing 3×10^5^ A549 or 2×10^5^ H1299 cells was added to the upper chamber, while 3.5×10^5^ A549 or 2.5×10^5^ H1299 cells were prepared for the invasion assay in a chamber precoated with 100 μl Matrigel (1:30) (Corning, 356234). Cells were cultured for 24 h (migration) or 48 h (invasion) at 37°C, and the upper chamber was immersed in 4% paraformaldehyde for 15 min at room temperature. Subsequently, the membranes were stained with 0.5% crystal violet for 18 min, and unmigrated cells were carefully wiped off with a cotton swab. The migratory and invasive cells were imaged under a microscope and counted using ImageJ software.

**Wound closure assay**

Cells were plated in 6-well plates and cultured in RPMI-1640 with 10% FBS when the cells attained 90% confluence. A scratch was generated with a 10 μl sterile pipette tip and washed with PBS buffer 2 times and captured (0 h) with micrographs. Thereafter, cells were cultured in FBS-free RPMI-1640 medium for 24 h. The migration of cells was monitored with micrographs at 24 h. The cell migration area was calculated by ImageJ software.

**Cell adhesion assay**

For the adhesion assay, Matrigel was precoated onto a 96-well plate for 2 h at 37°C. Single-cell suspensions of A549 (1×10^5^) and H1299 (1×10^5^) cells were prepared in 100 μl of FBS-free RPMI-1640 medium per well and incubated at 37°C for indicated time. The adherent cells were captured by a microscope and counted using ImageJ software after washing 2 times with PBS buffer.

**3D-spheres formation assay**

Matrigel (50 μl) was precoated in a 96-well plate at 37°C for 30 min. Single-cell suspensions of cells (500 cells per well, 50 μl) were prepared in MEM complete media and added to the precoated plate at 37°C for 30 min. Afterward, the Matrigel was diluted using MEM complete medium (Matrigel: medium= 1:10), and 50 µl of culture medium containing Matrigel was slowly pipetted into the above cells. MEM complete media was added for sphere formation every 2 days for 5-10 days. The spheres were captured by a microscope and measured by using ImageJ software.

**TOP/FOP Flash assay**

For the TOP/FOP Flash assay, the TOP-Flash or FOP-Flash reporter was co-transfected with pRL-TK plasmids into the indicated A549 and H1299 cells in 24-well plates for 48 h. According to the manufacturer's instructions for the Dual-Luciferase^®^ Reporter Assay System (Promega, E1960), luciferase activity was assessed in cell extracts. The relative luciferase intensity was then standardized to the activity of pRL-TK (Renilla luciferase) by a formula ((TOP/RL ratio)/ (FOP/RL ratio)).

**Supplementary Figures**

**Figure S1**


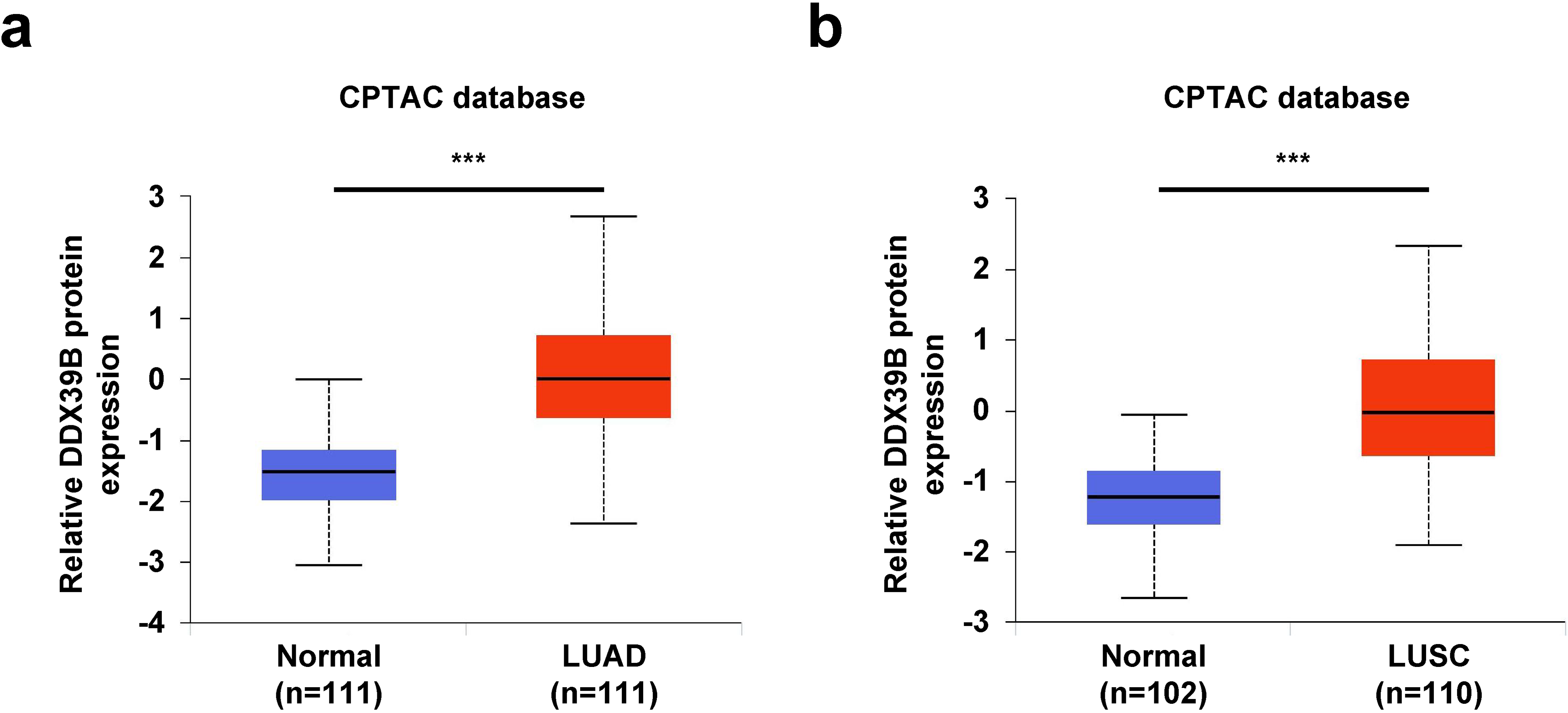


**Figure S1. DDX39B were upregulated in NSCLC patients.**

**(a-b)** The protein expression of DDX39B in **(a)** lung adenocarcinoma (LUAD) (n=111) compared with normal tissues (n=111), and in **(b)** lung squamous cell carcinoma (LUSC) (n=110) compared with normal tissues (n=102) based on the CPTAC database by an online tool  (<http://ualcan.path.uab.edu/index.html>). ****P* < 0.001.

**Figure S2**


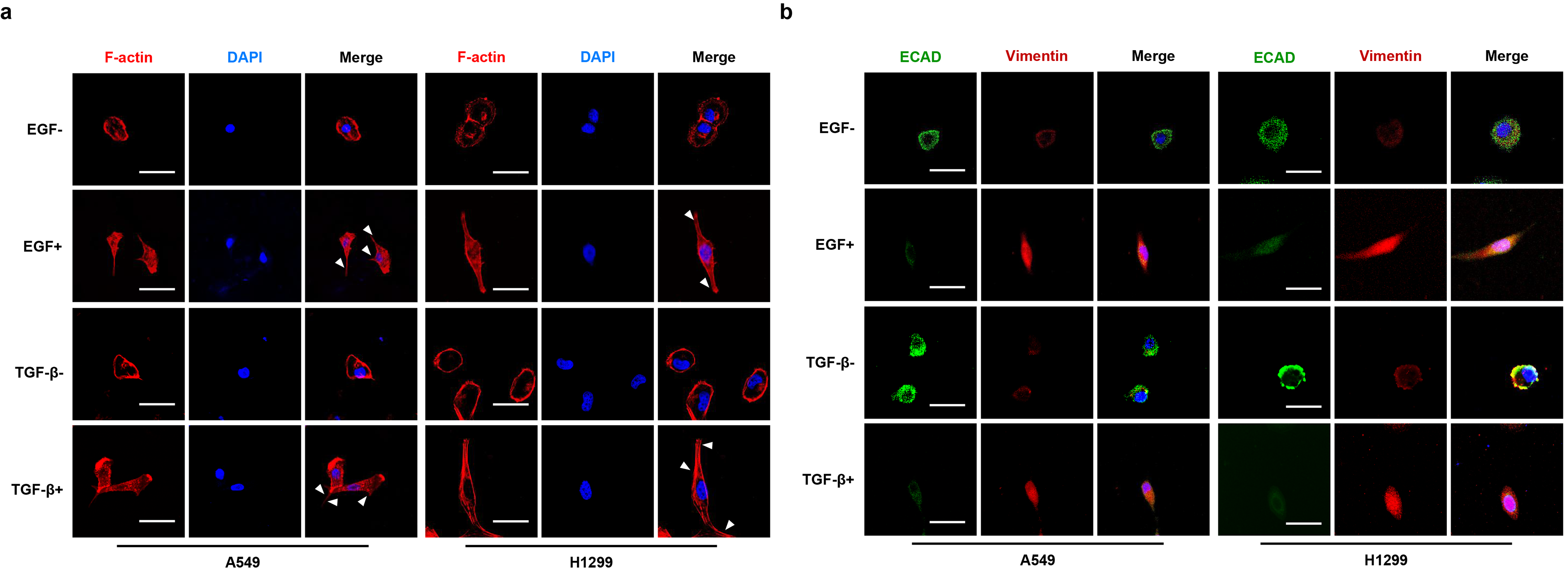


**Figure S2. EMT-metastasis models were established.**

NSCLC cell lines (A549 and H1299) were starved overnight and then treated with or without 30 ng/ml EGF or 10 ng/ml TGF-β for 72 h. **(a)** F-actin and nuclei were stained using rhodamine-phalloidin and DAPI, respectively. Representative images are shown, and the white triangles represent cilia and pseudopodia. **(b)** Costaining of ECAD and Vimentin by immunofluorescence staining in NSCLC cells. Scale bars, 50 µm.

**Figure S3**


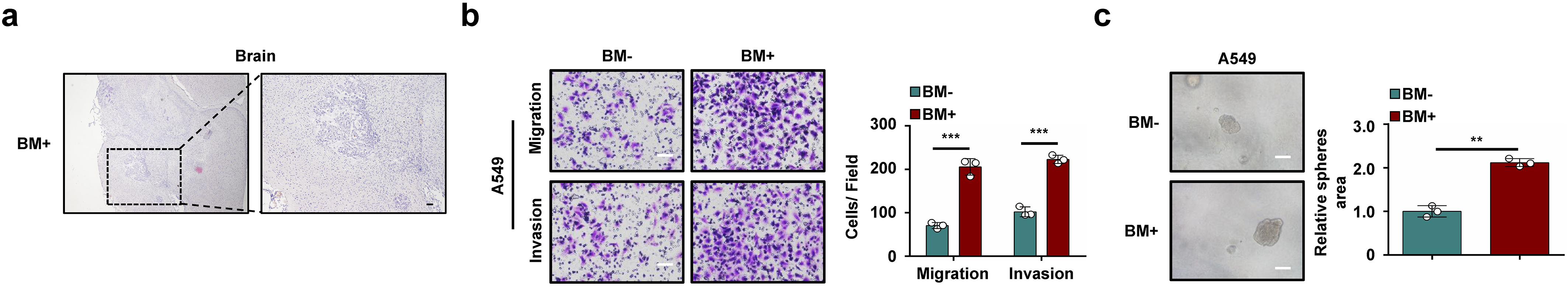


**Figure S3. Brain-metastasis model was constructed.**

**(a)** Representative images of H&E staining of brain metastatic tissues were shown. **(b)** The cell migration, invasion, **(c)** 3D sphere formation of indicated cell lines were analyzed (n=3). Scale bars, 50 µm. *P* values were determined by a two-tailed unpaired *t* test **(b-c)**. ***P* < 0.01, ****P* < 0.001.

**Figure S4**


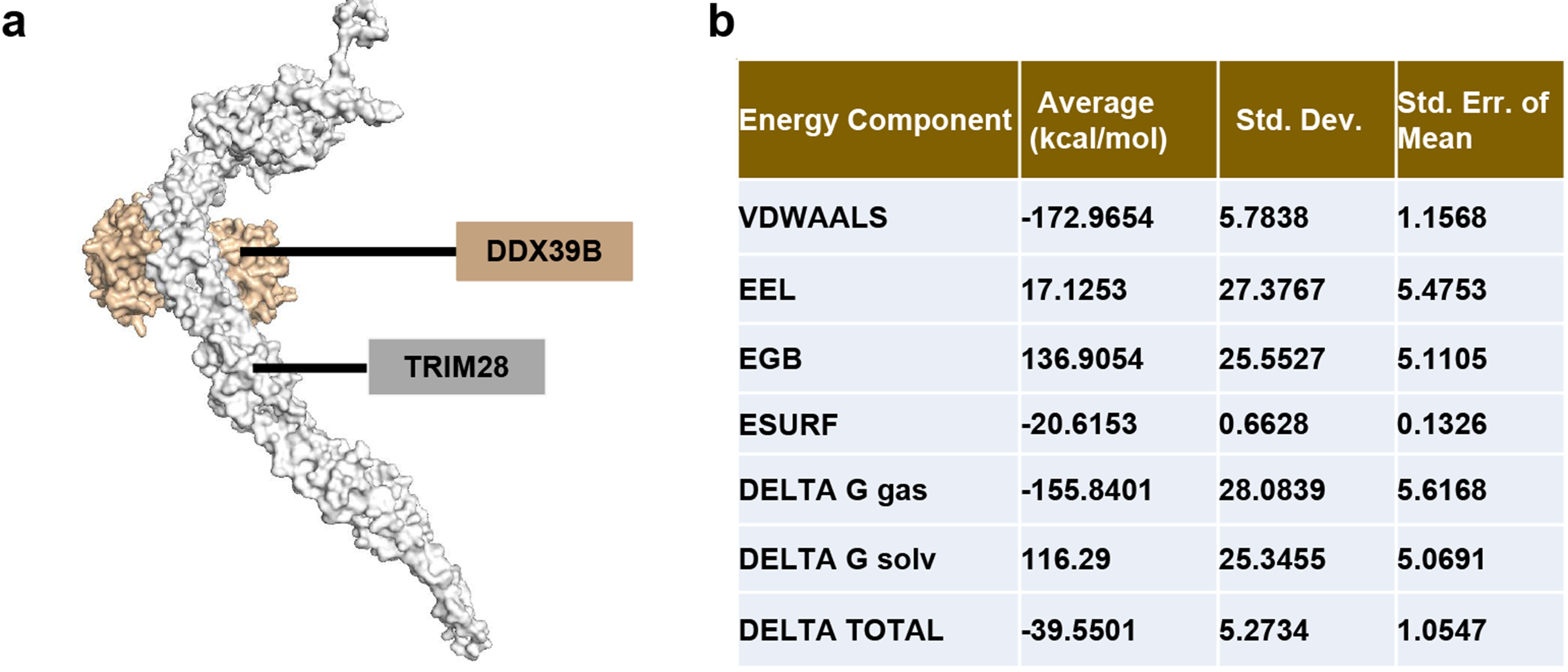


**Figure S4.** **The molecular docking model of DDX39B and TRIM28 protein.**

**(a)** Primary conformation model for molecular docking of DDX39B with TRIM28 protein. **(b)** The energy decomposition of the DDX39B/TRIM28 complex was calculated.

**Figure S5**


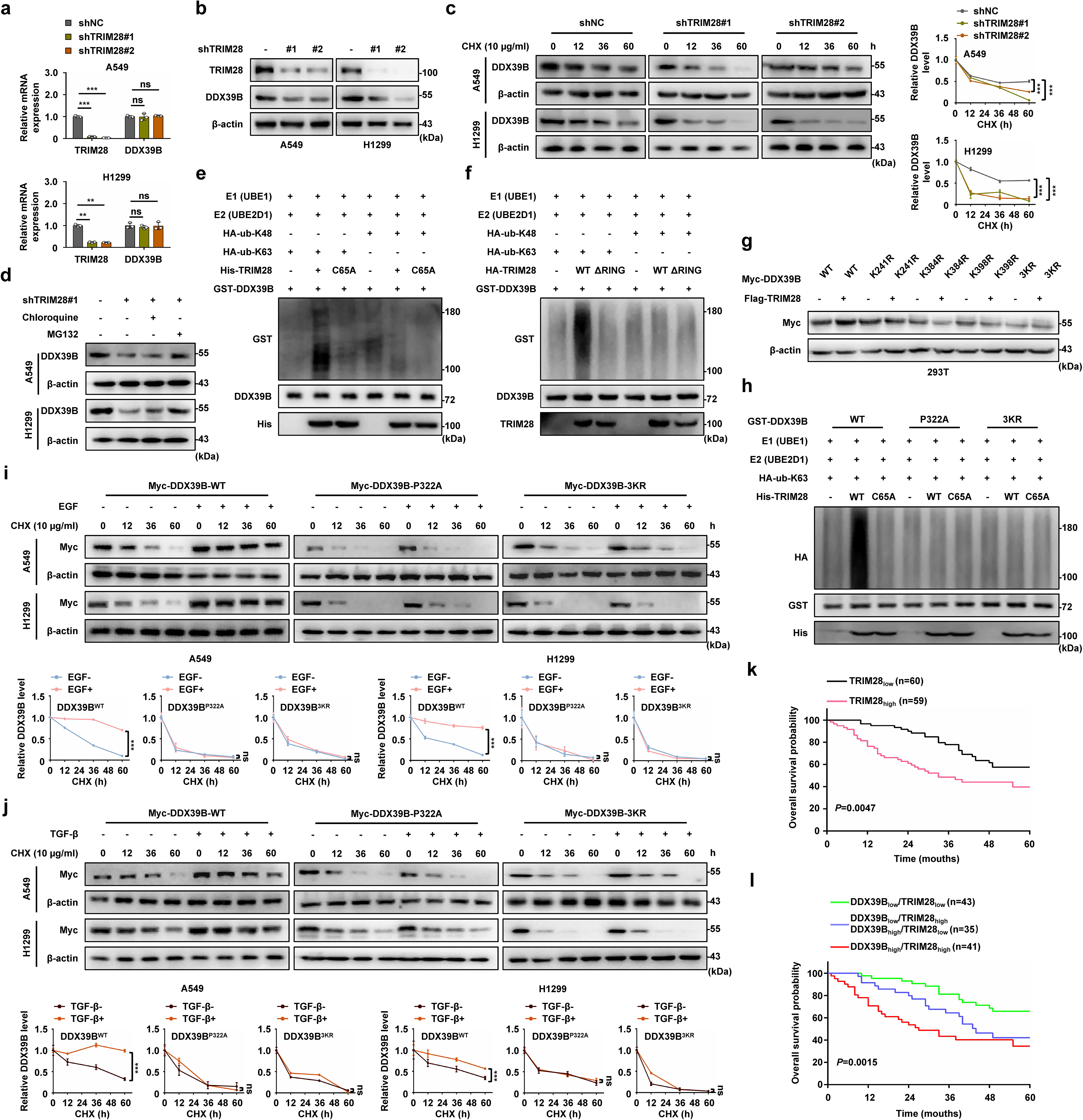


**Figure S5. TRIM28 was identified as a potential E3-specific ligase of DDX39B to stabilize DDX39B.**

**(a-b)** The effects of TRIM28 knockdown on **(a)** DDX39B mRNA (n=3) or **(b)** protein expression in NSCLC cell lines (A549 and H1299) was determined by RT‒qPCR and Western blotting, respectively. **(c)** The indicated cells were treated with 10 μg/ml CHX for the indicated times (0, 12, 36, and 60 h), and DDX39B protein level was examined by Western blotting. The ratio of DDX39B to β-actin was calculated. The line graph represents the rate of DDX39B degradation (n=3). **(d)** TRIM28-knockdown cells were treated with chloroquine or MG132, and DDX39B expression was detected by Western blot analysis. **(e)** The effect of TRIM28 WT or C65A (no enzymatic activity) mutant on the K48- or K63-linked ubiquitination of purified GST-DDX39B protein was examined by *in vitro* ubiquitination assay. **(f)** The effect of TRIM28 WT or ΔRING (RING domain deletion) on the K48- or K63-linked ubiquitination of purified GST-DDX39B protein was examined by *in vitro* ubiquitination assay. **(g)** 293T cells were co-transfected with Flag-TRIM28 and Myc-DDX39B WT or indicated mutant DDX39B, followed by immunoblotting with indicated antibodies. **(h)** The impact of TRIM28 WT or C65A (no enzymatic activity) mutant on the K63-linked ubiquitination of purified DDX39B WT, P322A or 3KR was examined by *in vitro* ubiquitination assay.**(i-j)** Cells transfected with DDX39B WT, P322A or 3KR (K241R, K384R and K398R) mutant were stimulated with or without **(i)** EGF or **(j)** TGF-β, and then treated with CHX (10 μg/ml) for the indicated times (0, 12, 36 and 60 h). The ratio of DDX39B to β-actin was calculated. The line graph represents the rate of DDX39B degradation (n=3). **(k)** Kaplan‒Meier estimates of overall survival probability based on TRIM28 protein expression in NSCLC patients (n=119). **(l)** Prognostic value of DDX39B combined with TRIM28 expression in NSCLC patients (n=119). Graphs represent data as the mean ± s.d. *P* values were determined by one-way ANOVA **(a)**, two-way ANOVA **(c, i-j)** or log-rank test **(k-l)**. ns, not significant, ***P* < 0.01, ****P* < 0.001.

**Figure S6**


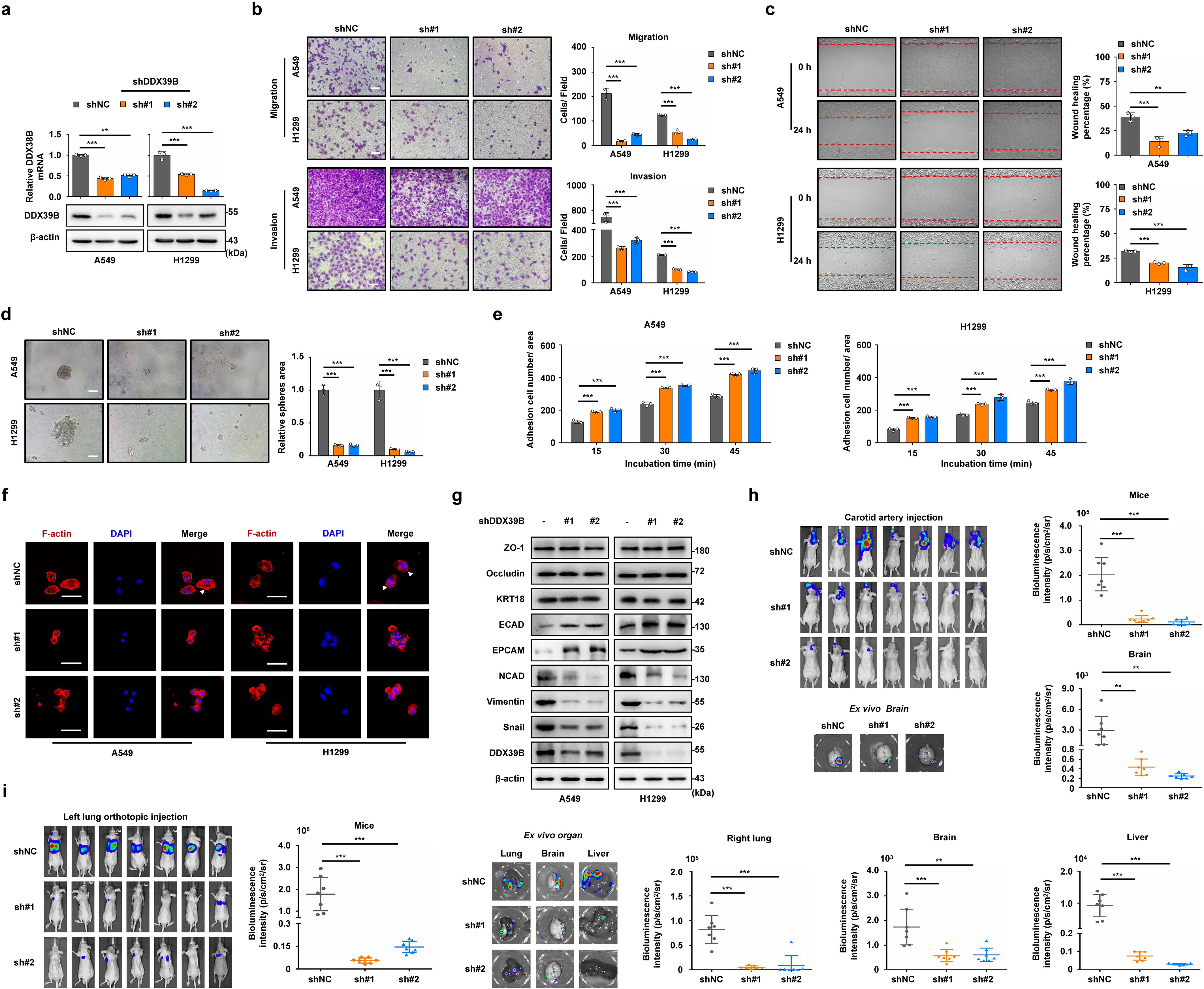


**Figure S6: DDX39B knockdown suppresses migration, invasion and EMT-like programs in NSCLC cells.**

**(a)** The efficiency of DDX39B knockdown by two different shRNAs in NSCLC cell lines (A549 and H1299) was determined by RT‒qPCR (n=3) and Western blotting, respectively. **(b-e)** The effects of DDX39B knockdown on **(b)** cell migration, invasion, **(c)** wound closure, **(d)** 3D sphere formation and **(e)** adhesion were examined (n=3). **(f)** F-actin and nuclei were stained using rhodamine-phalloidin and DAPI, respectively. Representative images were shown, and the white triangles represented cilia and pseudopodia. **(g)** The protein expression of mesenchymal markers (N-cadherin, Vimentin and Snail) and epithelial markers (ZO-1, Occludin, KRT18, ECAD and EPCAM) in DDX39B-silenced NSCLC cells were examined. **(h)** Indicated A549 cells were injected into carotid artery. Six weeks later, representative bioluminescence images and intensities (p/s/cm2/sr) of mice and isolated brain were presented and quantified (n=7/group). **(i)** Indicated A549 cells were orthotopically inoculated into the left pulmonary tissue. Eight weeks later, the representative bioluminescence images and intensities (p/s/cm2/sr) of mice and isolated organs (including the right lung, brain, and liver) were shown and quantified (n=7/group). Scale bars, 50 µm. Graphs represent data as the mean ± s.d. *P* values were determined by one-way ANOVA **(a-e, h-i)**. ***P* < 0.01, ****P* < 0.001.

**Figure S7**


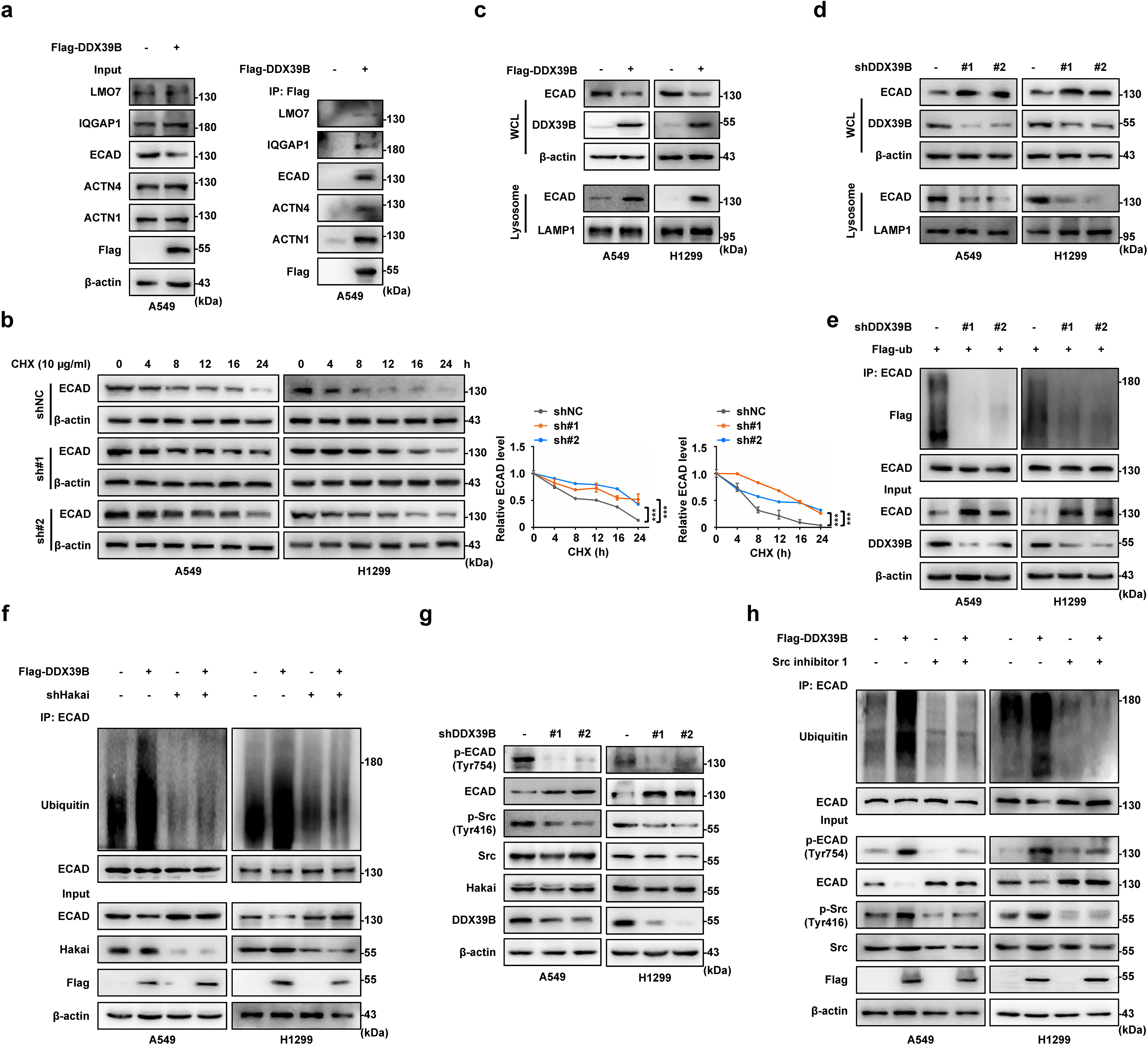


**Figure S7. DDX39B interacts with ECAD and regulates the ubiquitination and lysosomal degradation of ECAD through the Src/Hakai pathway.**

**(a)** The association between DDX39B and its interacting proteins (including ECAD, ACTN1, LMO7, ACTN4, and IQGAP1 proteins) was analyzed by immunoprecipitation assay. **(b)** DDX39B-silenced cells were treated with CHX (10 μg/ml) for the indicated times (0, 4, 8, 12, 16 and 24 h). The ratio of ECAD to β-actin was calculated. The line graph represents the rate of ECAD degradation (n=3). **(c-d)** The effect of **(c)** DDX39B overexpression or **(d)** DDX39B knockdown on ECAD expression in whole cell lysates (WCL) and lysosome was detected by Western blot analysis. **(e)** The indicated cells were transfected with the Flag-ub plasmid. The expression and ubiquitination of ECAD were analyzed. **(f)** DDX39B-overexpressed cells were transfected with or without shRNA targeting Hakai. The expression and ubiquitination of ECAD protein were detected. **(g)** The phosphorylation of ECAD^Y754^ was monitored in DDX39B-silenced NSCLC cells. **(h)** DDX39B-overexpressed cells were treated with Src inhibitor 1 (10 μM). The ECAD ubiquitination and pECAD^Y754^ expression were detected. Graphs represent data as the mean ± s.d. *P* values were assessed by two-way ANOVA **(b)**. ****P* < 0.001.

**Figure S8**
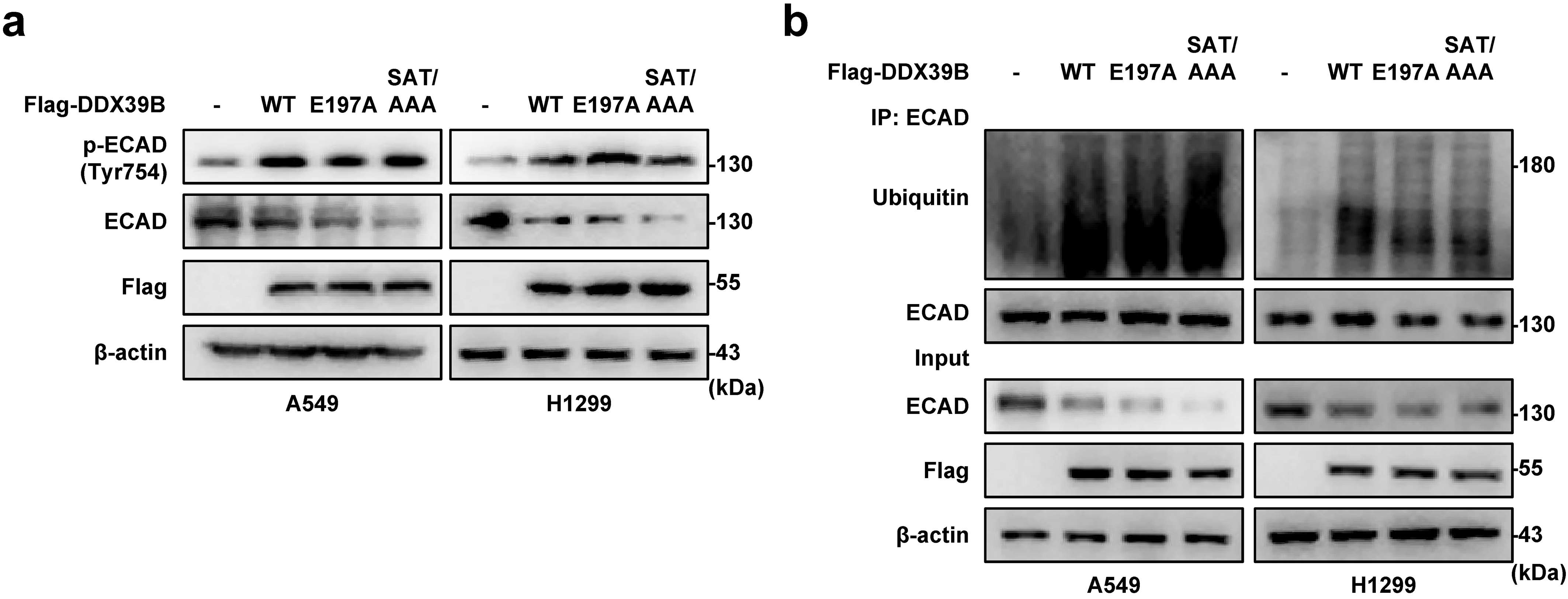


**Figure S8. DDX39B modulated the expression and ubiquitination of ECAD is independent of its ATPase and helicase activity.**

**(a-b)** NSCLC cells were transfected with DDX39B wild-type (WT) or the ATPase and helicase activity-deficient mutants (E197A and SAT/AAA). **(a)** The expression of ECAD, the phosphorylation of ECAD^Y754^ and **(b)** the ubiquitination of ECAD protein were detected.

**Figure S9**


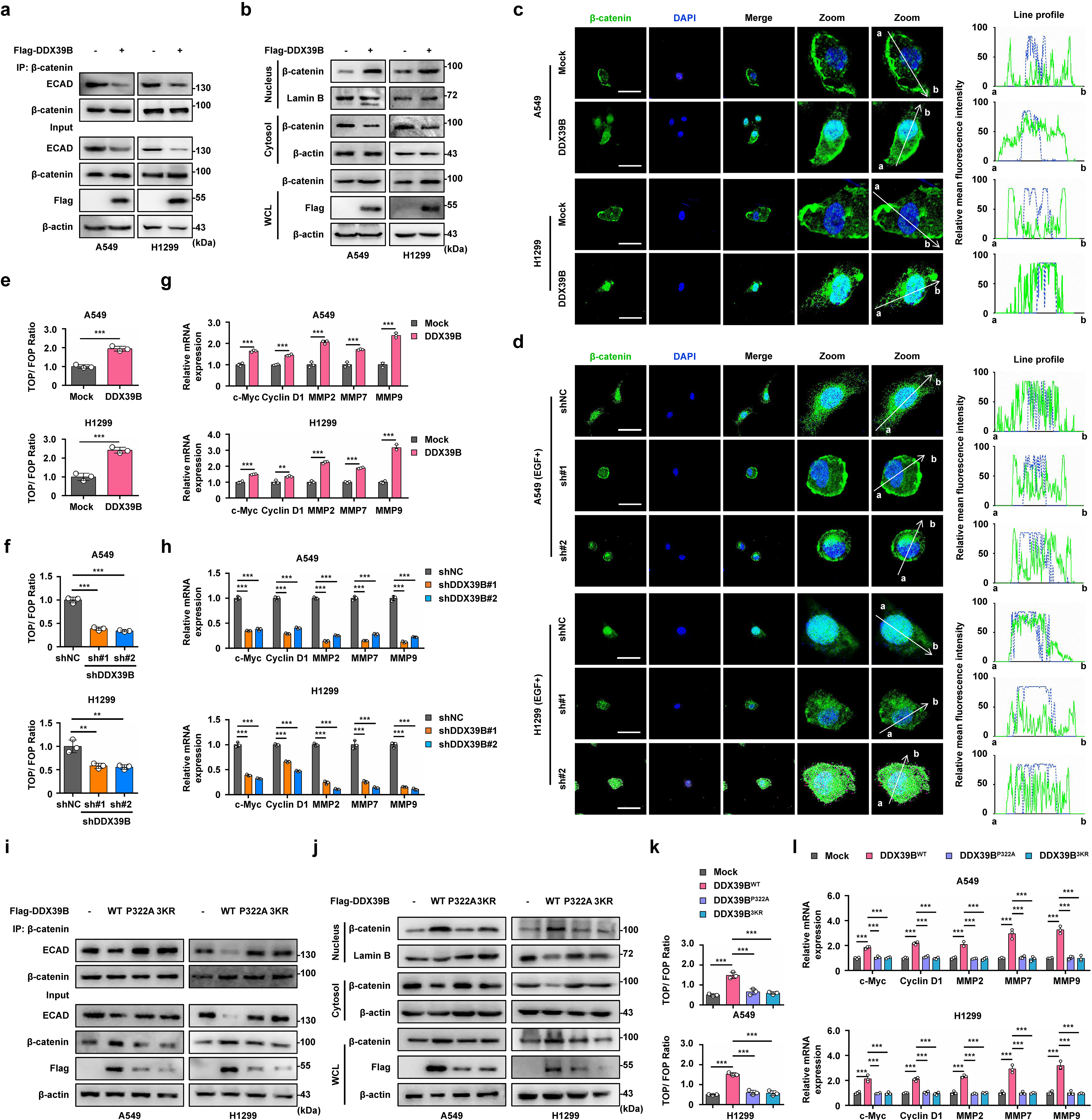


**Figure S9. DDX39B promotes the disassociation of the ECAD/β-catenin complex and facilitates the nuclear localization and targeted-genes transcription of β-catenin.**

**(a)** The effect of DDX39B on the association between ECAD and β-catenin was detected by immunoprecipitation assay. **(b)** β-catenin expression in the indicated cell fractions was detected by Western blot analysis. WCL: whole cell lysates. **(c)** The intracellular distribution of β-catenin in DDX39B-overexpressed NSCLC cells were examined by immunofluorescence staining. **(d)** The indicated NSCLC cells were treated with EGF and the subcellular localization of β-catenin was examined by immunofluorescence staining. The line profiles of β-catenin and DAPI signals were measured by ImageJ software (DAPI: blue, β-catenin: green). **(e-f)** The effect of **(e)** DDX39B overexpression or **(f)** knockdown on the transcriptional activity of β-catenin was analyzed by TOP/FOP-Flash luciferase reporter assay (n=3). **(g-h)** The mRNA expression levels of β-catenin downstream targets (c-Myc, CyclinD1, MMP2, MMP7, MMP9) in **(g)** DDX39B overexpression or **(h)** knockdown NSCLC cells were examined by RT‒qPCR (n=3). **(i-l)** The effects of DDX39B WT, P322A or 3KR (K241R, K384R and K398R) mutant on **(i)** the interaction between ECAD and β-catenin, **(j)** the intracellular distribution of β-catenin, **(k)** the transcriptional activity of β-catenin (n=3) and **(l)** the mRNA expression levels of β-catenin downstream targets (n=3). Scale bars, 50 µm. Graphs represent data as the mean ± s.d. *P* values were determined by two-tailed unpaired *t* test **(e, g)** or one-way ANOVA **(f, h, k-l)**. ***P* < 0.01, ****P* < 0.001.

**Figure S10**


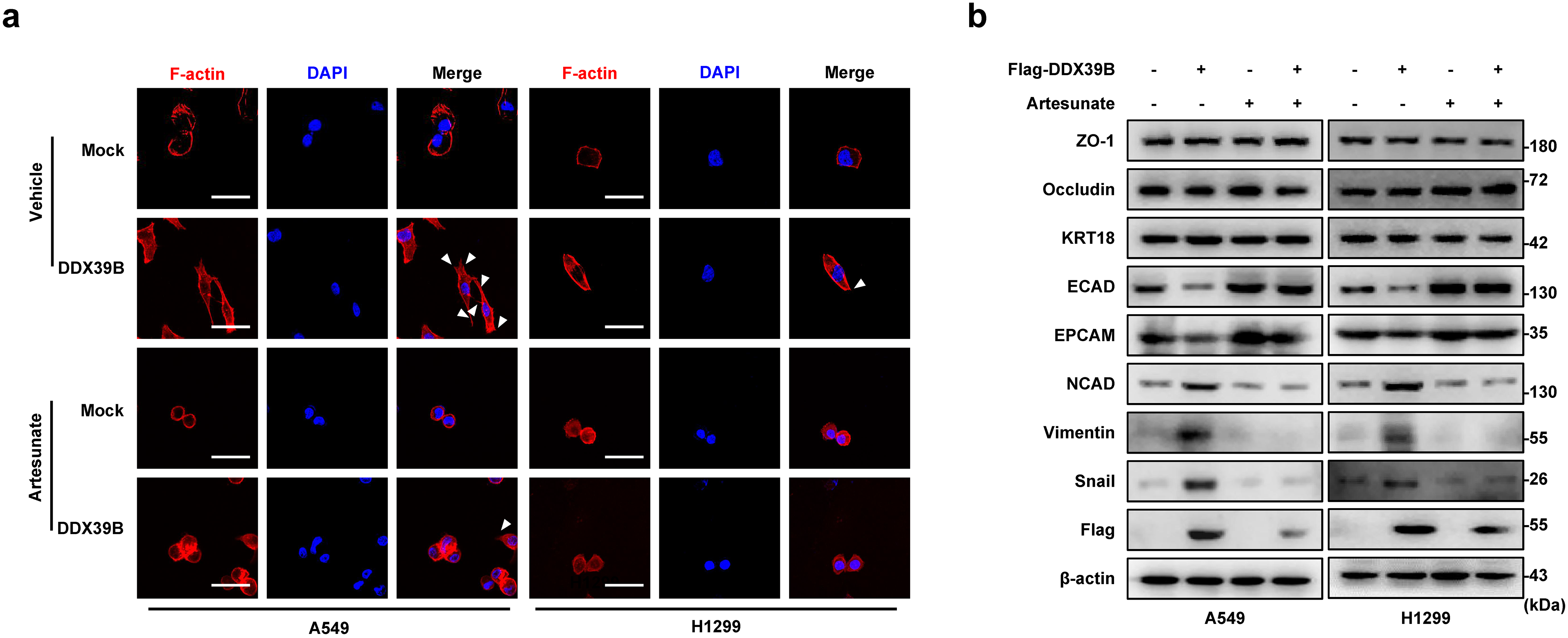


**Figure S10. Artesunate blocks DDX39B-mediated EMT reprogramming in NSCLC.**

DDX39B-overexpressed NSCLC cell lines (A549 and H1299) were treated with or without artesunate (20 μM). **(a)** F-actin and nuclei were stained using rhodamine-phalloidin and DAPI, respectively. Representative images were shown, and the white triangles represented cilia and pseudopodia. **(b)** The expression of EMT markers was detected by Western blot analysis. Scale bars, 50 µm.

**Supplementary Tables**

**Table S1. Association between DDX39B protein levels and clinicopathological variables.**

| Clinic features | DDX39B_low_ | DDX39B_high_ | ꭓ^2^ | *P* value |
| --- | --- | --- | --- | --- |
| Age |  |  | 0.640 | 0.424 |
| ≤ 60 | 36 | 30 |  |  |
| > 60 | 25 | 28 |  |  |
| Gender |  |  | 3.737 | 0.053 |
| Male | 35 | 23 |  |  |
| Female | 26 | 35 |  |  |
| Grade |  |  | 0.606 | 0.739 |
| Well  Moderate | 4  47 | 6  42 |  |  |
| Poor  Tumor size  ≤ 3 cm  > 3 cm | 10  19  42 | 10  24  34 | 1.349 | 0.246 |
| T stage |  |  | 0.960 | 0.327 |
| T1+T2 | 45 | 38 |  |  |
| T3+T4 | 16 | 20 |  |  |
| LN metastasis |  |  | 6.071 | 0.014 |
| No | 39 | 24 |  |  |
| Yes | 22 | 34 |  |  |
| M stage |  |  | 5.745 | 0.017 |
| M0 | 60 | 49 |  |  |
| M1 | 1 | 9 |  |  |
| TNM stage |  |  | 11.731 | 0.008 |
| I | 27 | 13 |  |  |
| II  III  IV | 18  15  1 | 17  19  9 |  |  |

**Table S2. Univariate and multivariate analysis of factors associated with survival in NSCLC patients.**

| Variable | Univariate analysis | | Multivariate analysis | |
| --- | --- | --- | --- | --- |
|  | HR (95%CI) | *P* value | HR (95%CI) | *P* value |
| Age (> 60 vs. ≤ 60 years) | 1.243 (0.737-2.097) | 0.414 |  |  |
| Gender (female vs. male) | 1.109 (0.660-1.866) | 0.695 |  |  |
| Grade (poor vs. well/moderate) | 1.386 (0.718-2.677) | 0.331 |  |  |
| Tumor size (> 3 vs. ≤ 3 cm) | 1.308 (0.747-2.290) | 0.347 |  |  |
| T stage (T3+T4 vs. T1+T2) | 2.666 (1.575-4.513) | <0.001 |  |  |
| LN metastasis (yes vs. no) | 4.609 (2.570-8.265) | <0.001 | 2.210 (1.044-4.676) | 0.038 |
| M stage (M1 vs. M0) | 1.850 (0.792-4.320) | 0.155 |  |  |
| TNM stage (III+IV vs. I+II) | 5.095 (2.940-8.830) | <0.001 | 2.835 (1.402-5.732) | 0.004 |
| DDX39B (high vs. low) | 2.311 (1.353-3.947) | 0.002 | 1.932 (1.125-3.317) | 0.017 |

LN, lymph node.

**Table S3. IP-MS data of DDX39B interactome.**

Data provided in Excel file Table S3.

**Table S4. The energy decomposition of the DDX39B-TRIM28 complex.**

Data provided in Excel file Table S4.

**Table S5. The potential ubiquitination sites of DDX39B.**

Data provided in Excel file Table S5.

**Table S6. Sequences of the oligonucleotides for shRNA, RT‒qPCR, plasmid constructions.**

| Usage | Name | Sequences (5' to 3') |
| --- | --- | --- |
| shRNAs | shNC | TTCTCCGAACGTGTCACGT |
|  | shDDX39B#1 | TAGACATCTCCTCCTACAT |
|  | shDDX39B#2 | CCGCAAGTTCATGCAAGAT |
|  | shTRIM28#1 | CTGAGACCAAACCTGTGCTT |
|  | shTRIM28#2 | ATGGTGAACGTACTGTCTATT |
|  | shHakai | CTCTTATCGAGGTAGTATAAA |
| RT-qPCR | DDX39B | TTTGAGCATCCGTCAGAAGTCC |
|  |  | CCAGTACAGACACCTGCCCAGT |
|  | 18S | TTGACGGAAGGGCACCACCAG |
|  |  | GCACCACCACCCACGGAATCG |
|  | TRIM28 | GCCGTGTGCTGGTCAATGATG |
|  |  | GAATGTGCTCCTGGTGCTTCTG |
|  | c-Myc | CAGCCCGAGACTGTTGC |
|  |  | CAGAGCGTGGGATGTTAG |
|  | CyclinD1 | GGGGCGATTTGCATTTCTAT |
|  |  | CGGTCGTTGAGGAGGTTGG |
|  | MMP2 | ATTGTATTTGATGGCATCGCTC |
|  |  | ATTCATTCCCTGCAAAGAACAC |
|  | MMP7 | CCAGATGTGGAGTGCCAGATGTTG |
|  |  | CGTCCAGCGTTCATCCTCATCG |
|  | MMP9 | CAGTACCGAGAGAAAGCCTATT |
|  |  | CAGGATGTCATAGGTCACGTAG |
|  | Hakai | GAGCCATATTCCGCCAAAG |
|  |  | ATACGAAAGGTTTCCTGACTGAC |
| Mutant plasmids  constructions | His-DDX39B-F127A | CACACTCGGGAGTTGGCTGCTCAGATCAGCAAGG |
|  |  | GCAGCCAACTCCCGAGTGTGACACATCACCA |
|  | His-DDX39B-E134A | AGATCAGCAAGGAATATGCGCGCTTCTCTAAATA |
|  |  | GCATATTCCTTGCTGATCTGAAAAGCCAACTC |
|  | His-DDX39B-S137A | AGGAATATGAGCGCTTCGCTAAATACATGCC |
|  |  | CGAAGCGCTCATATTCCTTGCTGATCTGA |
|  | His-DDX39B-L152A | GCTGTTTTTTTTGGTGGTGCGTCTATCAAGAAG |
|  |  | GCACCACCAAAAAAAACAGCAACCTTGACAT |
|  | His-DDX39B-P322A | CCATCCACCGTGGGATGGCCCAGGAGGAGAG |
|  |  | CCATCCCACGGTGGATGGCAATGGCTGG |
|  | His-TRIM28-C65A | TGGAGCTGCTGGAGCACGCCGGCGTGTGCAGAG |
|  |  | GCGTGCTCCAGCAGCTCCAGCGCCTCGGCG |
|  | GST-DDX39B-P322A | CCATCCACCGTGGGATGGCCCAGGAGGAGAG |
|  |  | CCATCCCACGGTGGATGGCAATGGCTGG |
|  | Myc-DDX39B-K95R | CCAAGTCGGGCATGGGAAGGACAGCAGTGTTTG |
|  |  | CTTCCCATGCCCGACTTGGCCTGGCACAG |
|  | Myc-DDX39B-K131R | GGCTTTTCAGATCAGCAGGGAATATGAGCGC |
|  |  | CTGCTGATCTGAAAAGCCAACTCCCGAGTGT |
|  | Myc-DDX39B-K144R | ATACATGCCCAATGTCAGGGTTGCTGTTT |
|  |  | CTGACATTGGGCATGTATTTAGAGAAGCGCT |
|  | Myc-DDX39B-K191R | CAACCTCAAACACATTAGACACTTTATTTTGG |
|  |  | CTAATGTGTTTGAGGTTGAGGCTCTTATTTC |
|  | Myc-DDX39B-K241R | CCGTCCAGTCTGCCGCAGGTTCATGCAAG |
|  |  | CTGCGGCAGACTGGACGGATCTCTTTGCTCA |
|  | Myc-DDX39B-K268R | GCAGCAGTACTACGTGAGACTGAAGGACAAC |
|  |  | CTCACGTAGTACTGCTGCAACCCATGCAG |
|  | Myc-DDX39B-K384R | AGGCCGGTTTGGCACCAGGGGCTTGGCTATC |
|  |  | CTGGTGCCAAACCGGCCTGCTCTGGCCAC |
|  | Myc-DDX39B-K398R | CGATGAGAATGATGCCAGGATCCTCAATGAT |
|  |  | CTGGCATCATTCTCATCGGACACAAATGTG |
|  | pEnter-Flag-His-DDX39B-E197A | AAACACTTTATTTTGGATGCATGTGATAAGATGC |
|  |  | GCATCCAAAATAAAGTGTTTAATGTGTTTGAGG |
|  | pEnter-Flag-His-DDX39B-SAT/AAA | GGTCATGATGTTCGCTGCTGCCTTGAGCAAAGA |
|  |  | CAGCAGCGAACATCATGACCTGCTTCTCGTGG |

**Table S7. List of resources (antibodies, drugs, reagents, plasmids) utilized in this paper.**

| Resources | Name | Manufacturer & Catalog# |
| --- | --- | --- |
| Antibodies | ACTN1 | Sangon Biotech, D221591 |
|  | ACTN4 | Sangon Biotech, D221929 |
|  | DDX39B antibody | Sangon Biotech, BB25AA0014 |
|  | DDX39B antibody | Huabio, ET1706-69 |
|  | DDX39B antibody | Proteintech, 14798-1-AP |
|  | ECAD antibody | Cell Signaling Technology, 14472 |
|  | ECAD antibody | Cell Signaling Technology, 3195 |
|  | EPCAM antibody | Sangon Biotech, D226394 |
|  | Flag-tag antibody | Cell Signaling Technology, 14793 |
|  | GST-tag antibody | Sangon Biotech, D110271 |
|  | Hakai antibody | Proteintech, 21197-1-AP |
|  | HA-tag antibody | Cell Signaling Technology, C29F4 |
|  | His-tag antibody | Huabio, 0812-1 |
|  | IQGAP1 antibody | Sangon Biotech, D163039 |
|  | KRT18 antibody | Sangon Biotech, D120229 |
|  | Lamin B antibody | PTMbio, PTM-5495 |
|  | LAMP-1 antibody | Proteintech, 21997-1-AP |
|  | LMO7 antibody | HuaBio, ER60223 |
|  | Myc tag antibody | Cell Signaling Technology, 2278 |
|  | Myc tag antibody | Huabio, HA601081 |
|  | NCAD antibody | Cell Signaling Technology, 13116 |
|  | pECAD^Y754^ antibody | Affinity, AF7218 |
|  | pSrc^Y416^ antibody | ZENBIO, 530480 |
|  | Pan-pTyr antibody | PTMbio, PTM-702 |
|  | Snail antibody | Cell Signaling Technology, 3879 |
|  | Src antibody | Sangon Biotech, D221267 |
|  | TRIM28 antibody | Huabio, R1210-2 |
|  | TRIM28 antibody | Proteintech, 66630-1-Ig |
|  | Ubiquitin antibody | Huabio, ET1609-2 |
|  | Vimentin antibody | Cell Signaling Technology, 5741 |
|  | β-actin antibody | Sangon Biotech, D110001 |
|  | β-catenin antibody | Huabio, ET1601-5 |
|  | Anti-HRP mouse polyclonal antibody | Sangon Biotech, D110087 |
|  | Anti-HRP rabbit polyclonal antibody | Sangon Biotech, D110058 |
|  | Anti-rabbit IgG, HRP-linked Antibody | Cell Signaling Technology, 7074 |
|  | Alexa Fluor™ 594 Goat anti-rabbit IgG | Invitrogen, A11037 |
|  | FITC-conjugated Affinipure goat anti-mouse | Proteintech, SA00003-1 |
|  | FITC-conjugated Affinipure goat anti-rabbit | Proteintech, SA00003-2 |
|  | FITC-conjugated Affinipure Donkey anti-Goat IgG (H+L) | Proteintech, SA00003-3 |
|  | Rhodamine (TRITC)–conjugated Goat Anti-Mouse IgG (H+L) | Proteintech, SA00007-1 |
| Drugs | Chloroquine | Selleck, S6999 |
|  | MG132 | Selleck, S2619 |
|  | Cycloheximide | Selleck, 66-81-9 |
|  | EGF | Peprotech, PHG0311 |
|  | TGF-β | ACROBiosystems, TG1-H4212 |
|  | Src inhibitor 1 | MCE, HY-101053 |
| Reagents | 1640 medium  2×Power tag PCR MasterMix | Gibco, 31800-022  Bioteke, PR1702 |
|  | Acryl/Bis 30% Solution (29:1) | Sangon Biotech, B546017 |
|  | Anti-Flag Magnetic Beads  Anti-HA Magnetic Beads  AxyPrep^TM^ DNA Gel Extraction Kit  AxyPrep^TM^ Plasmid Miniprep Kit | Sigma, M8823  MCE, HY-K0201  Axygen, AP-GX-250  Axygen, AP-MN-P-250 |
|  | BCA Protein Assay Kit | Sangon Biotech, C503021 |
|  | DAB Detection Kit | Gene Tech, GK600510 |
|  | DNA marker | TransGen Biotech, BM111-01 |
|  | Dpn1 | ThermoFisher, ER1701 |
|  | Dual-Luciferase® Reporter Assay System  D-Luciferin potassium  EasyPure PCR Purification Kit  EndoFree Maxi Plasmid kit  Fast Mutagenesis System  Feto SDS-PAGE staining buffer | Promega, E1960  Meilunstar^®^, MB1834  TransGen Biotech, EP101-02  Tiangen, DP117  TransGen Biotech, FM111-01  Affinibody life science AG, 18.001.10 |
|  | Flag peptides | Sigma, F4799 |
|  | GST 4FF (Pre-Packed Gravity Column) | Sangon Biotech, C600911 |
|  | GST Agarose | Sangon Biotech, C600031 |
|  | GST elution buffer | Sangon Biotech, E675011 |
|  | GST wash buffer | Sangon Biotech, C600326 |
|  | HA peptide | MCE, HY-P0239 |
|  | Hematoxylin and Eosin Staining Kit | Beyotime, C0105M |
|  | HiScript^®^Ⅱ Q Select RT SuperMix for qPCR | Vazyme, R232-01 |
|  | Immobilon ECL Ultra Western HRP Substrate | Merk Millipore, WBULS0100 |
|  | Lipo3000 transfection regent  Lysosome Extraction kit | Invitrogen, L3000-015  Bestbio, BB-3603 |
|  | Martrigel | Corning, 356234 |
|  | MEM medium | Gibco, 12561-056 |
|  | Nuclear and Cytoplasmic Extraction Kit | Cwbio, CW0199 |
|  | Ni-NTA Agarose | Invitrigen, R90101 |
|  | Ni-NTA elution buffer | Sangon Biotech, C600304 |
|  | Ni-NTA wash buffer | Sangon Biotech, C600303 |
|  | Phosphatase Inhibitor | ThermoFisher, 78428 |
|  | Protein A/G Magnetic Beads  Protein inhibitor | MCE，HY-K0202  ThermoFisher, 78430 |
|  | Protein marker  Purified UBE1 protein  Purified UBE2D1 protein | ThermoFisher, 26620  Proteintech, Ag8920  Proteintech, Ag1932 |
|  | Immobilon^®^-PVDF Membrane  Rhodamine-Phalloidin | Merk Millipore, IPVH00010  US Everbright^®^ Inc, YP0063 |
|  | RNAiso plus | Tarkara, 9109 |
|  | Sensor Chip CM5 | Cytiva, BR100012 |
|  | SYBR Green qPCR Master Mix | Bimake, B21203 |
|  | T4 DNA ligase | TransGen Biotech, FL101-02 |
|  | TransStart FastPfu DNA Polymerase | TransGen Biotech, AP221-12 |
|  | Anti-Fade Mounting Medium | Sangon Biotech, E675011 |
|  | Trypsin | Sigma, T4799 |
| Plasmids | pcDNA3.1-HA tag vector | Addgene, 128034 |
|  | pET28a-His tag vector | EMD Biosciences, 69864-3 |
|  | pGEX-5X-3-GST tag vector | kindly provided by Qunyin Lei, N/A |
|  | pRK7-Myc tag vector | Addgene, 19400 |
|  | pEnter-Flag-His-vector  pEnter-Flag-His-DDX39B | Vigenebio, N/A  Vigenebio, CH821820 |
|  | pEnter-Flag-His-TRIM28 | Vigenebio, CH831297 |
|  | pCAGGS-TRIM28-HA | kindly provided by Xiaofeng Guo, N/A |
|  | pCAGGS-TRIM28-ΔRING-HA | kindly provided by Xiaofeng Guo, N/A |
|  | Flag-ub | kindly provided by Hongbo Hu, N/A |
|  | HA-ub | kindly provided by Hongbo Hu, N/A |
|  | HA-ub-K6 | Miaoling biology, P31806 |
|  | HA-ub-K11 | Miaoling biology, P31913 |
|  | HA-ub-K27 | Miaoling biology, P31987 |
|  | HA-ub-K29 | Miaoling biology, P31814 |
|  | HA-ub-K33 | Miaoling biology, P31798 |
|  | HA-ub-K48 | kindly provided by Hongbo Hu, N/A |
|  | HA-ub-K63 | kindly provided by Hongbo Hu, N/A |
|  | pcDNA3.1- HA-Hakai | kindly provided by Yasuyuki Fujita, N/A |
|  | pRL-TK | kindly provided by Jinyi Liu, N/A |
|  | TOP Flash reporter | kindly provided by Jinyi Liu, N/A |
|  | FOP Flash reporter | kindly provided by Jinyi Liu, N/A |
|  | pLV-Neo-CMV-luciferase vector | Provided by VectorBuilder |
|  | pLV[Exp]-Puro-EF1A>Flag-DDX39B (wild type) | Provided by VectorBuilder |
|  | pLV[Exp]-Puro-EF1A>Flag-DDX39B (P322A) | Provided by VectorBuilder |
|  | pLV[Exp]-Puro-EF1A>Flag-DDX39B (3KR) | Provided by VectorBuilder |
|  | pLV[Exp]-EGFP:T2A:Hygro-CMV>HA-CDH1 | Provided by VectorBuilder |
|  | pDEST40-2XHA-wtSrc | Addgene, 140294 |
|  | pGEX-5X-3-GST-DDX39B | This paper, N/A |
|  | pGEX-5X-3-GST-DDX39B-P322A | This paper, N/A |
|  | pGEX-5X-3-GST-DDX39B-3KR | This paper, N/A |
|  | pGEX-5X-3-GST-TRIM28 | This paper, N/A |
|  | pGEX-5X-3-GST-ECD | This paper, N/A |
|  | pEnter-Flag-His-DDX39B-E197A | This paper, N/A |
|  | pEnter-Flag-His-DDX39B-SAT/AAA | This paper, N/A |
|  | pET28a-His-DDX39B | This paper, N/A |
|  | pET28a-His-DDX39B-F127A | This paper, N/A |
|  | pET28a-His-DDX39B-E134A | This paper, N/A |
|  | pET28a-His-DDX39B-S137A | This paper, N/A |
|  | pET28a-His-DDX39B-L152A | This paper, N/A |
|  | pET28a-His-DDX39B-P322A | This paper, N/A |
|  | pET28a-His-TRIM28 | This paper, N/A |
|  | pET28a-His-TRIM28-C65A | This paper, N/A |
|  | pRK7-Myc-DDX39B | This paper, N/A |
|  | pRK7-Myc-DDX39B-K95R | This paper, N/A |
|  | pRK7-Myc-DDX39B-K131R | This paper, N/A |
|  | pRK7-Myc-DDX39B-K144R | This paper, N/A |
|  | pRK7-Myc-DDX39B-K191R | This paper, N/A |
|  | pRK7-Myc-DDX39B-K241R | This paper, N/A |
|  | pRK7-Myc-DDX39B-K268R | This paper, N/A |
|  | pRK7-Myc-DDX39B-K384R | This paper, N/A |
|  | pRK7-Myc-DDX39B-K398R | This paper, N/A |
|  | pRK7-Myc-DDX39B-3KR (K241R, K384R, K398R) | This paper, N/A |
|  | pBiFc-VN173 vector | Addgene, 22010 |
|  | pBiFc-VC155 vector | Addgene, 22011 |
|  | pBiFc-VN173-DDX39B | This paper, N/A |
|  | pBiFc-VC155-TRIM28 | This paper, N/A |
|  | pBiFc-VC155-ECAD | This paper, N/A |
