# Supplementary figures and images for: DDX39B K63-linked ubiquitination mediated by TRIM28 promotes NSCLC metastasis by enhancing ECAD lysosomal degradation
Source: Signal Transduct Target Ther. 2025 Jul 16;10:221. doi: 10.1038/s41392-025-02305-9 (PMC12263876; doi:10.1038/s41392-025-02305-9)

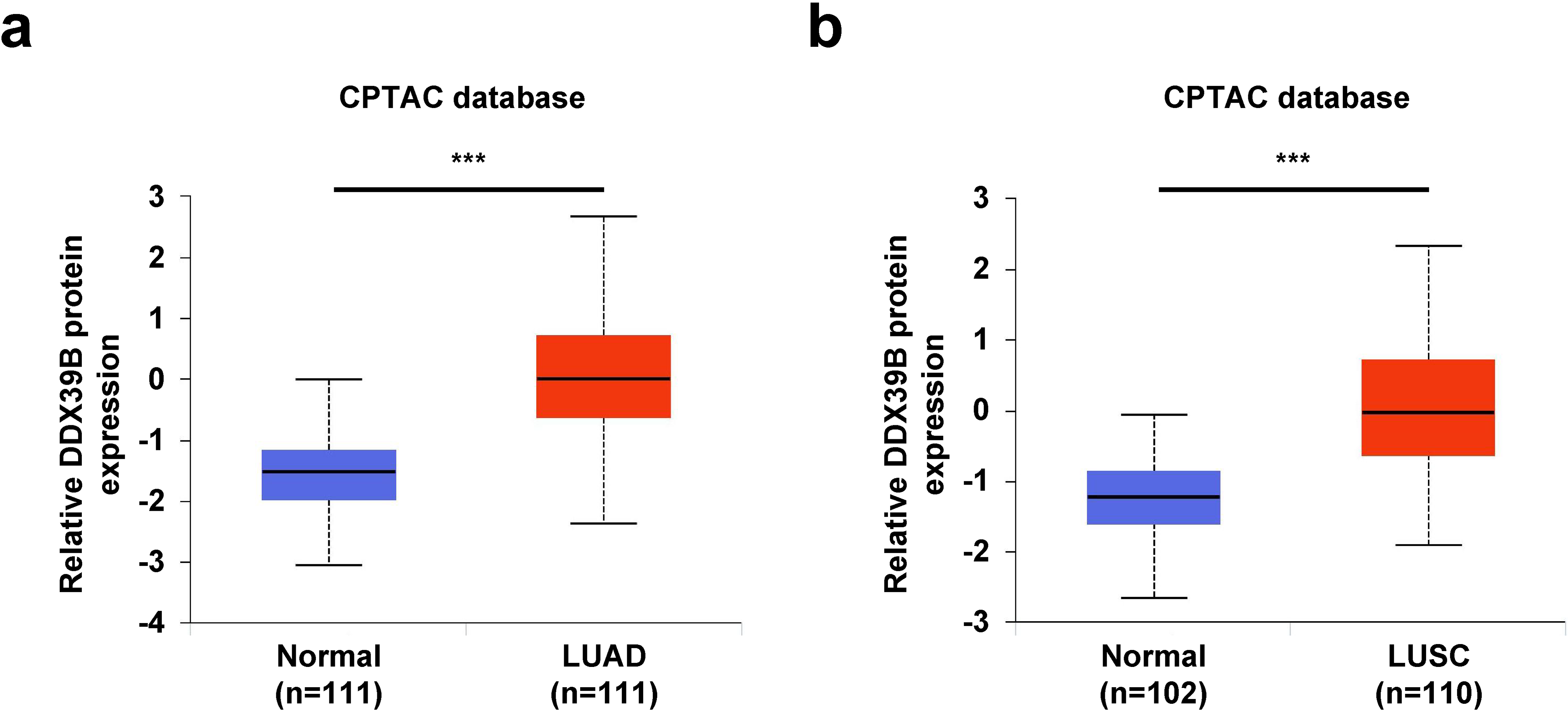

Supplement: Supplementary file 2 — Supplementary Fig. 1 [file 41392_2025_2305_MOESM2_ESM.tif]

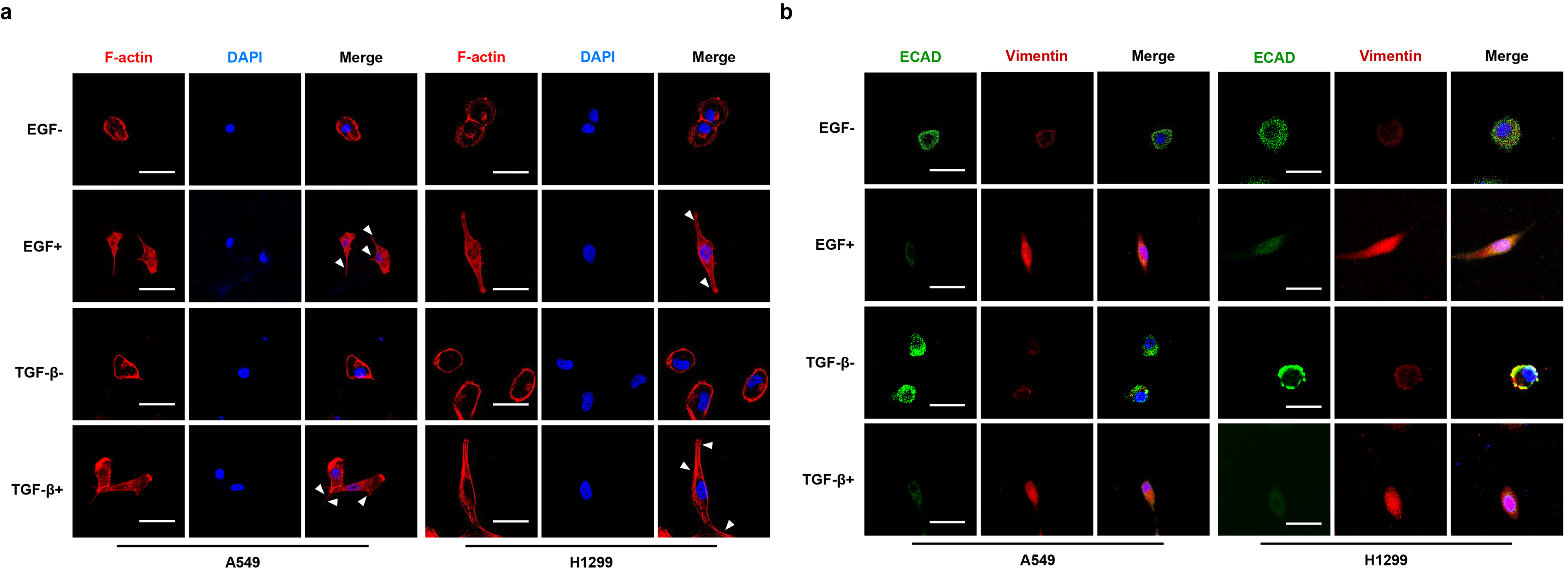

Supplement: Supplementary file 3 — Supplementary Fig. 2 [file 41392_2025_2305_MOESM3_ESM.tif]

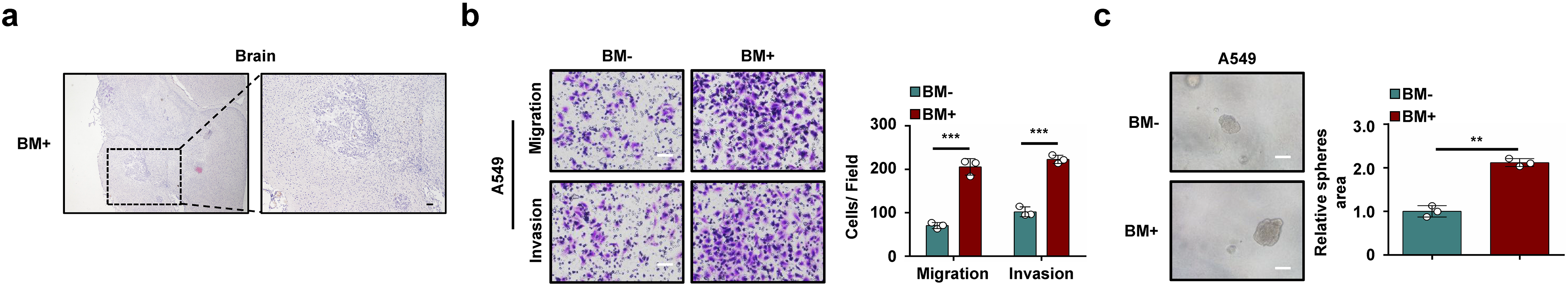

Supplement: Supplementary file 4 — Supplementary Fig. 3 [file 41392_2025_2305_MOESM4_ESM.tif]

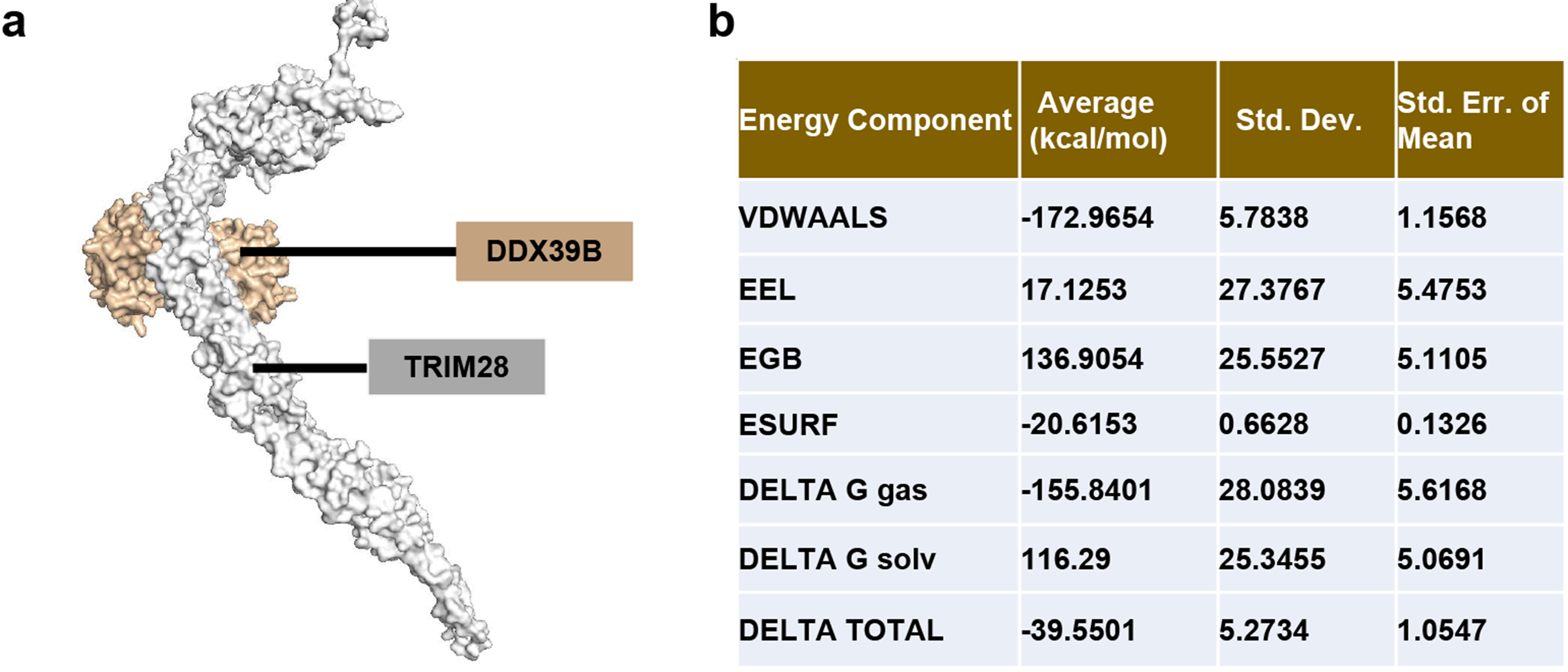

Supplement: Supplementary file 5 — Supplementary Fig. 4 [file 41392_2025_2305_MOESM5_ESM.tif]

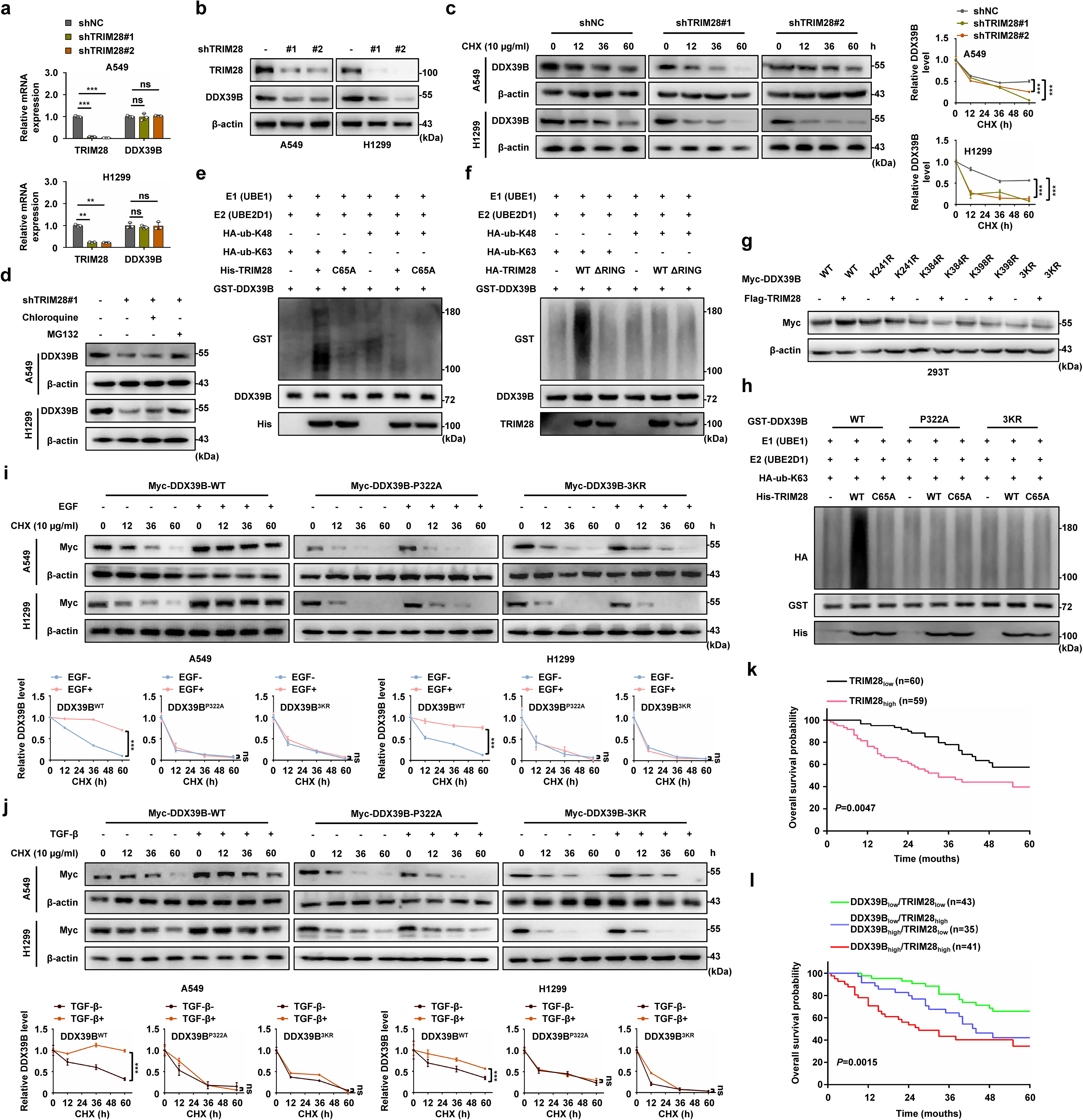

Supplement: Supplementary file 6 — Supplementary Fig. 5 [file 41392_2025_2305_MOESM6_ESM.tif]

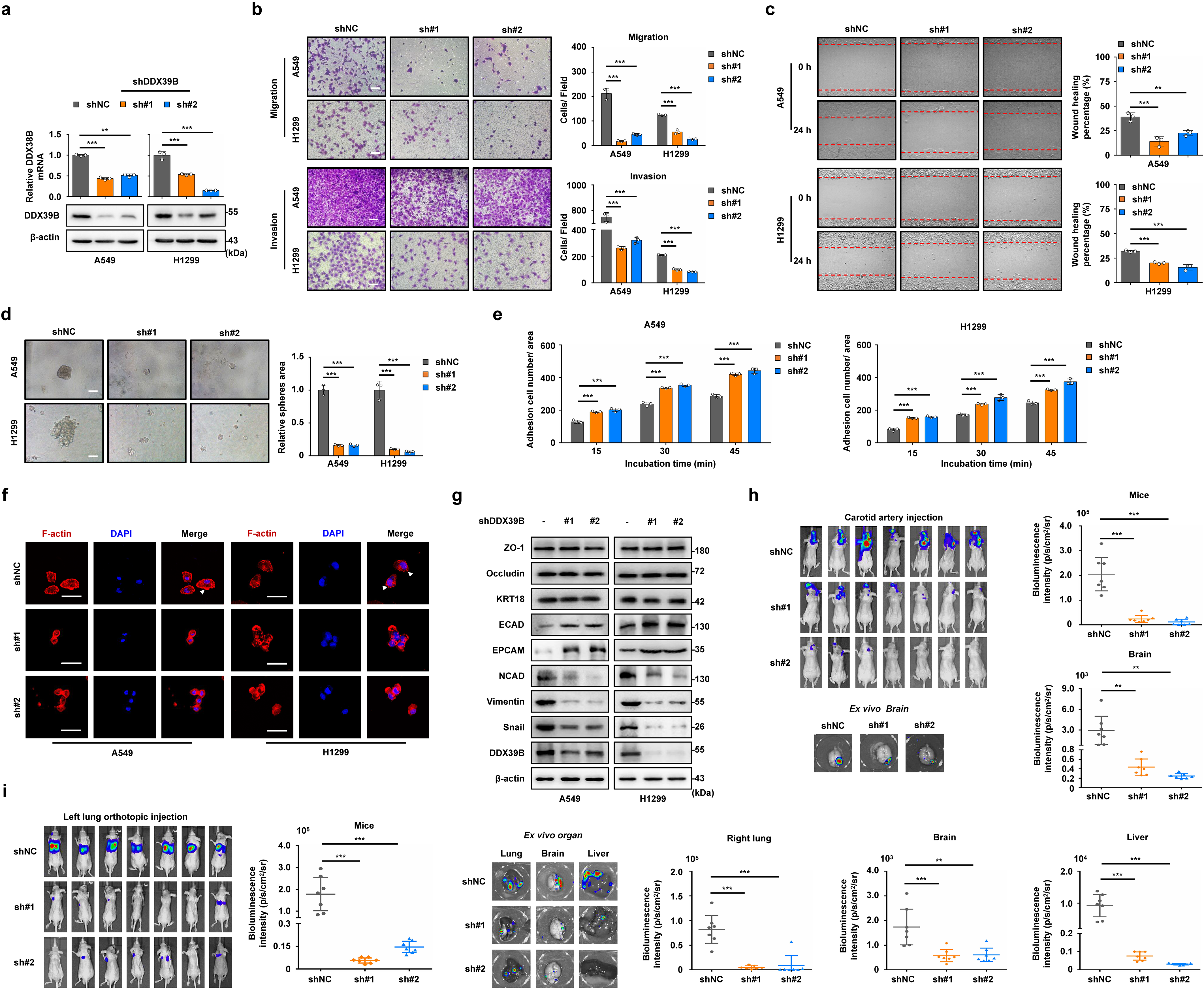

Supplement: Supplementary file 7 — Supplementary Fig. 6 [file 41392_2025_2305_MOESM7_ESM.tif]

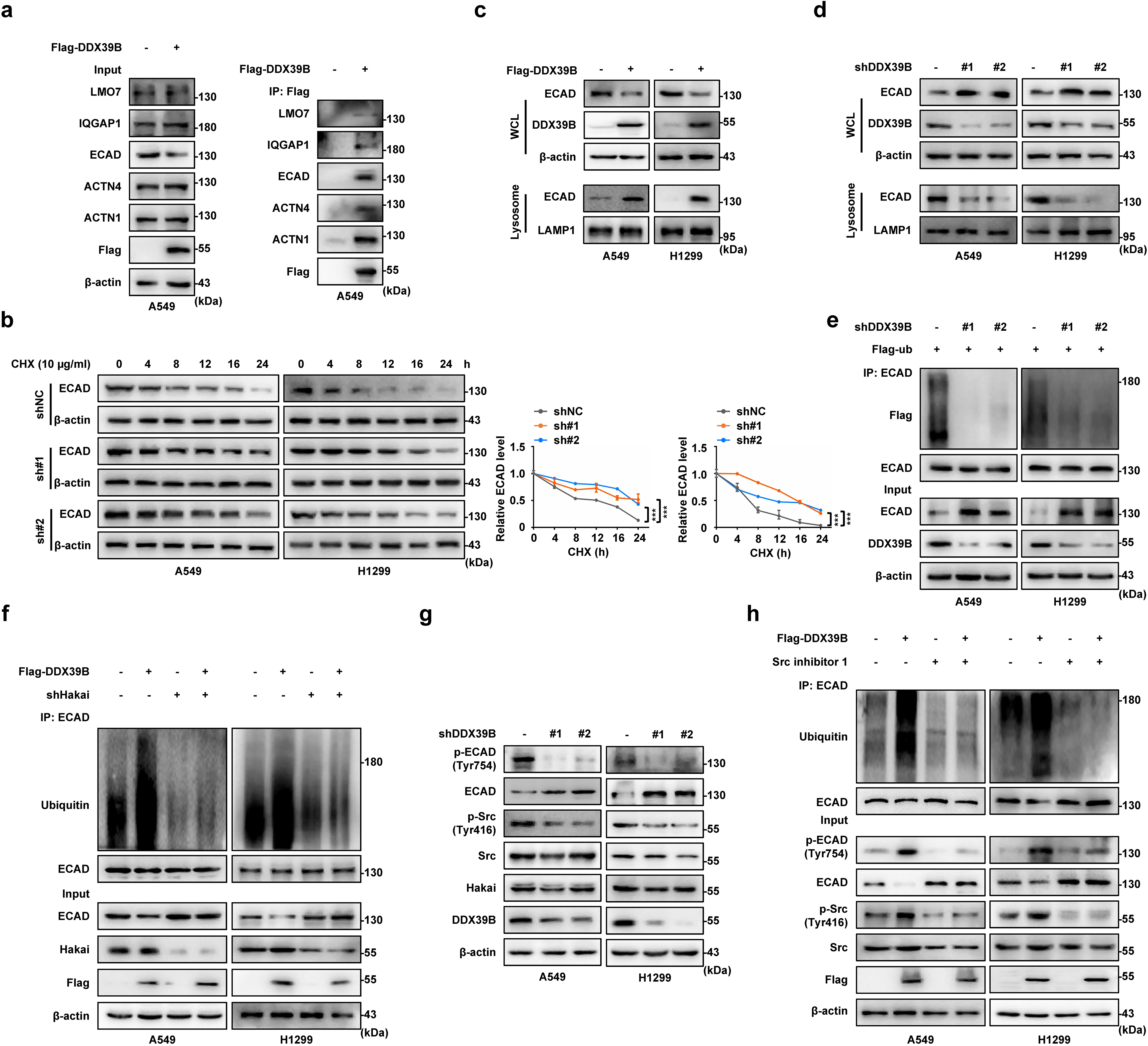

Supplement: Supplementary file 8 — Supplementary Fig. 7 [file 41392_2025_2305_MOESM8_ESM.tif]

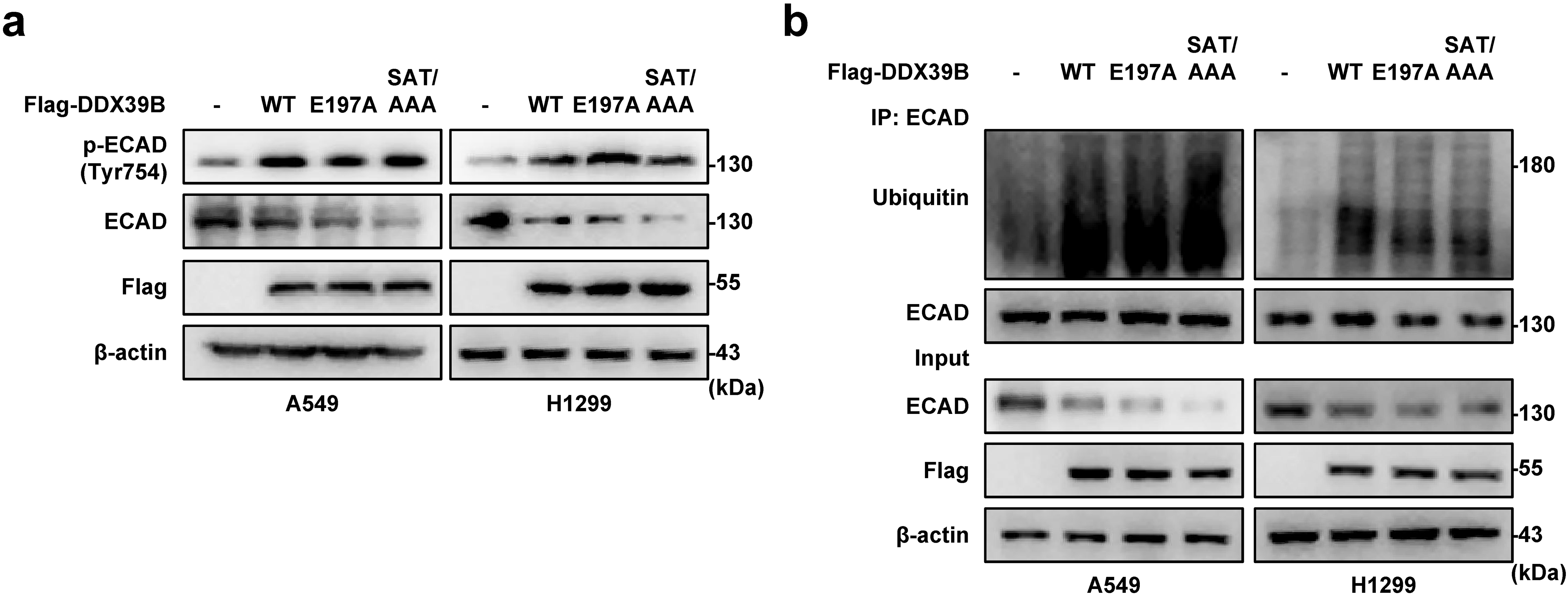

Supplement: Supplementary file 9 — Supplementary Fig. 8 [file 41392_2025_2305_MOESM9_ESM.tif]

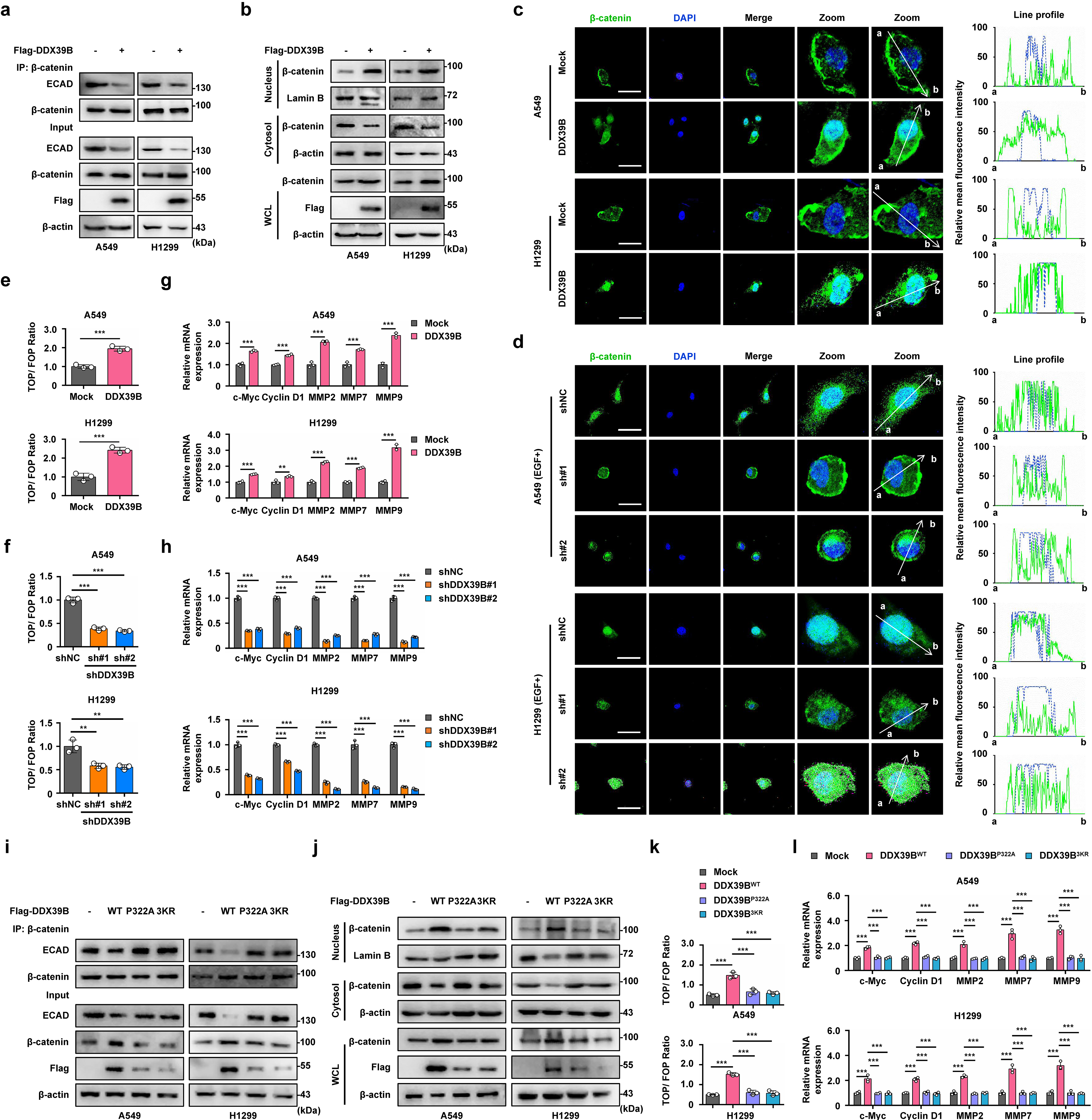

Supplement: Supplementary file 10 — Supplementary Fig. 9 [file 41392_2025_2305_MOESM10_ESM.tif]

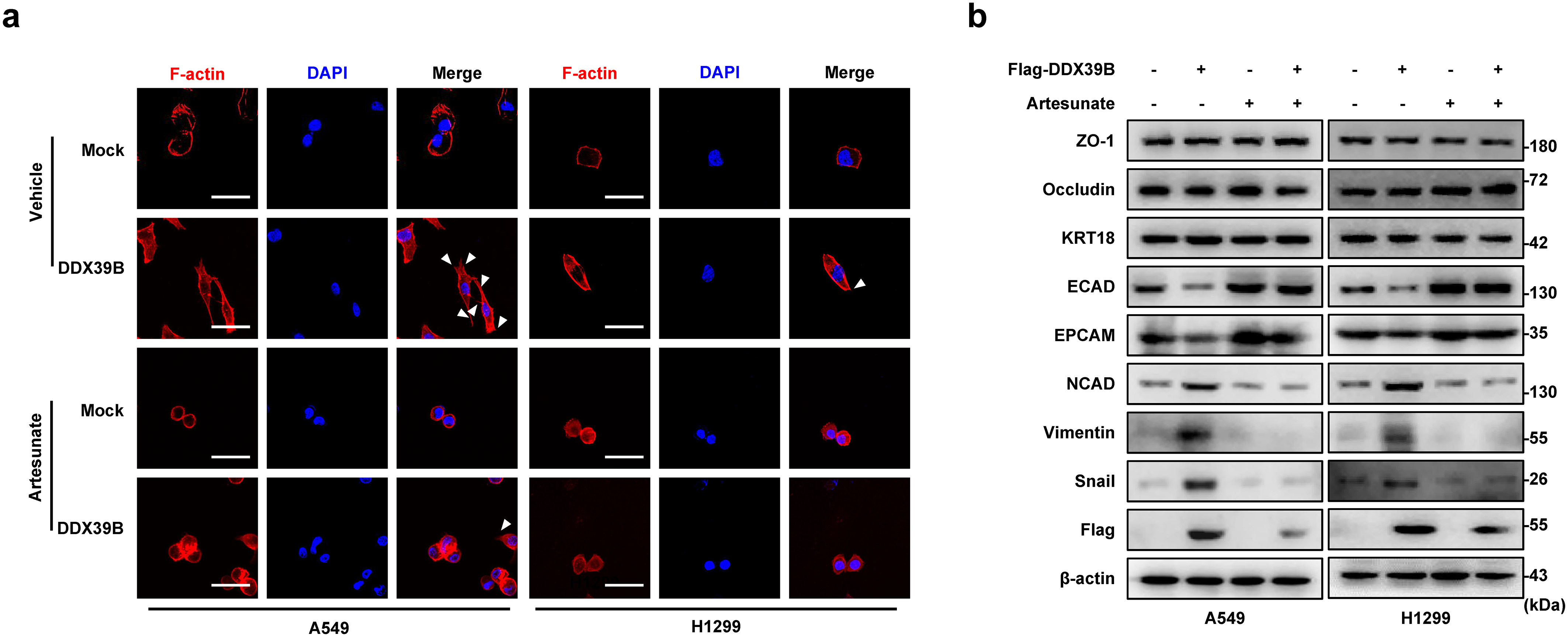

Supplement: Supplementary file 11 — Supplementary Fig. 10 [file 41392_2025_2305_MOESM11_ESM.tif]

Fig. 2

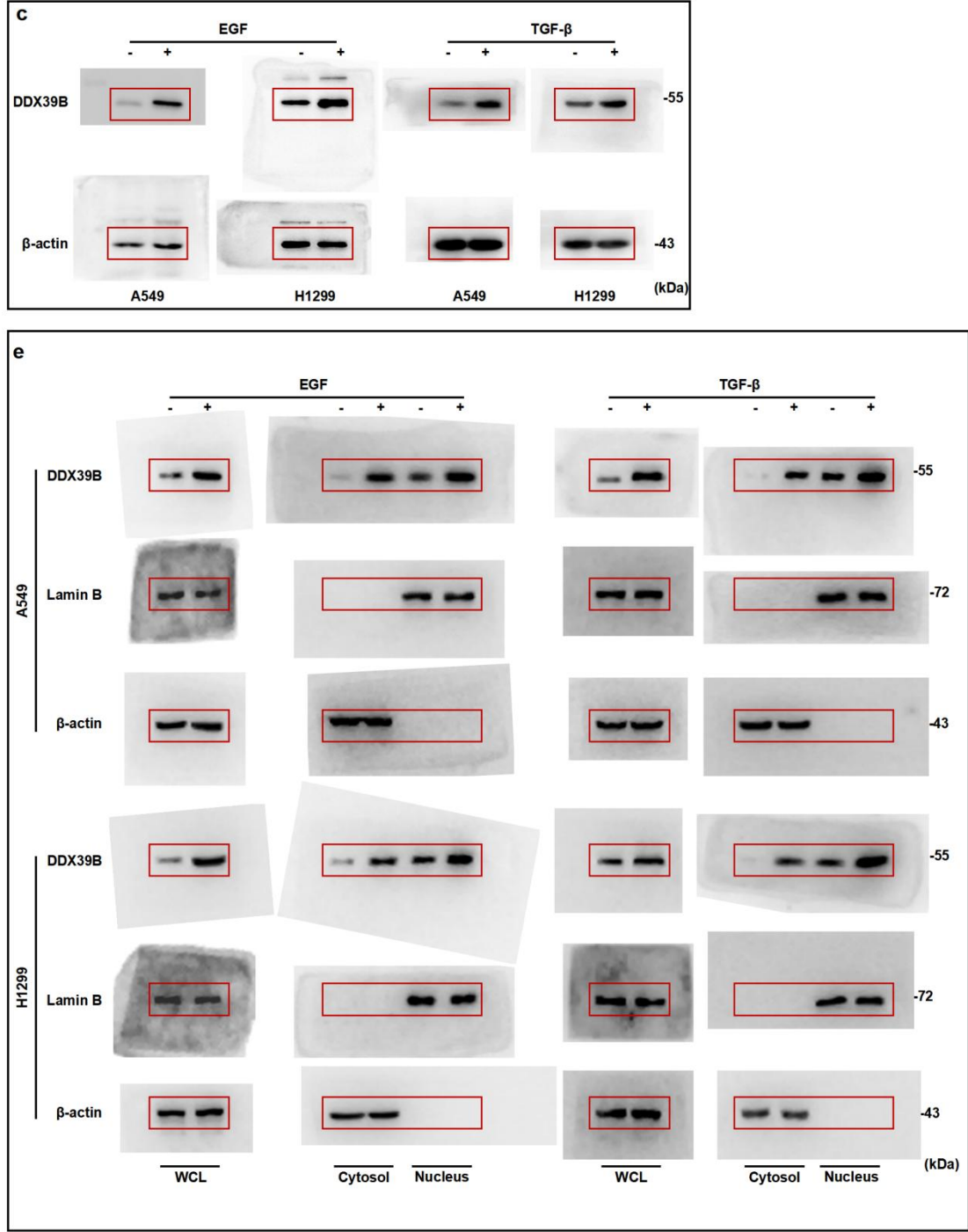

**Fig. 2**

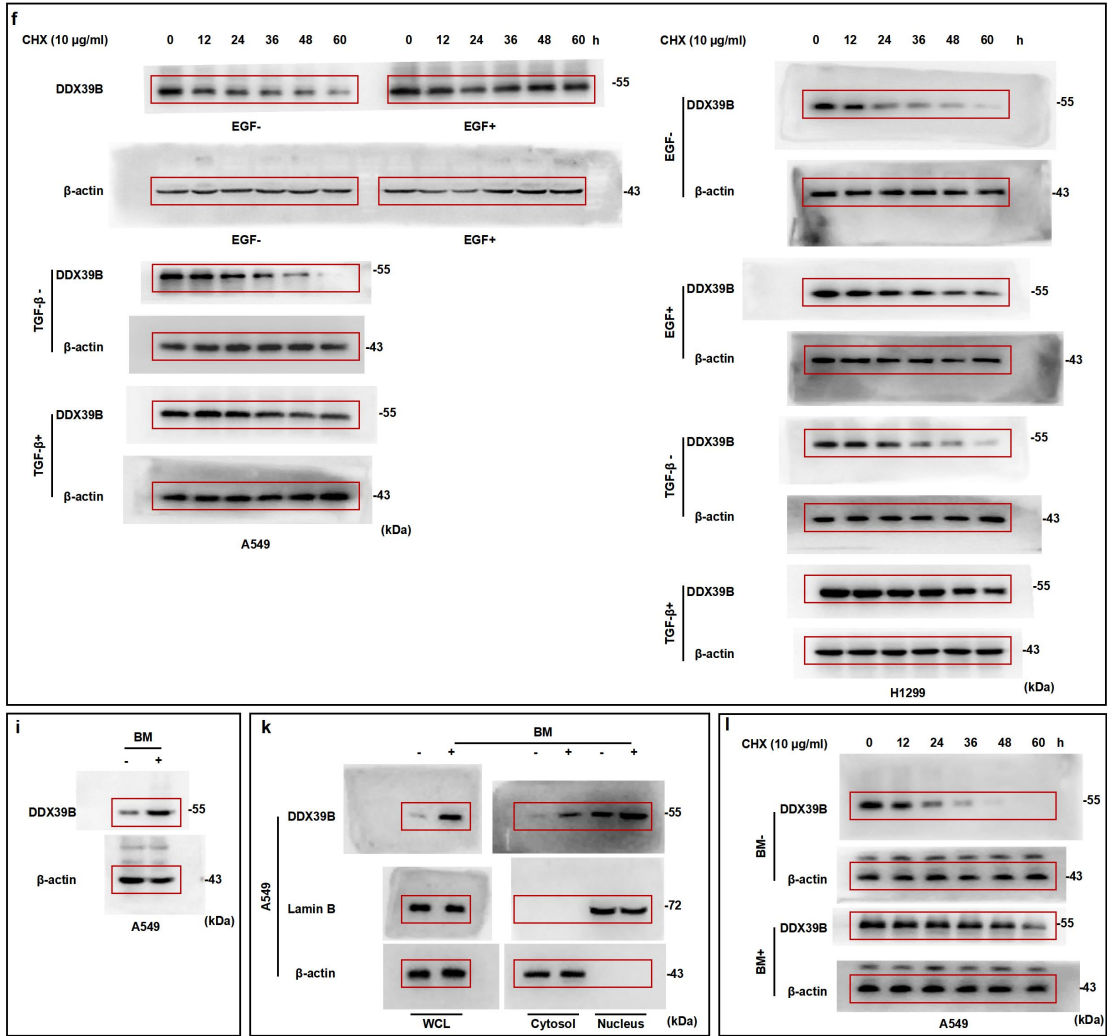

Fig. 3

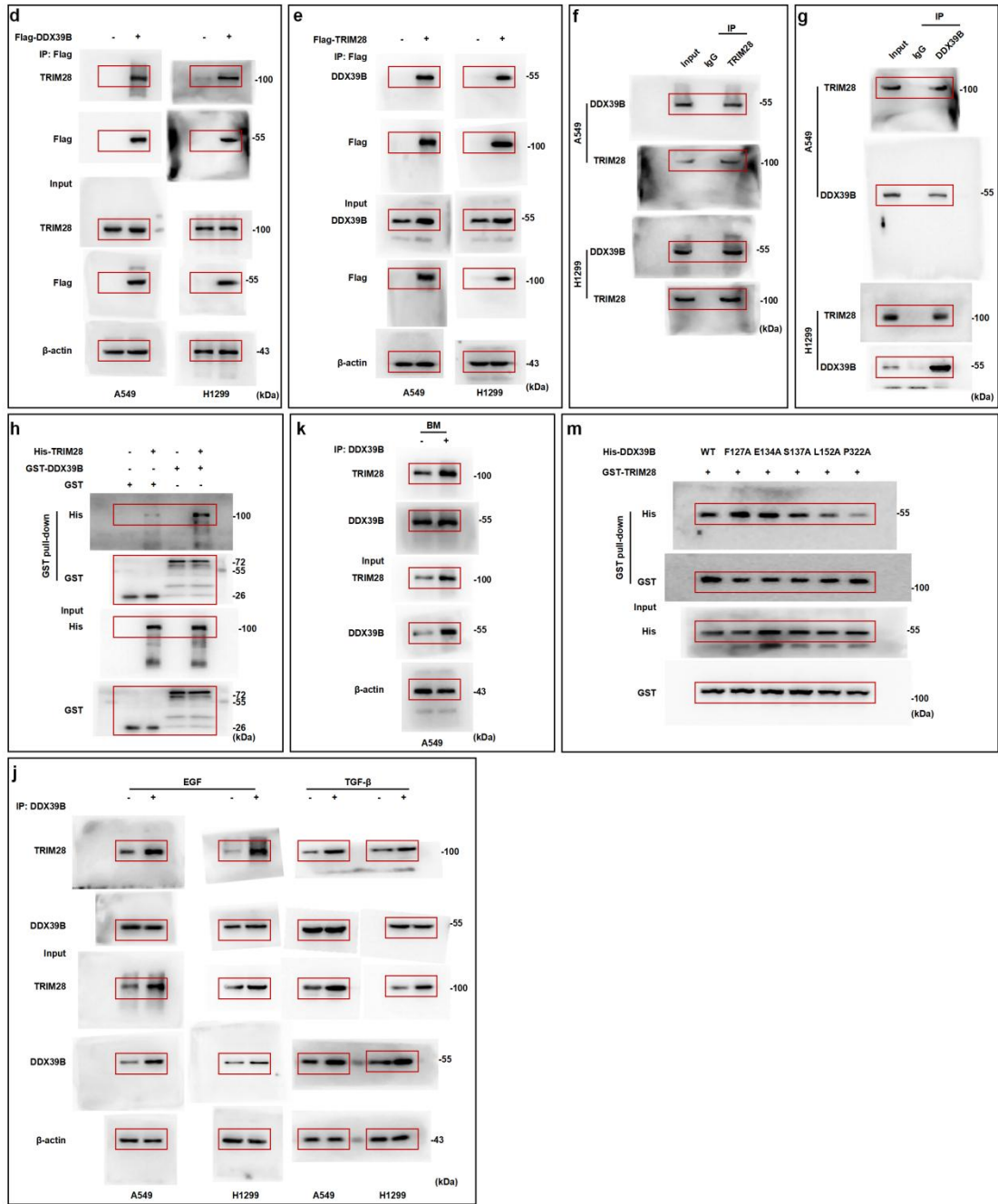

**Fig. 4**

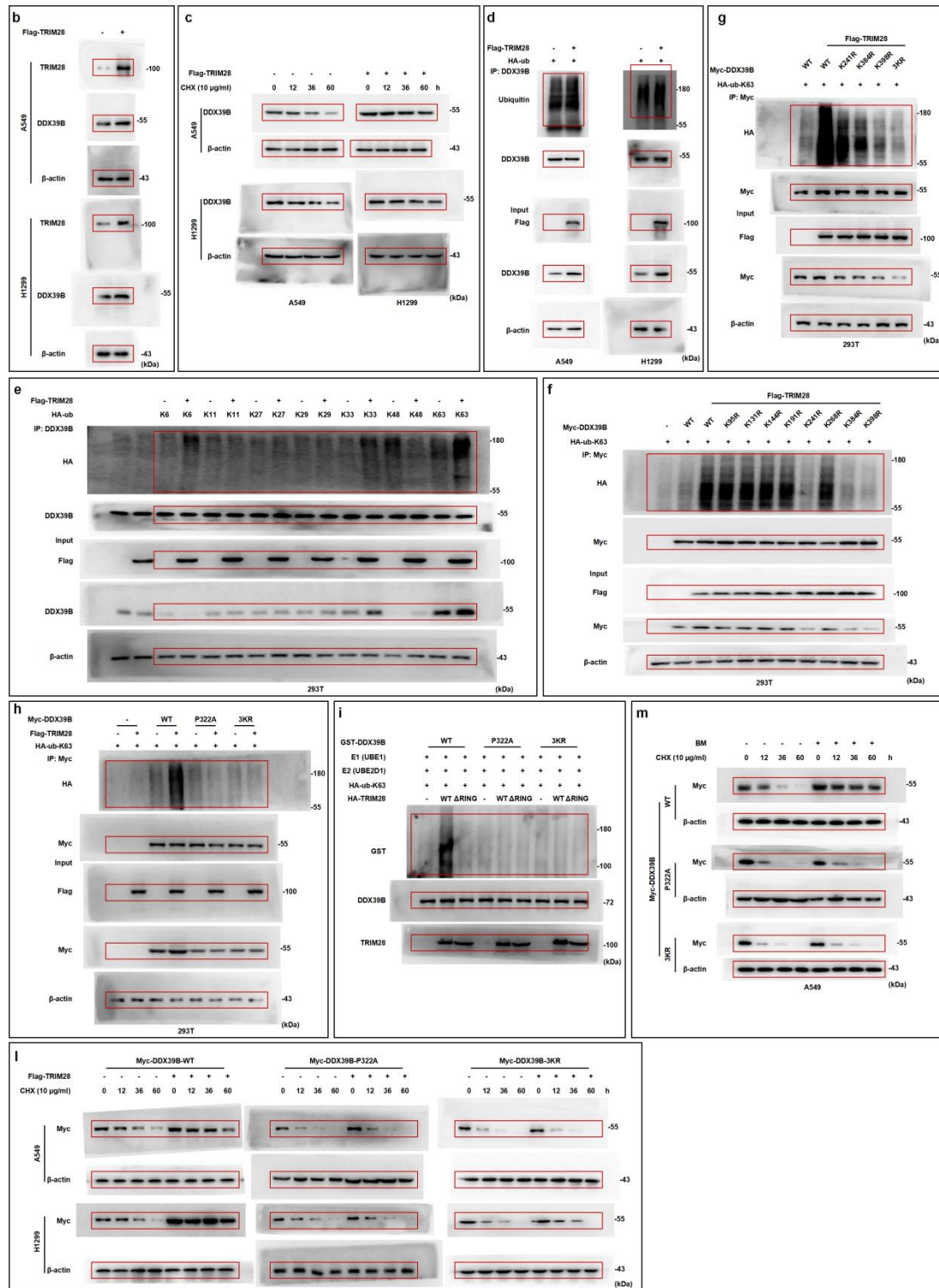

Fig. 5

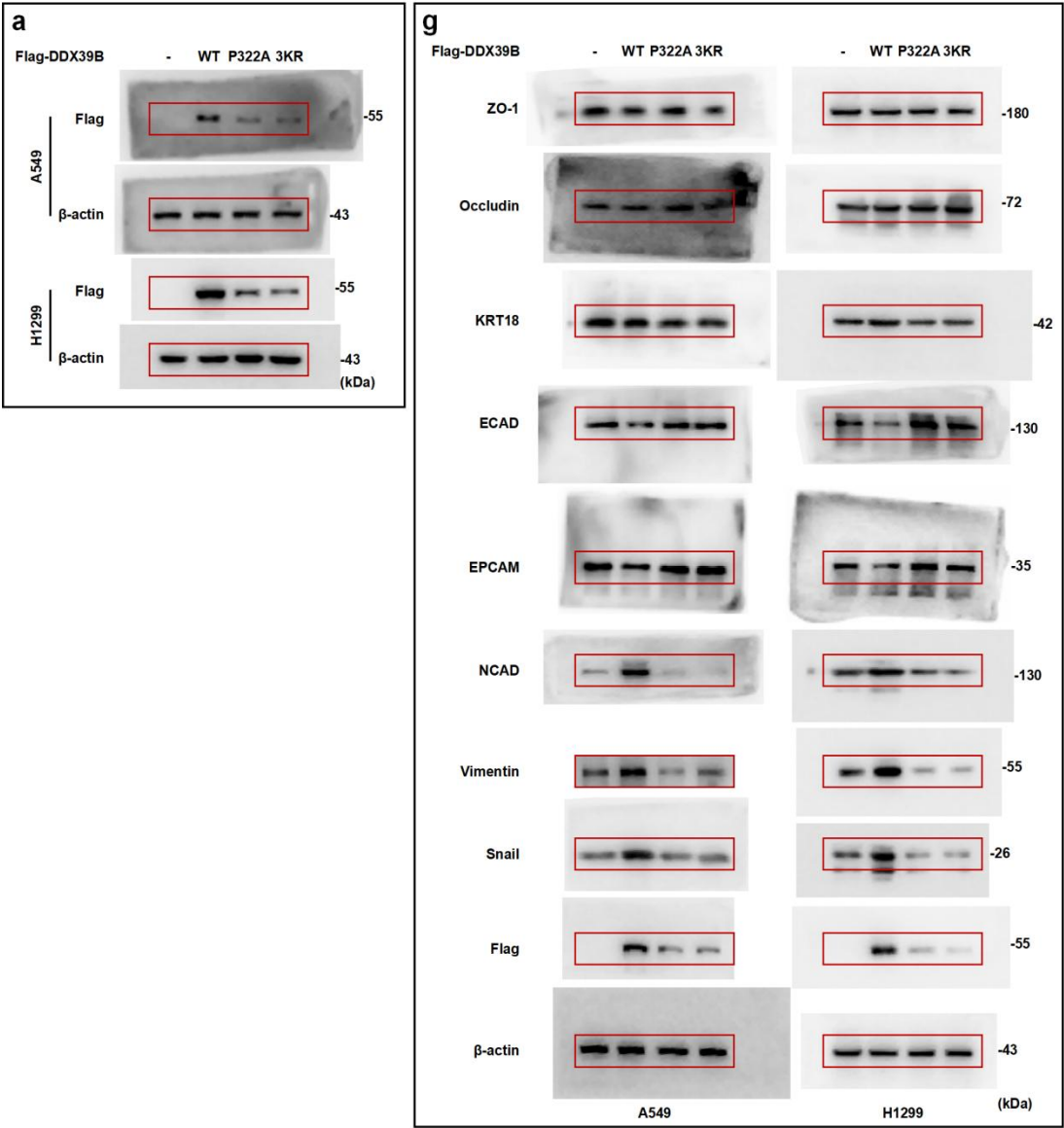

**Fig. 6**

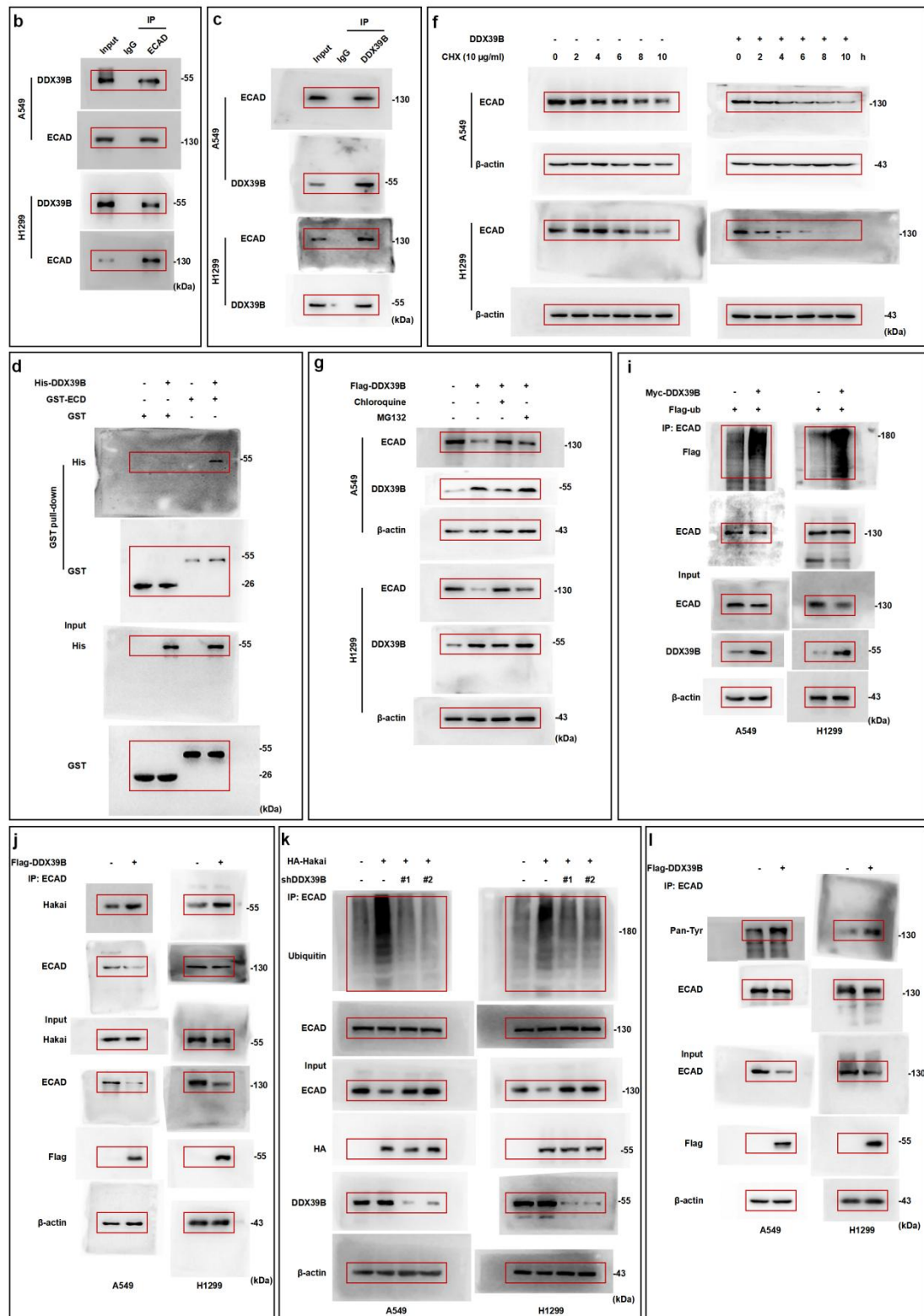

**Fig. 6**

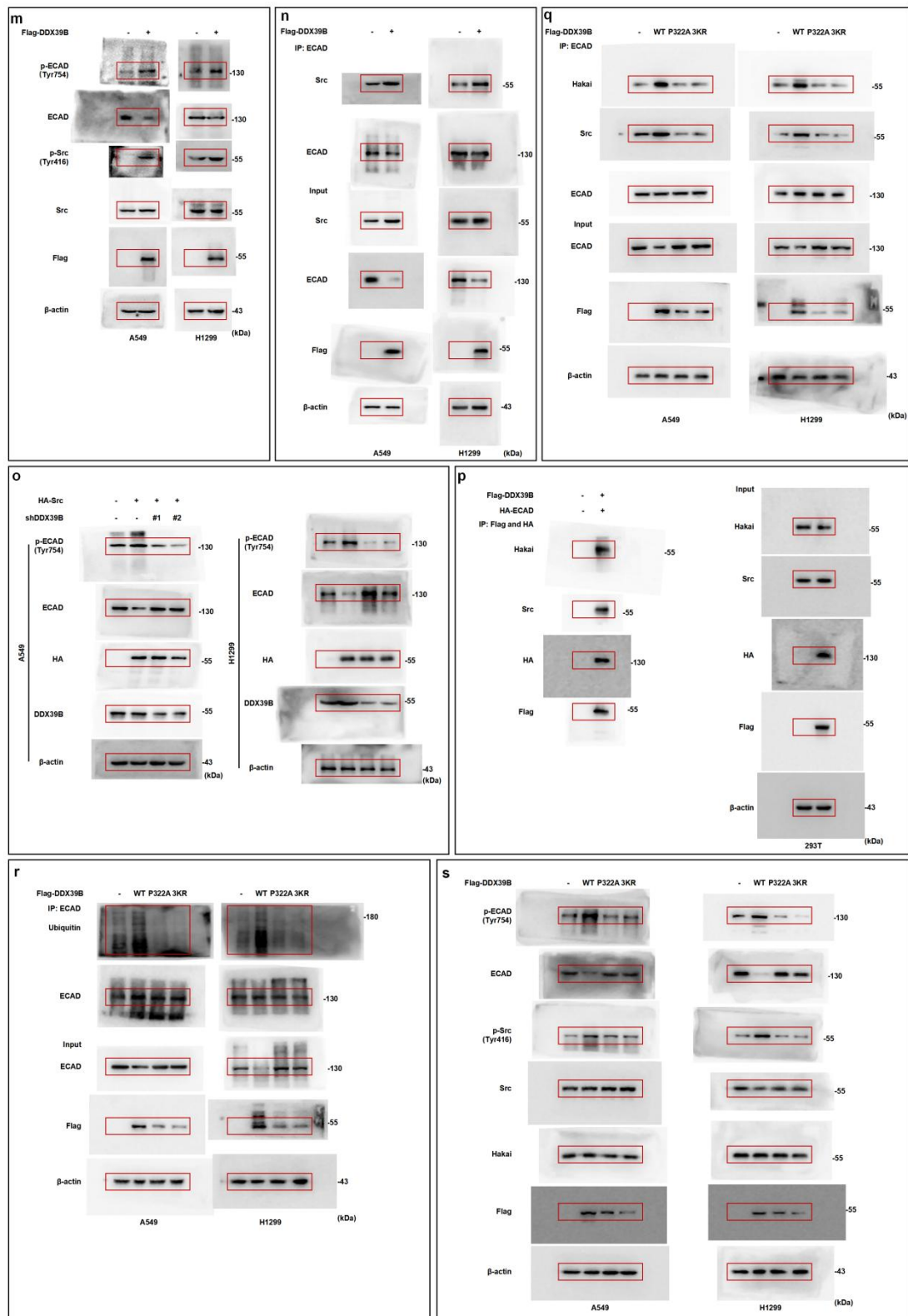

Fig. 7

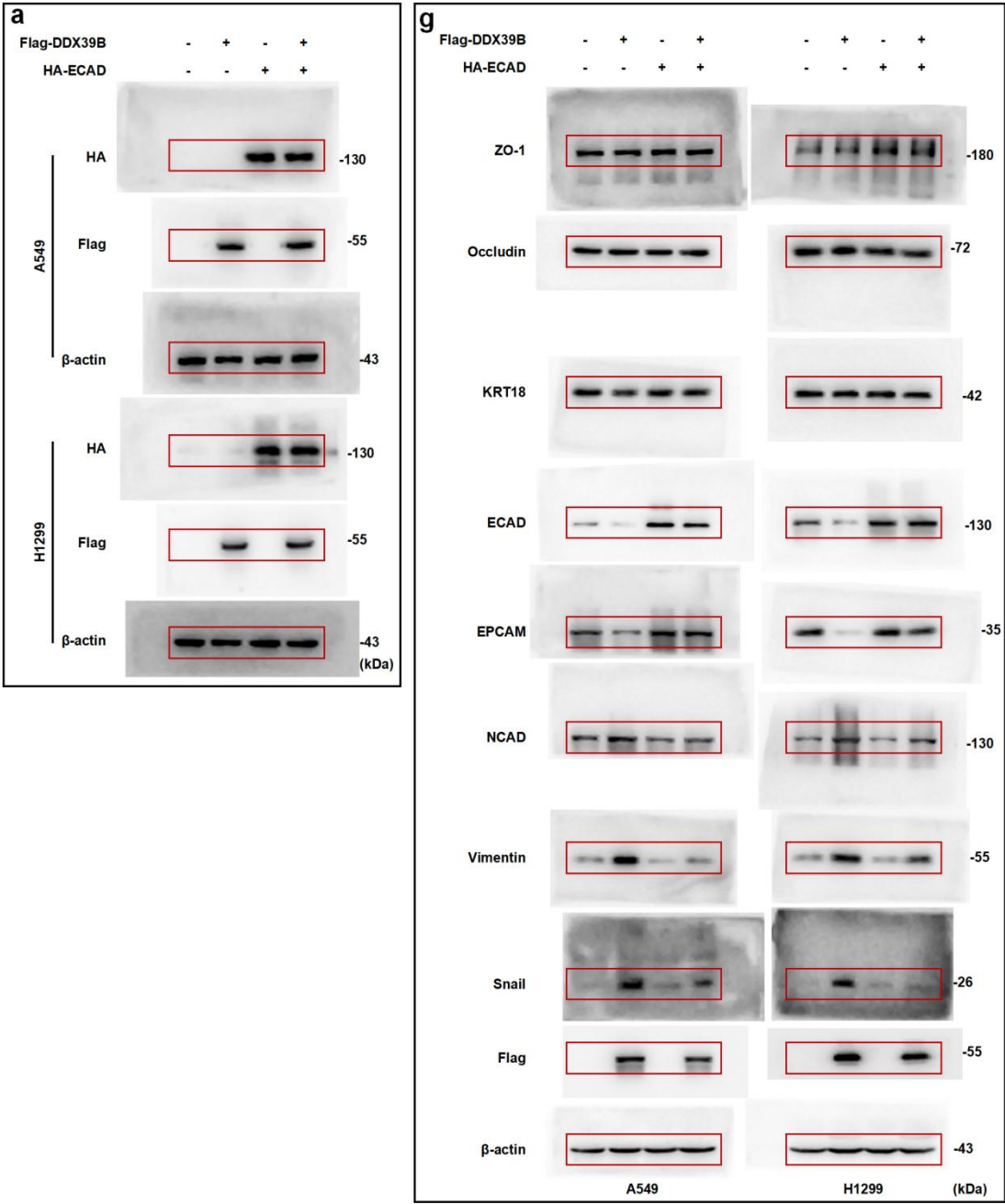

**Fig. 8**

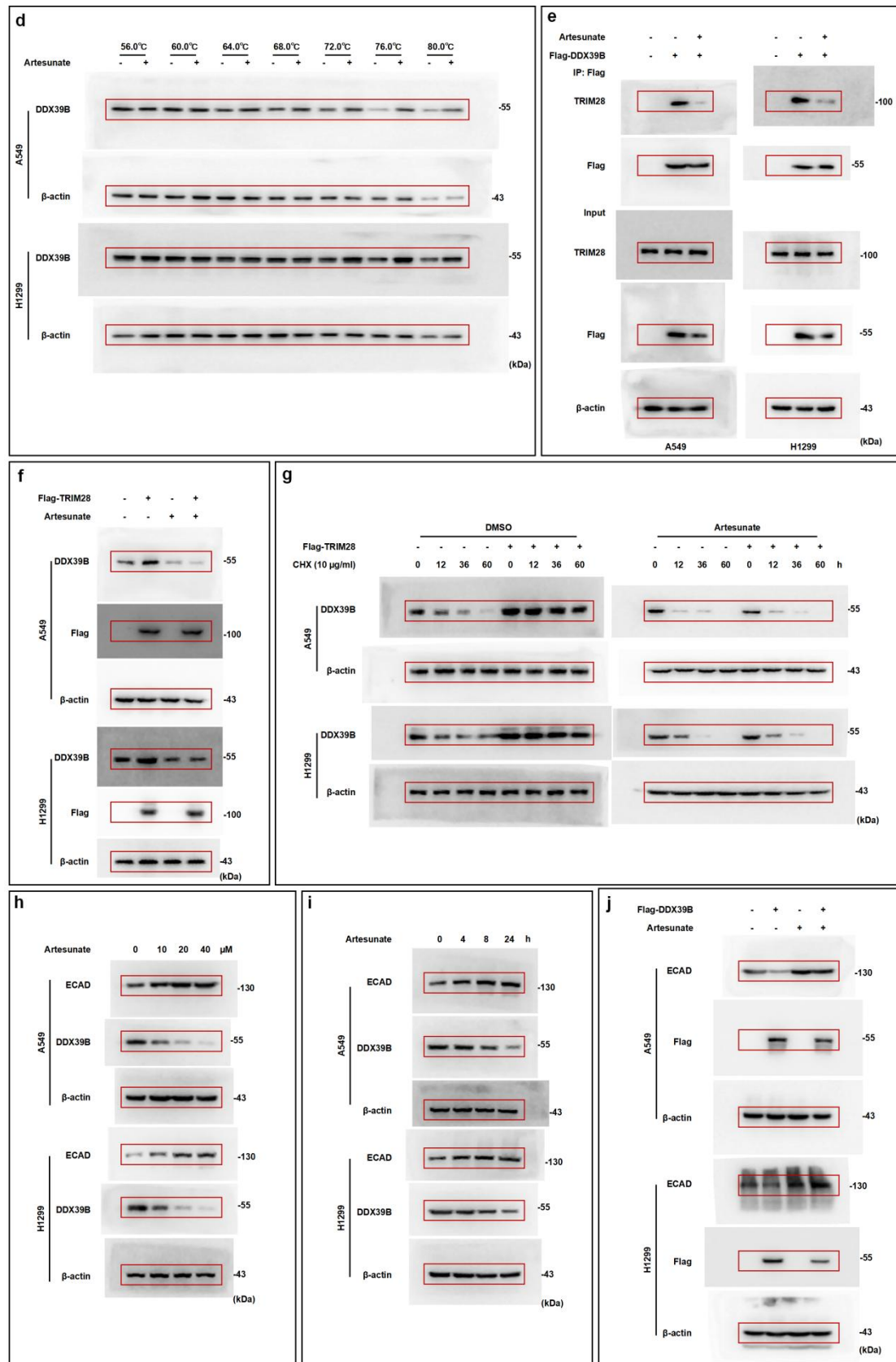

Fig. S5

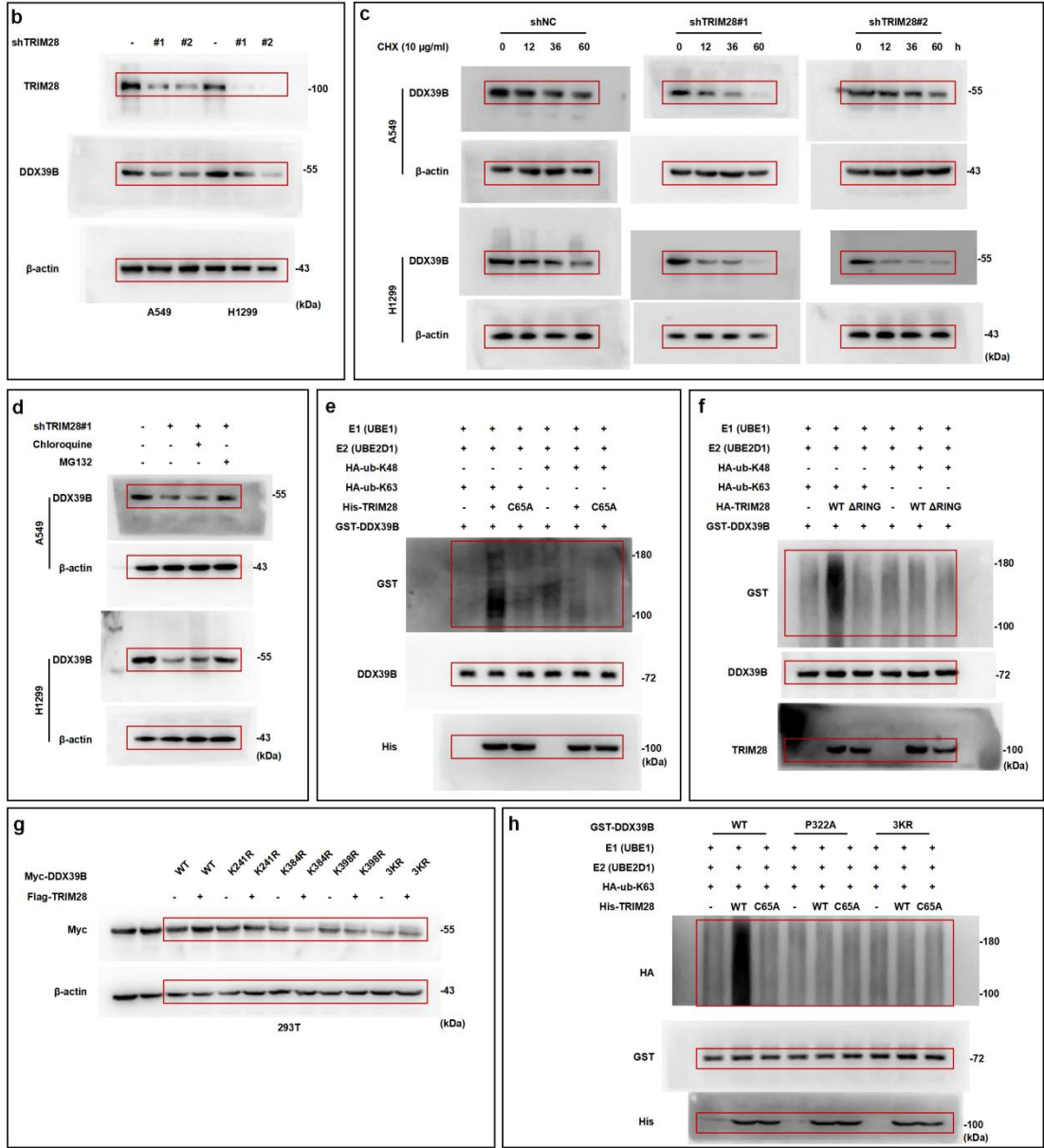

Fig. S5

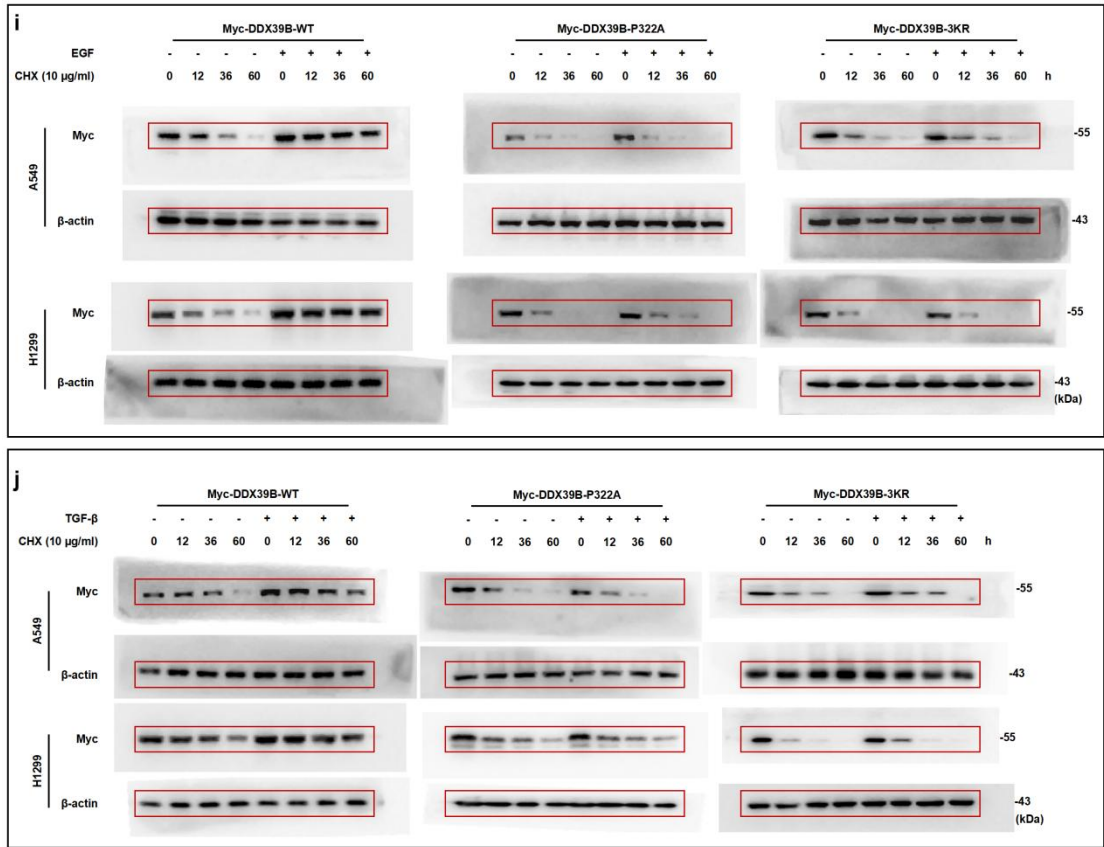

Fig. S6

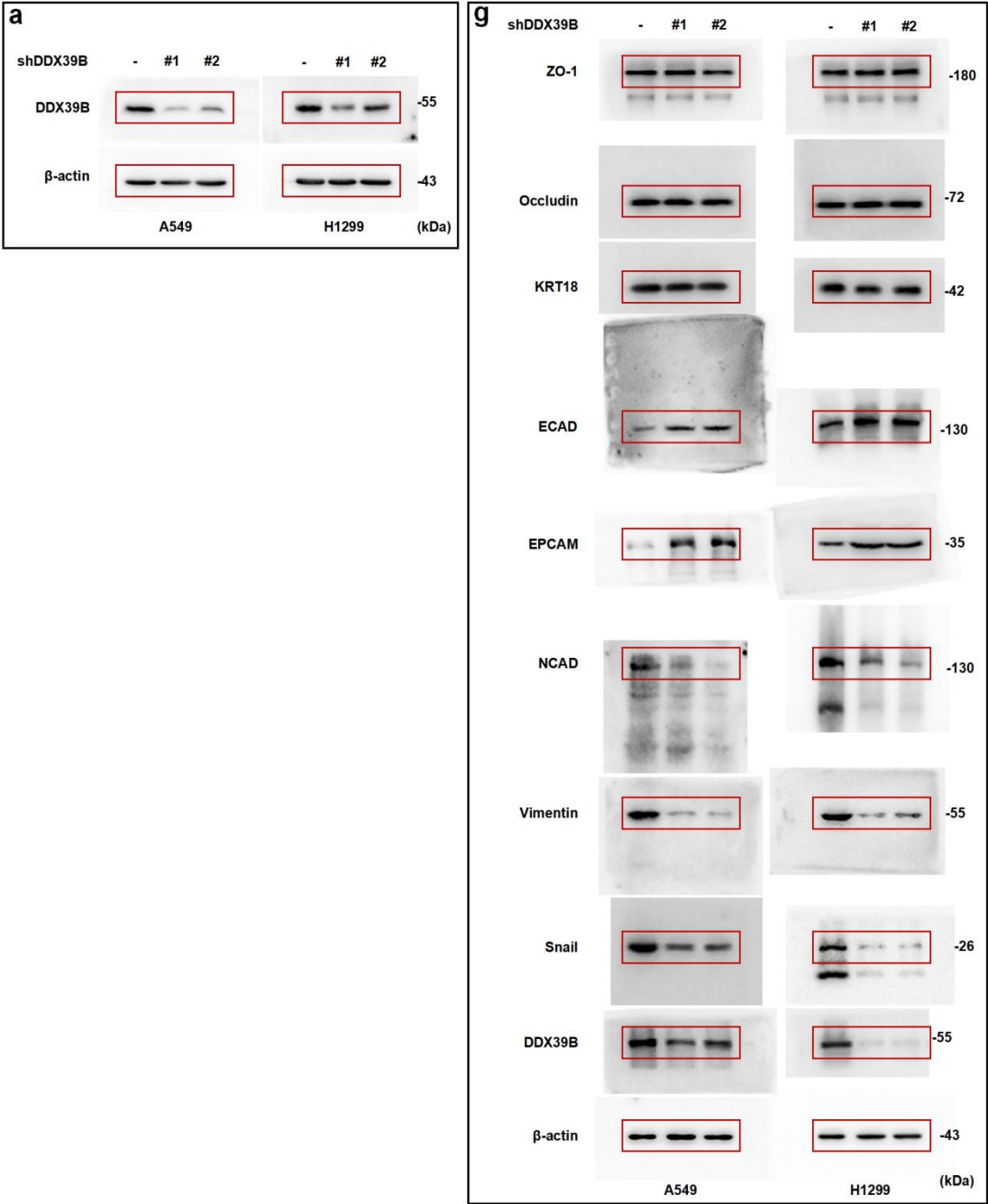

**Fig. S7**

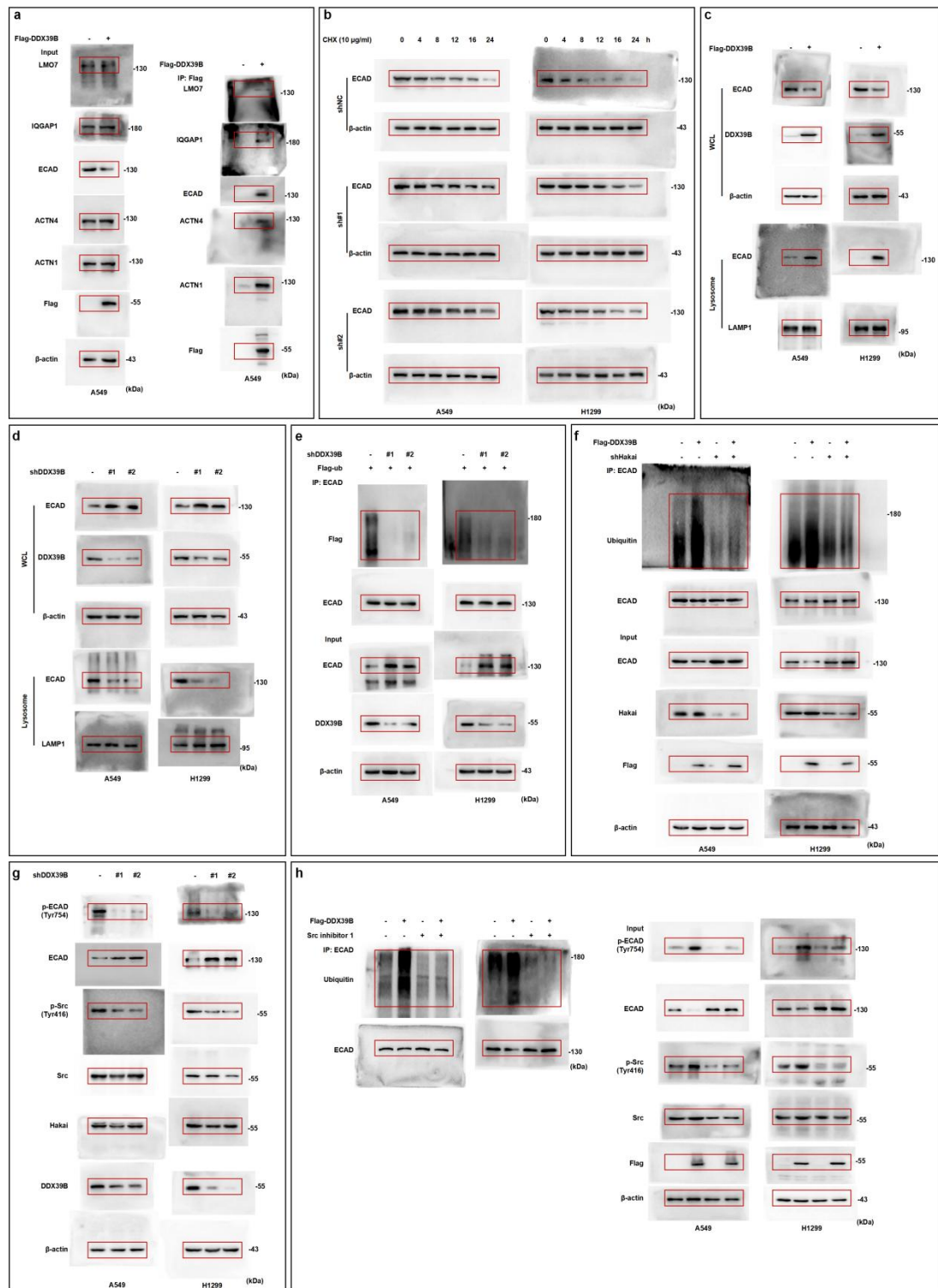

**Fig. S8**

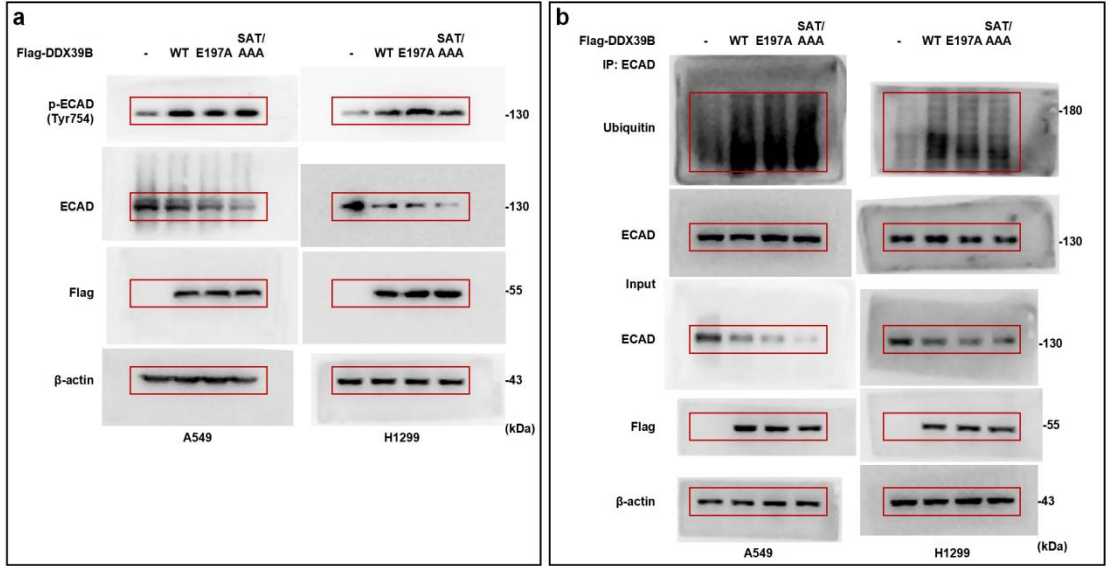

**Fig. S9**

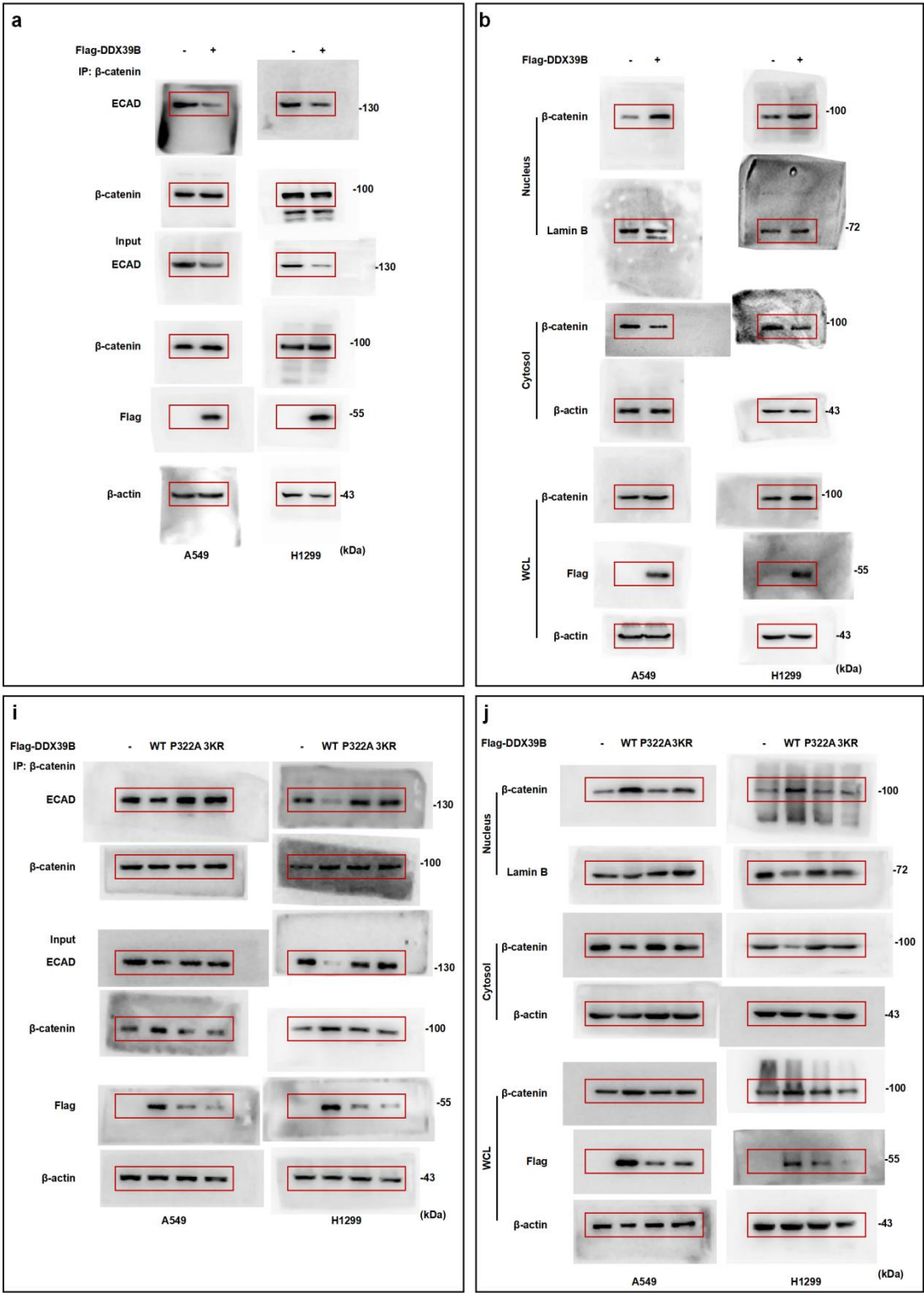

**Fig. S10**

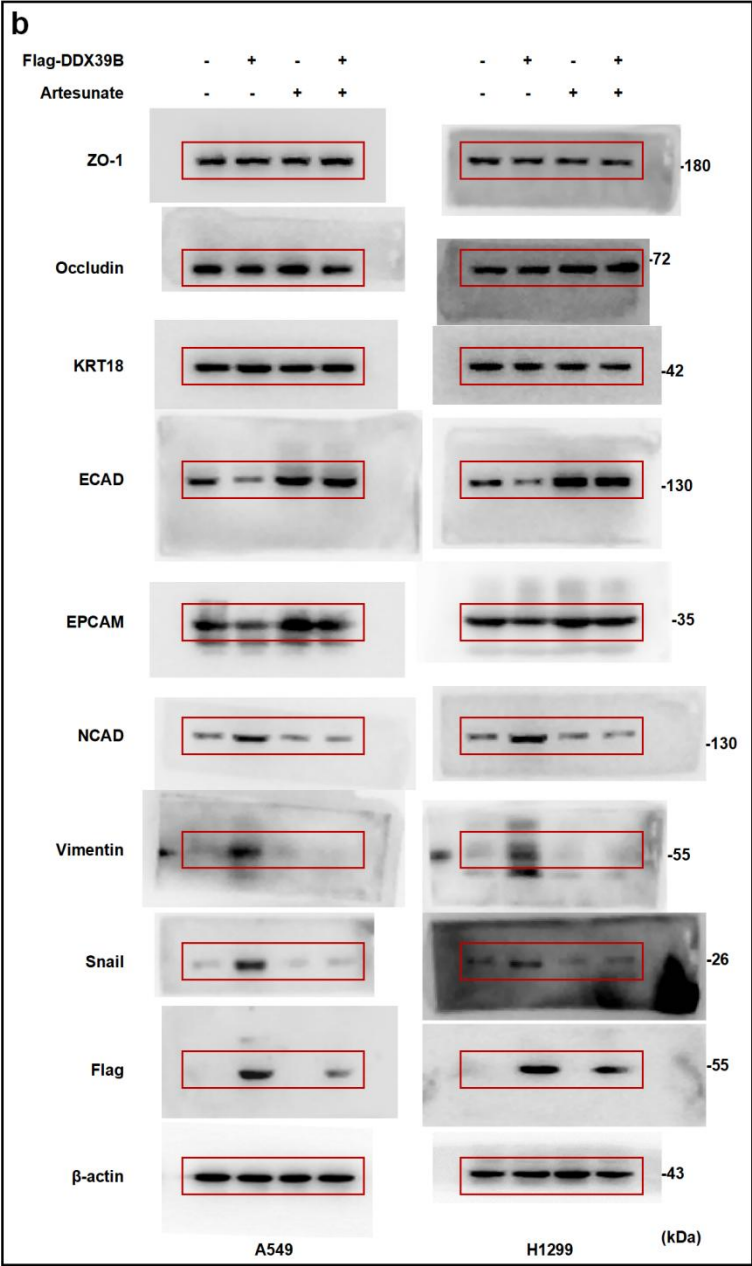

Supplement: Supplementary file 15 — The original and uncropped films of Western blots [file 41392_2025_2305_MOESM15_ESM.pdf]
